# The CD38^+^HLA-DR^+^ T cells with activation and exhaustion characteristics as predictors of severity and mortality in COVID-19 patients

**DOI:** 10.3389/fimmu.2025.1577803

**Published:** 2025-04-30

**Authors:** Qiuyue Long, Shixu Song, Jianbo Xue, Wenyi Yu, Yaolin Zheng, Jiwei Li, Jing Wu, Xiaoyi Hu, Mingzheng Jiang, Hongli Ye, Binghan Zheng, Minghui Wang, Fangfang Wu, Ke Li, Zhancheng Gao, Yali Zheng

**Affiliations:** ^1^ Department of Respiratory, Critical Care and Sleep Medicine, Xiang’an Hospital of Xiamen University, School of Medicine, Xiamen University, Xiamen, China; ^2^ Institute of Chest and Lung Diseases, Xiang’an Hospital of Xiamen University, Xiamen, China; ^3^ Department of Respiratory and Critical Care Medicine, Peking University People’s Hospital, Beijing, China; ^4^ Department of Thoracic Surgery and Oncology, the First Affiliated Hospital of Guangzhou Medical University, State Key Laboratory of Respiratory Disease & National Clinical Research Center for Respiratory Disease, Guangzhou, China; ^5^ Department of Critical Care Medicine, Xiang’an Hospital of Xiamen University, School of Medicine, Xiamen University, Xiamen, China; ^6^ State Key Laboratory of Vaccines for Infectious Diseases, Xiang An Biomedicine Laboratory, Xiamen, China

**Keywords:** COVID-19, CD38+HLA-DR+ T cells, T cells, single-cell transcriptomics, mortality risk factor

## Abstract

**Background:**

The COVID-19 pandemic remains a global health challenge. Severe cases often respond poorly to standard treatments, highlighting the necessity for novel therapeutic targets and early predictive biomarkers.

**Methods:**

We utilized flow cytometry to analyze peripheral immune cells from healthy, bacterial pneumonia patients, and COVID-19 patients. The expansion of activated T cells (CD38^+^HLA-DR^+^), monocytes, and myeloid-derived suppressor cells (MDSCs) were detected and correlated with clinical outcomes to evaluate prognostic potential. The single-cell RNA sequencing (scRNA-seq) was applied to characterize the critical cell subset associated with prognosis and elucidate its phenotype in COVID-19.

**Results:**

We revealed a significant increase in CD38^+^HLA-DR^+^ T cells in non-survivor COVID-19 patients, establishing them as an independent risk factor for 28-day mortality. The scRNA-seq analysis identified the CD38^+^HLA-DR^+^ T cell as a terminally differentiated, Treg-like subset exhibiting both activation and exhaustion characteristics. This subset presented the highest IL-6 and IL-10 mRNA levels among all T-cell subsets. Further functional analysis demonstrated its enhanced major histocompatibility complex class II (MHC-II) cross-signaling and correspondingly enriched cytoskeletal rearrangement processes. In addition, there was dysregulated NAD^+^ metabolism in CD38+HLA-DR+ T cells via scRNA-seq, accompanied by elevated adenosine and decreased NAD^+^ levels in serums from COVID-19 patients.

**Conclusions:**

We identified the selective expansion of CD38^+^HLA-DR^+^ T cells as a novel prognostic indicator for COVID-19 outcomes. These cells’ unique activated-exhausted phenotype, along with their impact on NAD^+^ metabolism, provides new insights into COVID-19 immunopathogenesis.

## 1 Introduction

The pandemic of coronavirus disease 2019 (COVID-19), caused by the SARS-CoV-2 virus, has led to a significant strain on global healthcare systems and has been associated with considerable morbidity and mortality, with over 770 million confirmed cases and more than 7 million deaths worldwide (1). The virus’s high mortality rate and its propensity for mutation have made the development of effective treatments an urgent priority (2). SARS-CoV-2 infection triggers exaggerated inflammatory responses and immune dysregulation, manifesting as systemic inflammatory response syndrome (SIRS), cytokine release storms, multi-organ dysfunction, acute respiratory distress syndrome, etc. (3). The current clinical management of COVID-19 includes a range of interventions, such as oxygen therapy, anti-inflammatory agents, antivirals, and immunotherapy (4, 5). Despite these interventions, severe cases often show poor response to standard treatment regimens, highlighting the need for novel therapeutic targets and early biomarkers for disease prediction in severe COVID-19 patients.

COVID-19 is characterized by immune dysregulation, with various immune cells playing critical roles in disease severity and adverse outcomes (6). For example, T cells expressing activation markers such as CD38 and HLA-DR have been observed in COVID-19 patients, suggesting functional impairment and immune dysregulation associated with disease severity (7, 8). Monocytes, particularly the CD14^+^CD16^+^ subset, are linked to severe COVID-19 due to their production of pro-inflammatory cytokines like IL-6 and TNF-α, which can contribute to cytokine storm and tissue damage (9). MDSCs, which can accumulate in severe COVID-19, may inhibit T cell proliferation and recruit Tregs by secreting IL-10 and TGF-β, contributing to an immunosuppressive state (10, 11). Given immune cells’ high heterogeneity and plasticity, scRNA-seq technology has emerged as a valuable tool for unbiased detection and functional exploration of immune cell subpopulations in infectious diseases (12).

Here, we utilized flow cytometry and scRNA-seq on peripheral blood mononuclear cell (PBMC) samples from COVID-19 patients to investigate the potential of immune cells as prognostic biomarkers and to explore their mechanisms in pathogenesis. Our results showed that the expansion of CD38+HLA-DR+ T cells in peripheral was an informative indicator that correlated with the 28-day mortality. We further identified CD38+HLA-DR+ T cells as a fully differentiated Treg-like T-cell subset exhibiting both activation and exhaustion characteristics. These findings providing an early warning signal for severe cases and potential therapeutic targets for COVID-19’s immunoregulation.

## 2 Methods

### 2.1 Participants recruitment and sample collection

The prospective study was conducted at Xiang’an Hospital of Xiamen University and Peking University People’s Hospital from December 20, 2022 to March 25, 2024 (ChiCTR2100054156, registered 12 October 2021). A total of 58 subjects were enrolled, including 34 COVID-19 patients, 14 bacterial pneumonia patients as disease control, and 10 volunteers as healthy control (**Supplementary Table S1**). Mortality was assessed for all patients included in the study. For COVID-19 patients who died during the observation period, survival time was calculated from hospital admission to the time of death. For patients who survived, survival time was assessed at 28 days after hospital admission. This approach ensured a consistent and standardized evaluation of mortality across all study participants.The diagnosis of COVID-19 was confirmed via positive SARS-CoV-2 nucleic acid tests, and the diagnosis of bacterial pneumonia was confirmed by positive culture results of lower respiratory tract samples. Disease severity of COVID-19 was assessed according to the Diagnosis and Treatment of COVID-19 (Trial Version 10) (13). Peripheral blood samples were collected within three days of admission. PBMCs were isolated from the whole blood by Ficoll-Paque density gradient centrifugation as previously described (14) for flow cytometry and scRNA-seq analysis.

### 2.2 PBMCs isolation

PBMCs were isolated from the whole blood by Ficoll-Paque density (1.077 g/mL) gradient centrifugation as previously described (14). Briefly, blood samples were centrifuged at 2500 rpm for 10 minutes at 20°C to remove plasma. The remaining leucocyte was diluted with an equal volume of phosphate-buffered saline (PBS) and gently layered onto an equal volume of Lymphoprep medium (Cat #18061, STEMCELL) in a 15 mL centrifuge tube, ensuring distinct phase separation. Followed by a centrifugation at 400 g for 20 minutes at 20°C with no brake. The mononuclear cell layer was carefully collected, washed with 5 mL of PBS. After centrifugation, the pellet was resuspended in cryopreservation medium, aliquoted into cryovials, and stored at -80°C until analysis.

### 2.3 Flow cytometry analysis

The cryopreserved PBMCs were thawed at 37°C, diluted in a pre-warmed complete medium (RPMI-1640 ^+^ 10% inactivated FBS), and incubated overnight at 37°C in 5% CO2 before the flow cytometry analysis. We used the Fc Receptor Blocking Solution (422302, BioLegend) to prevent non-specific antibody binding. Then, cells were gently washed with PBS and evenly divided into two aliquots for distinct staining protocols. Activated T cell and its subsets were assessed using the following fluorochrome-conjugated monoclonal antibodies: anti-CD3: BV421, anti-CD4: Percp/cy5.5, anti-CD8: APC, anti-CD38: PE, and HLA-DR: APC-H7. After excluding the lymphocyte linage (lin^+^) population, monocytes were identified by the CD15^-^CD88^+^ gate, and MDSCs were identified by the HLA-DR-CD11b^+^CD33^+^ gate.

In panel one, we evaluated the activation of CD4^+^ and CD8^+^ T cells using CD38 and HLA-DR as activation markers (15). In panel two, we first recognized monocytes using the lin^-^CD88^+^CD15^-^ gate and classified them into classical (cM), non-classical (ncM), and intermediate (iM) monocytes according to CD14 and CD16 markers. Next, we identified myeloid-derived suppressor cells (MDSC) by the lin^-^HLA-DR^low^CD11b^+^CD33^+^ gate, and distinguished two subclusters: monocytic MDSC (M-MDSC) and polymorphonuclear MDSC (PMN-MDSC) based on CD14 and CD15 expression, respectively. **Supplementary Table S2** included the antibodies, fluorochrome conjugates, and clones used for this analysis. After excluding debris, doublets, and dead cells, 10,000 events were sampled for each sample. Representative plots and statistics were generated using FlowJo 10.8.1 software.

### 2.4 Single-cell RNA-seq sequencing and data preprocessing

Single-cell suspensions and libraries were prepared following the manufacturer’s protocols (10 × Genomics). Single-cell sequencing was performed using the Chromium System, which employs droplet sequencing technology to encapsulate individual cells within Gel Beads in Emulsion (GEMs). Each GEM contained a lysed cell, and its transcripts were barcoded with unique molecular identifiers (UMIs). Reverse transcription was initiated in the presence of enzymes, and full-length cDNA was synthesized using free poly-dT as a primer, followed by amplification. The cDNA was then subjected to paired-end sequencing on the NovaSeq platform (CapitalBio Technology). Raw data were processed using the Cell Ranger 7.1.0 pipeline, with reads aligned to the human GRCh38 reference genome under default parameters. Quality control was rigorously applied to the single-cell dataset based on gene detection and mitochondrial content, filtering out cells with fewer than 200 detected genes or more than 90% of the maximum gene number or with mitochondrial UMIs exceeding 25%. Genes detected in at least three cells were retained. Data analysis was conducted using R version 4.2.1.

### 2.5 Principal components analysis and clustering

Gene expression data were scaled using the “ScaleData” function in “Seurat” (16), with a scaling factor of 10,000. The 2,000 most variable genes were identified and subjected to principal component analysis (PCA) using the “vst” method. Then the “ElbowPlot” was used to determine the optimal principal component. Clustering was performed using the Louvain algorithm with a resolution parameter set to 0.6 for general clustering and 1.0 for T cell-specific clustering. Clusters were annotated based on cell-type-specific markers and visualized in a two-dimensional UMAP space. Low-quality clusters were removed based on criteria outlined in **Supplementary Figures S4A–E**, resulting in a final Seurat object containing 45,669 cells for further analysis.

### 2.6 Differential gene expression analysis

Differential expression analysis was conducted using the “FindMarkers” function in “Seurat”, comparing gene expression across various cell types, disease severity of patients, and survival status. The Wilcoxon rank sum test was applied, and genes with adjusted p-values (Padj) < 0.05 were considered significant when setting logfc = 0.25 and min.pct = 0.25. Heatmaps of DEGs were generated using the “plot_heatmap” function in the Scillus package.

### 2.7 Pseudotime trajectory analysis

The Monocle package (17) was used to infer the developmental trajectories of T cells, utilizing highly variable genes for dimension reduction and cell ordering.

### 2.8 Biological pathway enrichment and signature scoring

Utilizing the Gene Ontology (GO) database, we employed the “Scillus” package to conduct pathway and ontology analyses. Specifically, DEGs with a log2 fold-change (log2FC) exceeding 0.25 in distinct T cell types were subjected to biological process enrichment analysis using the “plot_all_cluster_go” function. Furthermore, signature scores were calculated to quantify the expression activities of exhaustion, effector, cytokine, inflammatory, and NAD metabolism among various cell types. This was achieved using the “AUCell_calcAUC” function within the “AUCell” package (18) based on the corresponding gene lists provided in **Supplementary Table S7**.

### 2.9 Transcription factors motif enrichment

To enrich TF motifs among T cell types, we constructed a gene regulatory network using the “SCENIC” package (19) in conjunction with the RcisTarget database. This approach facilitated the prediction of candidate upstream TFs. Subsequently, the activity scores of these TFs were compared across different T cell types and visualized in a heatmap format.

### 2.10 Ligand-receptor interaction analysis

To elucidate potential cell-cell communication pathways, we utilized the CellChat algorithm (20) to identify interactions between CD38^+^HLA-DR^+^ T cells and other PBMC subtypes, resulting in the identification of 15 distinct PBMC cell subtypes. The CellChat database encompasses a comprehensive collection of recognized signaling molecules, including receptor-ligand interactions. The strength of these interactions was estimated by analyzing the average expression levels of immune-related ligands and receptors within each cluster. This analysis was complemented by a permutation test, with significance determined at p < 0.05.

### 2.11 Quantification of plasma NAD^+^ and adenosine levels

NAD^+^ levels in plasma were determined using the NAD^+^ quantitation kit (BC0315, Solarbio). Briefly, 0.1 mL of plasma was mixed with 0.5 mL of acidic extraction solution and boiled for 5 minutes. The mixture was cooled on ice, centrifuged at 10,000 g for 10 minutes at 4°C, and 200 μL of the supernatant was added to an equal volume of alkaline extraction solution. After mixing thoroughly, the sample was centrifuged again under the same conditions, and the supernatant was collected for analysis. Optical density (OD) at 570 nm was measured using a microplate reader (Mutiskan SkyHigh, Thermo Fisher), and NAD^+^ levels were calculated according to the manufacturer’s instructions.

Adenosine levels in plasma were measured using the adenosine ELISA kit (CB12066-Hu, Coibo). Fifty microliters of standard solutions or 5-fold diluted samples were added to respective wells, followed by 50 μL of biotinylated antibody solution. After a 30-minute incubation at 37°C, the wells were washed five times with 350 μL of wash buffer. Subsequently, 100 μL of antibody conjugated with horseradish peroxidase (HRP) was added and incubated for another 30 minutes at 37°C. After washing, 100 μL of color-developing solution was added and incubated for 15 minutes at 37°C in the dark. The reaction was stopped with 50 μL of stop solution, and OD values were measured at 450 nm. Sample concentrations were calculated based on the standard curve (R^2^ > 0.99).

### 2.12 Statistical analysis

Statistical analyses were performed to compare COVID-19 patients with healthy controls and bacterial pneumonia patients, as well as to evaluate differences within the COVID-19 cohort based on disease severity (severe vs. non-severe) and clinical outcomes (survival vs. non-survival). Continuous variables were categorized by distribution: normally distributed variables are reported as mean ± SD, and non-normally distributed as median (interquartile range, IQR). Analyses were performed using GraphPad Prism 9.5.0 and SPSS 26.0. Comparisons of normally distributed variables across groups were made using Student’s t-test and one-way ANOVA, while Mann-Whitney U and Kruskal-Wallis tests were utilized for non-normally distributed variables. Categorical data were expressed as counts or percentages and compared via χ² or Fisher’s exact test as appropriate.

Correlations between continuous variables were evaluated with Pearson’s test (normally distributed) and Spearman’s rho test (non-normally distributed). Univariate and multivariable logistic regression was applied to identify risk factors, with variable selection via stepwise inclusion. Survival analysis was performed using the Kaplan-Meier method, with survival curves compared using log-rank tests. The diagnostic performance was assessed via ROC analysis, determining the optimal cutoff using the Youden index. Statistical significance was defined as *p* < 0.05.

## 3 Results

### 3.1 CD38^+^HLA-DR^+^ T cells expanded specifically in critical COVID-19 patients

**Figure 1:**
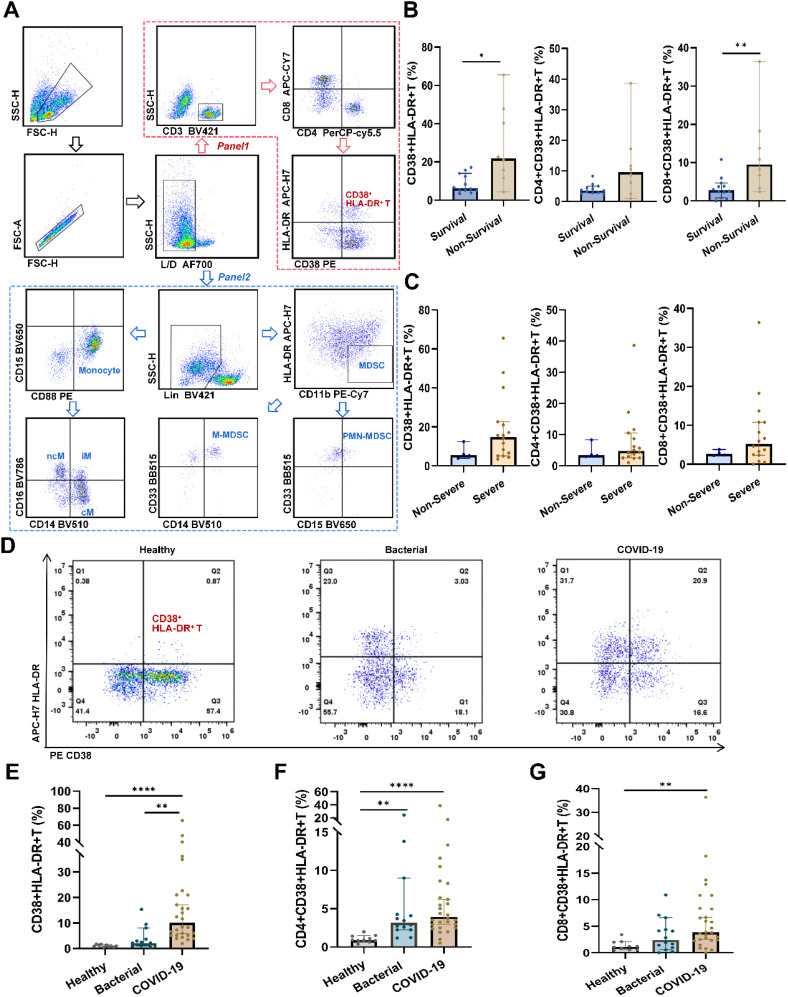
Flow cytometry analysis of peripheral immune cells in pneumonia patients and healthy subjects. **(A)** Representative gating strategy used to identify CD38^+^HLA-DR^+^ T cells, MDSCs, and monocytes. After excluding debris, doublets, and dead cells, the red arrow indicated the gating procedures of CD38^+^HLA-DR^+^ T cells and their subtypes (panel one), and the blue arrow indicated the gating procedures of MDSCs, monocytes, and their subtypes (panel two). **(B)** Percentages of CD38^+^ HLA-DR^+^ T cells and their subclusters (CD4^+^ and CD8^+^) between survival (n = 12) and non-survival patients (n = 8). **(C)** Percentages of CD38^+^ HLA-DR^+^ T cells and their subclusters (CD4^+^ and CD8^+^) between no-severe (n = 4) and severe patients (n = 16). **(D)** Representative flow cytometry plots of CD38^+^HLA-DR^+^ T cells in healthy subjects (n = 10), bacterial pneumonia patients (n = 14), and COVID-19 patients (n = 28). **(E)** Percentages of CD38^+^HLA-DR^+^ T cells in the three groups. **(F)** Percentages of CD4^+^CD38^+^HLA-DR^+^ T cells in the three groups. **(G)** Percentages of CD8^+^CD38^+^HLA-DR^+^ T cells in the three groups. Data were represented as median with 95% CI. Statistical significances were calculated via Mann-Whitney U tests or Kruskal-Wallis tests. *p < 0.05, **p < 0.01, ****p < 0.0001.

First, we analyzed the distribution of activated T cells, MDSCs, and monocytes in PBMC via flow cytometry. Blood samples were collected from 20 COVID-19 patients, including 16 severe and four non-severe cases. Of these, eight out of severe cases deceased during hospitalization. The gating strategies for activated T cells (defined as CD38^+^HLA-DR^+^) and for MDSCs and monocytes, including their subtypes, were shown in **Figure 1A**. No significant increase was observed in MDSCs and monocytes or their subsets in severe or non-survival groups (**Supplementary Figures S1A–D**). However, we found that the CD38^+^HLA-DR^+^ T cell (6.26% vs. 21.80%, *p* = 0.01) and its CD8^+^ subset (2.74% vs. 9.53%, *p* = 0.01) were significantly increased in the non-survival group compared to the survival group (**Figures 1B, C**).

To discern whether the expansion of CD38^+^HLA-DR^+^ T cells is specific to COVID-19 or a general consequence of infection, we extended our investigation to encompass a larger cohort. The final cohort included 28 COVID-19 patients, 14 bacterial pneumonia patients, and 10 healthy subjects. As delineated in **Supplementary Table S1**, no significant demographic differences were observed between patients with bacterial pneumonia and those with COVID-19. Compared to healthy controls and bacterial pneumonia patients, we observed elevated percentages of CD38^+^HLA-DR^+^ T cells in COVID-19 cases (0.94%, 2.16% vs. 10.01%, respectively, *p* < 0.001) (**Figures 1D, E**). Upon further stratification into CD4 and CD8 subsets, while differences among the three groups persisted, direct comparisons between bacterial pneumonia and COVID-19 revealed no significant distinctions (**Figures 1F, G**). Taken together, we identified CD38^+^HLA-DR^+^ T cells as the primary pathogenic cell subset for subsequent analysis.

### 3.2 CD38^+^HLA-DR^+^ T cell as an independent risk factor for 28-day mortality in COVID-19 patients

**Figure 2:**
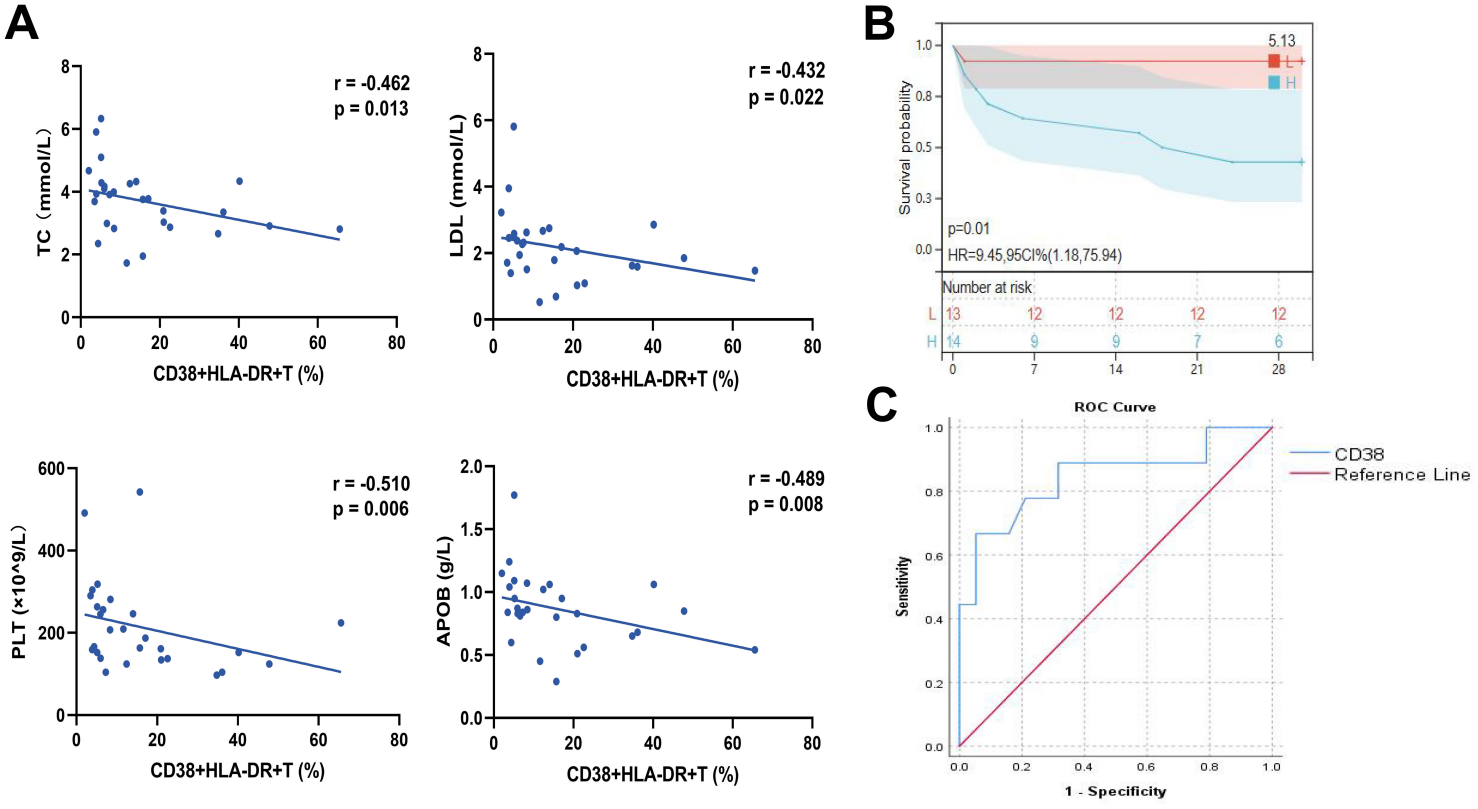
Correlation analysis of clinical parameters and analysis of the prognostic efficacy of CD38+HLA-DR+ T cell percentage in COVID-19 patients. **(A)** The percentages of CD38^+^HLA-DR^+^ T cells were negatively correlated with TC, LDL, APOB, and PLT. **(B)** The Kaplan-Meier curve for 28-day overall survival of COVID-19 patients in groups with high and low CD38^+^HLA-DR^+^ T cell proportions. **(C)** The ROC curve for the predictive accuracy of CD38^+^HLA-DR^+^ T cells for 28-day mortality. Statistical significances of correlation analysis were calculated via Spearman method.

The relationships between the prevalence of CD38^+^HLA-DR^+^ T cells and clinical indicators in COVID-19 patients were first investigated. As shown in **Figure 2A** and **Supplementary Table S3**, the percentages of CD38^+^HLA-DR^+^ T cells were negatively correlated with four parameters: TC, PLT, LDL, and APOB (r = -0.462, r = -0.432, r = -0.510, r = -0.489, respectively, all *p* < 0.05). While the CD4^+^ and CD8^+^ subsets of CD38^+^HLA-DR^+^ T cells showed significant positive correlations with TP (r = 0.441, p = 0.025, r = 0.422, *p* = 0.019, respectively) (**Supplementary Figures S2A, B**). Serum levels of 12 inflammatory cytokines were analyzed in COVID-19 patients, comparing the CD38+HLA-DR+ T^hi^ and the CD38+HLA-DR+ T^low^ group, divided by median (10.01%). The CD38+HLA-DR+ T^hi^ group exhibited higher levels of most cytokines, particularly IL-5 and IFN-α (2.90 pg/ml vs. 4.83 pg/ml, *p* = 0.028, and 3.68 pg/ml vs. 7.73 pg/ml, *p* = 0.028, respectively), with positive correlations between CD38+HLA-DR+ T cell percentages and IL-5/IFN-α levels (*p* = 0.006, r = 0.603; *p* = 0.003, r = 0.637, respectively) (**Supplementary Figures S3A, B**).

We then applied univariate and multivariable regression analyses to elucidate the risk factors associated with 28-day mortality in COVID-19 patients. Most COVID-19 patients were male (21/28, 75%) and elderly (aged over 60, 23/28, 82.14%). Demographic characteristics were presented in **Supplementary Table S4**. No significant differences were observed in age, gender, or past medical histories between the non-survival and survival COVID-19 groups.

The univariate analysis identified CD38^+^HLA-DR^+^ T cells (OR: 1.12, 95% CI: 1.02-1.23, *p* = 0.016), TC (OR: 0.11, 95% CI: 0.02-0.60, *p* = 0.010), LDL (OR: 0.06, 95% CI: 0.01-0.53, *p* = 0.012), and APOB (OR: 0.00, 95% CI: 0.00-0.08, *p* = 0.008) as being significantly associated with 28-day mortality in COVID-19 patients (**Table 1**). A multivariable regression analysis was further conducted. The results demonstrated that the percentage of CD38^+^HLA-DR^+^ T cells (OR: 1.13, 95% CI: 1.01-1.26, *p* = 0.039) was an independent risk factor for death in COVID-19 Patients. Kaplan-Meier survival analysis further verified that the 28-day survival rate was higher in the CD38^+^HLA-DR^+^ T^hi^ group than in the CD38^+^HLA-DR^+^ T^low^ group, as divided by the median of CD38^+^HLA-DR^+^ T cell counts (*p* < 0.05) (**Figure 2B**).

**Table 1:** Analysis of risk factors for mortality of patients with COVID-19.

Parameters	Univariate OR (95%CI)	*p*-value	Multivariate OR (95%CI)	*p*-value
CD38^+^HLA-DR^+^T (%)	1.12 (1.02-1.23)	0.016×	1.13 (1.01-1.26)	0.039×
Age (years)	1.06 (0.99-1.15)	0.103		
Gender	–	0.994		
TP (g/L)	1.04 (0.93-1.16)	0.530		
ALB (g/L)	0.86 (0.72-1.04)	0.121		
GLO (g/L)	1.11 (0.98-1.26)	0.104		
TBIL (μmol/L)	1.10 (0.97-1.25)	0.124		
DBIL (μmol/L)	1.17 (0.98-1.40)	0.080		
ALT (U/L)	0.99 (0.96-1.02)	0.423		
AST (U/L)	1.00 (0.98-1.02)	0.738		
γ-GT (U/L)	0.98 (0.96-1.01)	0.187		
ALP (U/L)	1.00 (0.98-1.02)	0.796		
TG (mmol/L)	0.55 (0.16-1.94)	0.352		
TC (mmol/L)	0.11 (0.02-0.60)	0.010×	0.86 (0.01-51.62)	0.943
HDL (mmol/L)	0.11 (0.01-1.79)	0.120		
LDL (mmol/L)	0.06 (0.01-0.53)	0.012×	0.76 (0.00-2072.28)	0.947
APOA (g/L)	0.17 (0.01-2.83)	0.217		
APOB (g/L)	0.00 (0.00-0.08)	0.008×	0.00 (0.00-4764152.30)	0.492
CK (U/L)	1.00 (1.00-1.01)	0.141		
LDH (U/L)	0.99 (0.99-1.00)	0.139		
WBC (×10^9^/L)	1.05 (0.83-1.31)	0.702		
NEUT (×10^9^/L)	1.06 (0.83-1.35)	0.662		
LYM (×10^9^/L)	0.68 (0.08-5.80)	0.721		
MONO (×10^9^/L)	0.71 (0.20-2.51)	0.593		
HGB (×10^9^/L)	1.00 (0.97-1.04)	0.837		
PLT (×10^9^/L)	1.00 (0.99-1.01)	0.622		
IL-6 (pg/ml)	1.01 (1.00-1.01)	0.086		
IL-10 (pg/ml)	1.05 (0.95-1.15)	0.364		

OR, Odds ratio; CI, Confidence interval; –, Not applicable.

To assess the predictive ability of the percentage of CD38^+^HLA-DR^+^ T cells for patient survival outcomes, we performed a ROC curve analysis. The area under the curve (AUC) was 0.845 (95% CI: 0.67-1.00), indicating a pronounced predictive ability of CD38 proportion in distinguishing between survival and non-survival outcomes (**Figure 2C**). The optimal cutoff value, determined by the maximum Youden index of 0.614, was identified as 20.95. At this threshold, the sensitivity was 0.667, and the specificity was 0.947. Overall, these results indicated the prognostic potential of the CD38+HLA-DR+ T subset in COVID-19.

### 3.3 The CD38^+^HLA-DR^+^ T cell was an exhausted, Treg-like subset with high IL-6 and IL-10 mRNA expression

**Figure 3:**
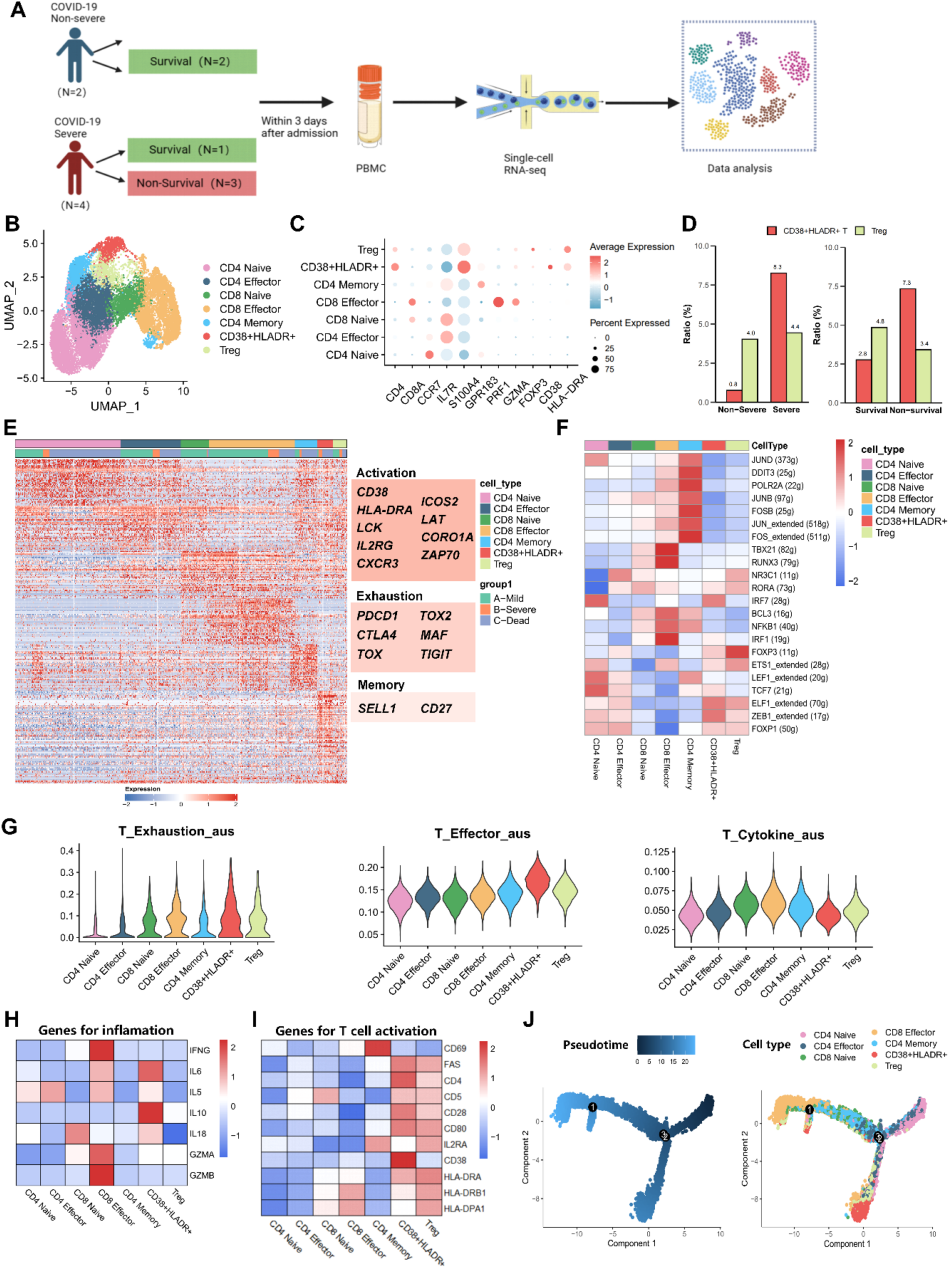
Characterization of CD38^+^HLA-DR^+^ T Cells as an exhausted Treg-Like Subset via single-cell transcriptome. **(A)** Schematic flowchart of PBMC sampling and single-cell RNA sequencing. **(B)** Two-dimensional UMAP plot depicting the re-clustering of 15,908 T cells into seven distinct subtypes, including CD4 Naive, CD4 Effector, CD4 Memory, CD8 Naive, CD8 Effector, Treg, and CD38^+^HLA-DR^+^ T cells. **(C)** Dot plot showing marker genes (bottom row) used to identify the seven T cell subsets (left column). **(D)** Bar graphs illustrating the relative proportions of CD38^+^HLA-DR^+^ T cells between the severe and non-severe groups (left panel) and between non-survival and survival groups (right panel). **(E)** Heatmap showing differentially expressed genes (DEGs) across each T cell subtype, with CD38^+^HLA-DR^+^ T cells exhibiting a transcriptomic landscape similar to Tregs. Genes indicating activation and exhaustion were highly expressed in the CD38^+^HLA-DR^+^ T cells. **(F)** Heatmap illustrating the activity scores of transcription factors (TFs) showing comparable profiles between CD38^+^HLA-DR^+^ T cells and Tregs. **(G)** The biological signature scores of T-cell exhaustion, effector, and cytokine across seven T-cell subclusters. **(H)** Heatmap cytokine storm-associated genes in each T cell subset. **(I)** Heatmap of T-cell activation marker genes across each T subcluster. **(J)** Differentiation trajectories of T cells, with color-coding to indicate the level of differentiation (left) and T-cell subtypes (right).

We further applied scRNA-seq to clarify the pathological function of CD38^+^HLA-DR^+^ T cells. Six COVID-19 patients were enrolled, including four severe and two non-severe patients (**Figure 3A**, **Supplementary Table S5**). Three of them died during hospitalization. After rigorous quality control to exclude the doublets and low-quality transcripts (**Supplementary Figures S4A–D**), a total of 45,669 PBMCs were retained for downstream analysis. Unsupervised clustering analyses were conducted, the uniform manifold approximation and projection (UMAP) yielded 23 clusters. Through manual annotation based on known marker genes, the 23 clusters were incorporated into nine distinct cell types: T cells (CD3D), B cells (CD79A and MS4A1), plasma cells (MZB1), CD14 monocytes (CD14), NK cells (NKG7 and KLRD1), CD14CD16 monocytes (CD14 and FCGR3A), CD16 monocytes (FCGR3A), DC cells (CD1C and TCF4), and platelets (PPBP) (**Supplementary Figures S5A–D**). We then depicted immune cell atlas with different disease severities and outcomes. Consistent with previous reports (21) of lymphopenia in COVID-19, our results showed that the percentage of T cells was lower in severe patients (about 29%) than in non-severe patients (about 45%) (**Supplementary Figure S5E**). A similar decrease was observed in the group of non-survival patients (about 31%) compared to survival patients (about 38%). NK cells decreased from 28% in the non-severe to 13% in the severe and from 26% in the survival to 10% in the non-survival, also contributing to the lymphopenia. As for monocytes, there were remarkable elevations in both the CD14 classical cluster and the CD14CD16 intermediate cluster, possibly associated with a more severe inflammatory storm (22).

To explore the possible pathological mechanism of CD38^+^HLA-DR^+^ T cells, we re-clustered 15,908 T cells and yielded seven subclusters via UMAP: CD4 Naive (CD4 and CCR7), CD4 Effector (CD4, IL7R, and GPR183), CD4 Memory (CD4, S100A4, and GPR183), CD8 Naive (CD8A and IL7R), CD8 Effector (CD8A, PRF1, and GZMA), Treg (CD4 and FOXP3), and CD38^+^HLA-DR^+^ (CD38 and HLA-DR) T cells (**Figures 3B, C**). We analyzed the relative percentage of each T-cell subcluster in patients with different disease severity and outcomes (**Supplementary Figures S6A, B**). In line with our results from flow cytometry, an expansion in the mean percentage of CD38^+^HLA-DR^+^ T cells occurred in severe patients (8.3%) compared to non-severe patients (0.8%) and in the non-survival group (7.3%) compared to the survival group (2.8%). While there was no significant change in the percentage of Tregs. (**Figure 3D**). Next, we compared differentially expressed genes (DEGs) between CD38^+^HLA-DR^+^ T cells and other T-cell subclusters. Molecules associated with T cell activation (CD38, HLA-DR, LCK, IL2RG, etc.), exhaustion (PDCD1, CTLA4, TIGIT, TOX, TOX2, etc.), and memory (SELL1 and CD27) were highly expressed in CD38^+^HLA-DR^+^ T cells (23) (**Figure 3E**, **Supplementary Table S6**), revealing the exhausted characteristic of this subset. Intriguingly, the heatmap of DEGs showed that CD38^+^HLA-DR^+^ T cells and Tregs possessed similar transcriptome profiles (**Figure 3E**) and shared the most DEGs (**Supplementary Figure S4C**). Transcription factor (TF) profiles also revealed similar expression patterns between the CD38^+^HLA-DR^+^ T cell and Tregs, in both, upregulated TF activities included FOXP3, ZEB1, ELF1, ETS1, and FOXP1 were observed (**Figure 3F**). This potentially interpreted that the CD38^+^HLA-DR^+^ T cell was a Treg-like subset. Besides, compared all other T-cell subtypes, the CD38^+^HLA-DR^+^ T cells exhibited higher expression levels of interferon regulatory factors (IRF1 and IRF7) and T cell factor -7 (TCF7), indicating the host’s immune response against viral infection (24, 25).

We further evaluated the biological functions by comparing the scores of exhaustion, activation (effector), cytokine production among T-cell subtypes. The signature gene sets (22, 26) used for functional analysis were listed in **Supplementary Table S7**. There were highest scores of exhaustion and activation, as well as a relatively low score of cytokine production in CD38^+^HLA-DR^+^ T cells (**Figure 3G**), once indicating its exhaustion and dysfunction. In addition, subsequent analysis revealed the highest expression of IL-6 and IL-10 in the CD38^+^HLA-DR^+^ T subset (**Figure 3H**). And both CD38^+^HLA-DR^+^ T cells and Tregs were mainly characterized by the late-stage activation marker gene (FAS, also known as CD95) rather than the early (CD69) (**Figure 3I**). This was in line with pseudo-time results, in which they were at the terminal differentiation stage, specifically the CD38^+^HLA-DR^+^ T subset (**Figure 3J**). Collectively, the above results indicated that the CD38^+^HLA-DR^+^ T cell was an exhausted Treg-like subset with high IL-6 and IL-10 expression.

### 3.4 CD38^+^HLA-DR^+^ T cells were involved in MHC-II cross-talk and cytoskeletal rearrangements

**Figure 4:**
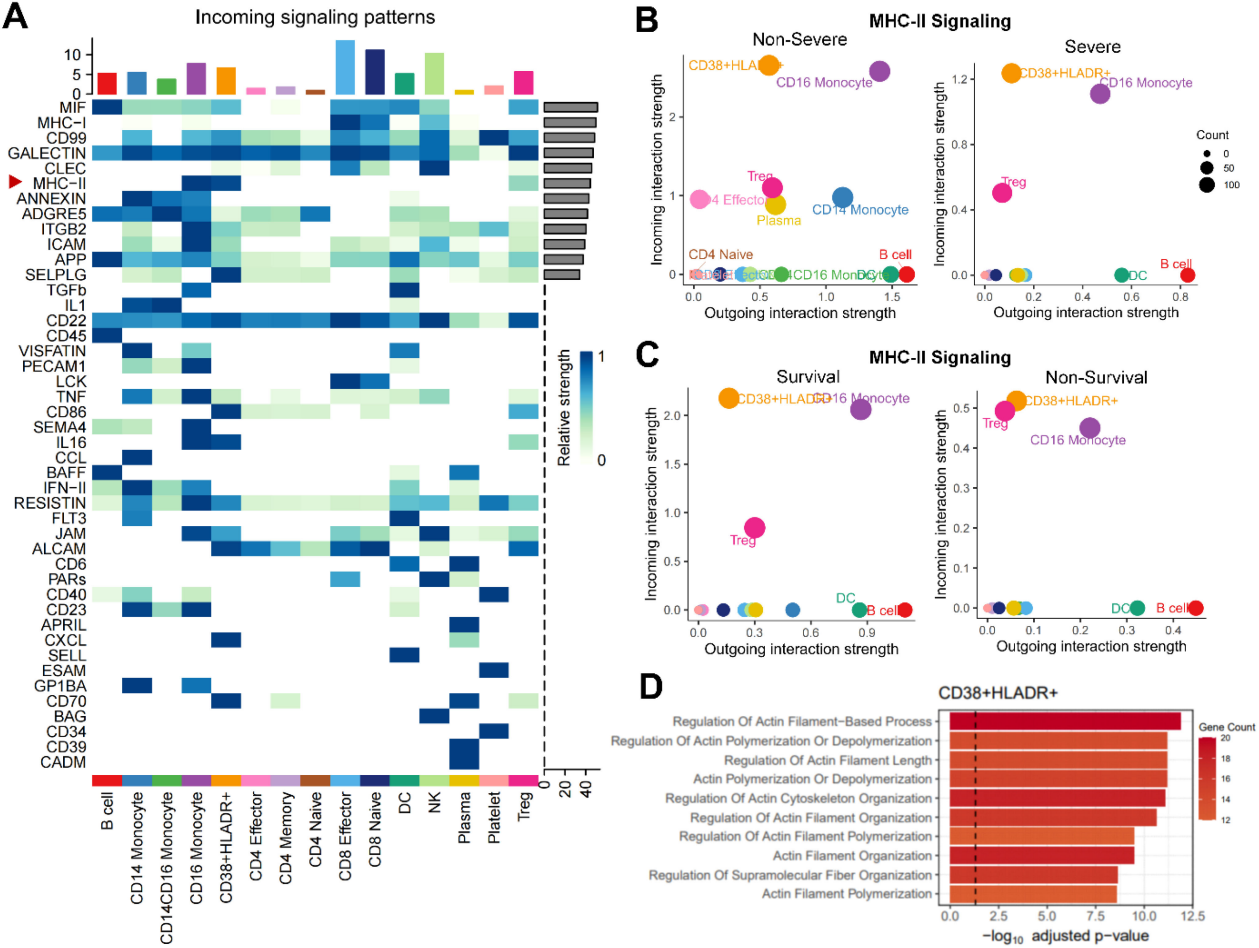
Enhanced MHC-II signaling and enriched cytoskeletal rearrangement process in CD38^+^HLA-DR^+^ T cells. **(A)** Heatmap depicting overall incoming signalings of 15 cell types in PBMCs. B-C. Bubble plots showing that the CD38^+^HLA-DR^+^ T cells received the most vital stimulus of MHC-II signal regardless of **(B)** disease severity and **(C)** clinical outcomes. **(D)** Bar plot showing significantly enriched GO terms associated with biological functions in CD38^+^HLA-DR^+^ T cells.

We then applied Cellchat to analyze cell-cell communications between CD38^+^HLA-DR^+^ T cells and other PBMC cell types. Among overall incoming signaling patterns, there was close MHC-II cross-talk in the CD38^+^HLA-DR^+^ T subset (**Figure 4A**). Regarding MHC-II signaling, which involves necessary molecules for exogenous antigen presentation during T cell activation processes (27), the CD38^+^HLA-DR^+^ T subset exhibited the strongest incoming signaling with antigen presenting cells (APCs) among all T-cell subtypes regardless of disease severity and outcomes (**Figures 4B, C**), followed by Tregs, preliminarily suggesting they experienced overwhelmingly activated stimulation. Next, DEGs between CD38^+^HLA-DR^+^ T cells and other T cell subtypes were applied to pathway analysis based on the GO database. As shown in **Figure 4D**, the CD38^+^HLA-DR^+^ T subset was predominantly involved in cytoskeletal motility pathways such as actin filament polymerization and supramolecular organization, which was closely related to immune synapse formation during T-cell activation process (28). Taken together, the response of T cell receptors to excessive MHC-II stimulation might cause drastic cytoskeleton alterations cytoskeleton and subsequent overactivation in CD38+HLA-DR+ T cells.

### 3.5 Dysregulated NAD^+^ metabolism in COVID-19 patients

**Figure 5:**
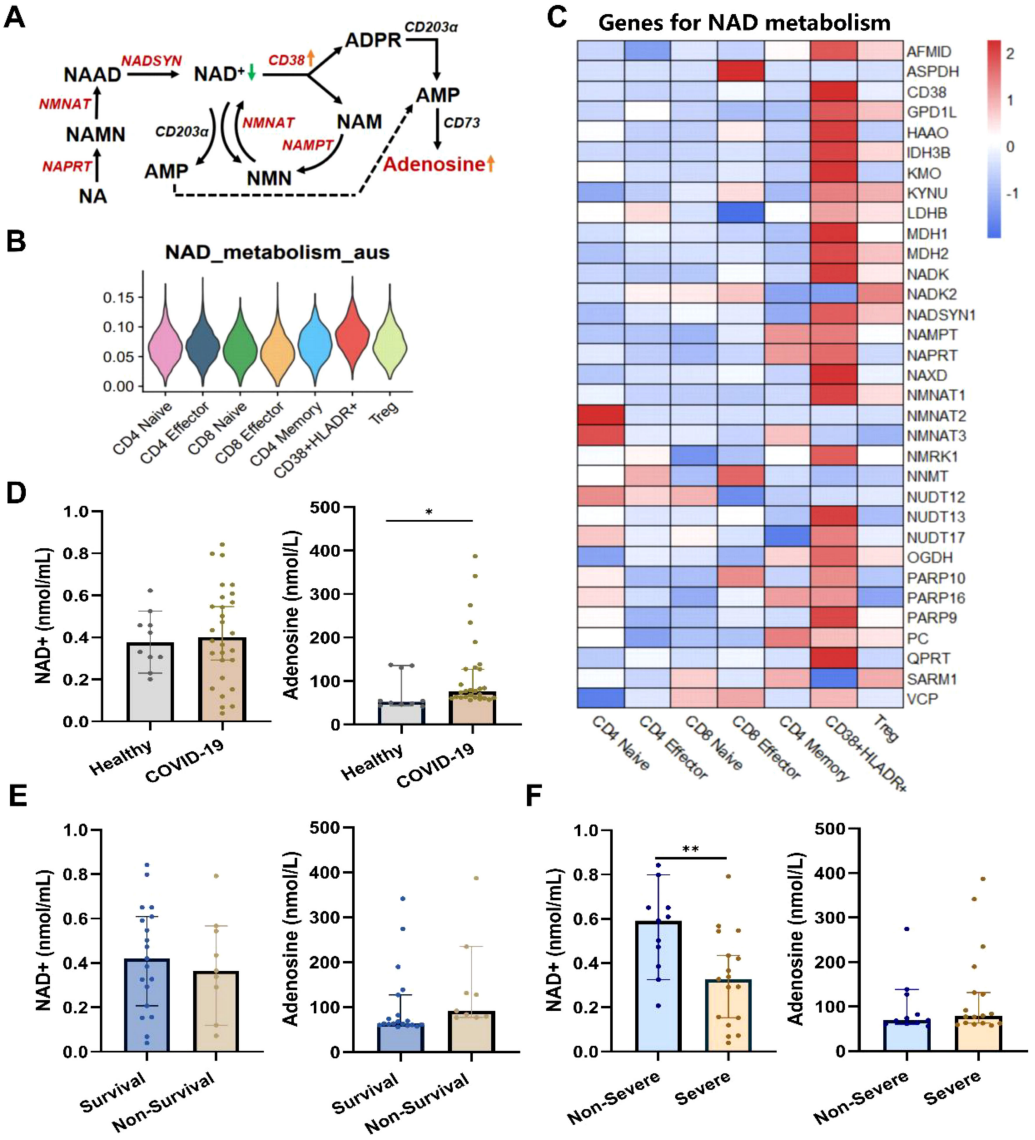
Dysregulated NAD^+^ metabolism in CD38^+^HLA-DR^+^ T cells and COVID-19 patients. **(A)** Dysregulates NAD^+^ metabolism in CD38^+^HLA-DR^+^ T cells identified through scRNA data. The red color indicated upregulated NAD^+^ metabolism-associated enzymes. **(B)** The enriched scores of the NAD^+^ metabolism process across seven T subclusters. **(C)** Heatmap of genes involved in NAD^+^ metabolism across each T subcluster. **(D)** The serum NAD^+^ and adenosine levels between healthy (n =10) and COVID-19 (n = 28) groups. **(E)** The serum NAD^+^ and adenosine levels between survival (n =19) and non-survival (n =9) COVID-19 patients. **(F)** The serum NAD^+^ and adenosine levels between non-severe (n =11) and severe (n =17) COVID-19 patients. *p < 0.05, **p < 0.01. Data were represented as median with 95% CI. Statistical significances were calculated via Mann-Whitney U tests.

The CD38 is a major NAD^+^ glycolytic enzyme (NADase) in mammals (29). The active CD38 enzyme activity, which translated the NAD^+^ into adenosine (**Figure 5A**), was associated with T cell exhaustion through adenosine receptor signaling (30). Thus, we assessed the NAD^+^ metabolism activities in T-cell subtypes based on relevant gene dataset. The CD38^+^HLA-DR^+^ T cell had the highest scores of NAD^+^ metabolism among all T cell subclusters (**Figure 5B**). Detailed expressions of NAD^+^ metabolism-related genes were shown in **Figure 5C**. To verify the dysregulated NAD^+^ metabolism in COVID-19 cases, we subsequently examined NAD^+^ and adenosine levels in clinical plasma samples (**Figures 5D–F**). The NAD^+^ level was markedly lower in severe COVID-19 patients compared to non-severe patients (0.591 vs. 0.337 nmol/mL, *p* < 0.05), although no significant difference was observed between COVID-19 patients and healthy subjects. As expected, the adenosine level was significantly elevated in COVID-19 patients compared to healthy controls (76.93 pmol/mL vs. 52.32 pmol/mL, *p* < 0.05), while no significant differences were observed in disease severity or outcome groups.

## 4 Discussion

In present study, we identified a unique T cell subset, the CD38^+^HLA-DR^+^ T cells, that could serve as an independent risk factor of 28-day mortality in COVID-19 patients. This targeted subset was presented characteristics of activation and exhaustion and a similar transcriptome profile with Treg, with high levels of IL-6 and IL-10 transcripts. Corresponding to the strong incoming MHC-II signaling, cytoskeletal rearrangement processes relevant to T-cell activation were enriched in this overactivated subset. Furthermore, CD38 may act as an NADase and contribute to the generation of pathological CD38^+^HLA-DR^+^ T cells in the context of COVID-19. Our findings highlight the critical role of the CD38^+^HLA-DR^+^ T cell subset in predicting the early prognosis of COVID-19 patients. The highly-activated but exhausted T cell subset may be a novel therapeutic target to improve patients’ outcomes.

Our study reveals a significant increase in CD38^+^HLA-DR^+^ T cells in severe COVID-19 patients. This aligns with the observations in virus infections and autoimmune diseases (31, 32). CD38, a multifaceted glycoprotein with roles in cell adhesion, signal transduction, and calcium signaling, is prominently expressed on immune cells such as CD4^+^, CD8^+^ T cells, B lymphocytes, and natural killer cells (33, 34). Its co-expression with HLA-DR, an MHC-II receptor pivotal for antigen presentation to T cells (35), indicates an activated state of immune response modulation. Studies have noted increased CD38^+^HLA-DR^+^CD8^+^ T cells in H7N9 influenza and COVID-19, particularly in critically ill cases, suggesting their prognostic value and involvement in severe immune dysregulation (36). Additionally, the accumulation of this subset with disease progression is believed to exert cytotoxic effects, potentially contributing to tissue damage. In systemic lupus erythematosus, the upregulation of CD38 and HLA-DR on CD4^+^ and CD8^+^ T cells signifies a state of chronic immune activation (37). These collective findings underscore the integral role of CD38^+^HLA-DR^+^ T cells in immune activation and disease pathogenesis.

Through single-cell transcriptome analysis, we revealed the simultaneous state of activation and exhaustion in CD38^+^HLA-DR^+^ T cells in COVID-19. The persistent activation status is consistent with previous reports, potentially driven by the ongoing antigen presentation and immune system overactivation observed in COVID-19 (8). The high expression of tyrosine kinases LCK (38) and ZAP70 (39) are crucial for mediating T cell activation and signal transduction, further emphasizing the cells’ initial robust response to the viral antigen. However, this hyperactivation may precipitate a cascade of inflammatory responses, potentially culminating in immunopathology and a more severe disease course (40). In chronic viral infections like HIV (41) and hepatitis C (31), the immune exhaustion exhibited by CD38^+^HLA-DR^+^ T cells has been observed, and is manifested by diminished proliferation and effector function. The onset of exhaustion in CD38^+^HLA-DR^+^ T cells appears to be a common mechanism among various viral infections, where sustained antigenic stress leads to T cell dysfunction, leading to a compromised antiviral response and may contribute to disease progression (42). This T cell subset may become common therapeutic targets that could be exploited to restore T cell function and modulate the immune response in a range of viral diseases.

Furthermore, we revealed that the CD38^+^HLA-DR^+^ T cells exhibit high levels of transcripts encoding IL-6 and IL-10. IL-6, primarily secreted by monocytes and macrophages, is recognized as a vital driver of the cytokine storm in COVID-19 patients, exacerbating disease progression and leading to more severe symptoms and higher mortality (43, 44). IL-6 antagonists are widely accepted as therapeutic regimens for severe COVID-19, significantly mitigating the cytokine storm (45). Conversely, IL-10, typically known for its anti-inflammatory properties, is also highly expressed in CD38^+^HLA-DR^+^ T cells in our research. The elevated IL-10 could initially be protective by mitigating the excessive inflammatory damage. While persistently high IL-10 levels could also tip the balance towards immunosuppression, potentially impairing the host’s ability to clear pathogens effectively (46). Understanding this intricate balance in CD38^+^HLA-DR^+^ T cells is crucial for developing immunomodulatory strategies in COVID-19. To translate these findings into clinical practice, it is proposed that CD38^+^HLA-DR^+^ T cell levels could be routinely measured using flow cytometry upon hospital admission. This approach would enable the early identification of high-risk patients, facilitating timely interventions such as immunomodulatory therapies. By integrating CD38^+^HLA-DR^+^ T cell monitoring into clinical workflows, risk stratification can be enhanced, personalized treatment decisions can be guided, and patient prognosis may ultimately be improved.

Of note, at single-cell resolution, we found that CD38^+^HLA-DR^+^ T cells were Treg-like regarding transcription factors and transcriptional profiles. Both T-cell subsets were marked by late-stage rather than early activation genes and by terminal differentiation stages. The most significant DEGs between Tregs and CD38^+^HLA-DR^+^ T cells, including *PCLAF*, *CD38*, *PLAC8*, *IGFBP4*, and *STMN1*, offer insights into their molecular signatures and potential roles in immune regulation. The high similarity and intricate interplay between CD38^+^HLA-DR^+^ T cells and Tregs in COVID-19 highlights the complexity of immune dysregulation. Future immunological research is warranted to dissect their functional overlap and divergence in COVID-19.

CD38, which is also recognized as a pivotal NAD^+^ hydrolase, may play a significant role in the immunosuppressive environment characteristic of COVID-19 through its enzymatic conversion of NAD^+^ to adenosine (30). Our research has revealed disruptions in NAD^+^ metabolism in COVID-19 patients, as evidenced by transcriptome analysis highlighting an enrichment of the NAD^+^ metabolic pathway and by metabolite alterations in peripheral blood. NAD^+^ is crucial for cellular energy metabolism (47) and immune cell functions, including macrophage activation and T cell subset survival and polarization (48). This underscores the importance of NAD^+^ in maintaining the equilibrium of immune responses. Consistent with our findings, previous studies have reported NAD^+^ depletion in viral infections included HIV-1, HSV-1, and COVID-19 (49–51). Studies have tentatively proved the therapeutic benefits of NAD^+^ supplements. In preclinical models, supplementing NAD^+^ precursor nicotinamide riboside (NR) or nicotinamide mononucleotide (NMN) could restore the NAD^+^ level and improve recovery from SARS-CoV-2 infection (52). A clinical trial also shows that supplementation with a combined metabolic activator consisting of NAD^+^ precursors accelerates disease recovery in COVID-19 (53). Taken together, CD38 may induce distinct downstream effects in CD38^+^HLA-DR^+^ T cells, thus inducing their immunopathological profile. The functional effects and molecular mechanisms mediated by CD38-NAD+ metabolic axis in CD38^+^HLA-DR^+^ T cells warrant further investigation.

There are some limitations in the research. First, our data’s relatively small sample size and cross-sectional nature may limit the findings’ generalizability to broader populations. Future studies should aim to include larger and longitudinal cohorts to validate our findings and ensure they are representative of the wider population. Second, the phenotypic analysis of CD38+ HLA-DR+ T cells in this study was primarily based on transcriptomic data. Future functional assays should employ *in vitro* and *in vivo* models dissect the nature of this subset. Lastly, the therapeutic implications of targeting CD38+HLA-DR+ T cells or the CD38-NAD+ axis require further investigation in preclinical and clinical trials to assess safety, efficacy, and optimal dosing strategies.

In conclusion, the activation and exhaustion of CD38^+^HLA-DR^+^ T cells in COVID-19 represent a complex equilibrium central to the disease’s pathogenesis. Understanding the mechanisms that drive this balance is vital for developing therapeutic strategies to restore T-cell function and modulate the immune response to improve patient outcomes.

## Data Availability

The scRNA seq data presented in the study are deposited in the GSA-Human repository of the National Bioinformatics Center of China, accession number HRA011282, https://ngdc.cncb.ac.cn/gsa-human/browse/HRA011282.
