# Supplementary material for: The CD38+HLA-DR+ T cells with activation and exhaustion characteristics as predictors of severity and mortality in COVID-19 patients
Source: Front Immunol. 2025 Apr 30;16:1577803. doi: 10.3389/fimmu.2025.1577803 (PMC12074963; doi:10.3389/fimmu.2025.1577803)
Supplement: Supplementary file 1 [file DataSheet1.docx]

Supplementary Material

# Supplementary Figures

**
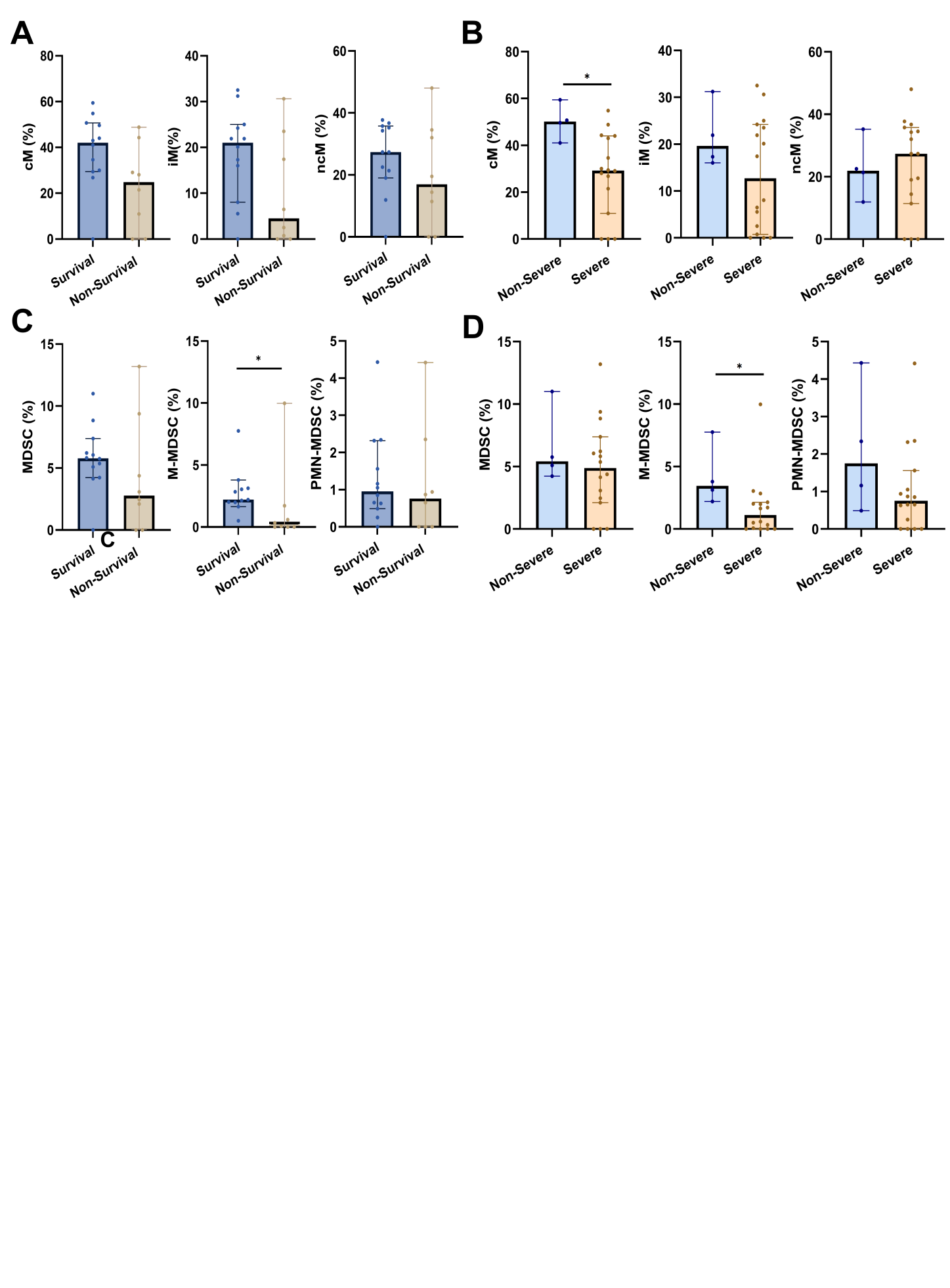
**

**Supplementary Figure 1.** A. Percentages of CD14^+^ classical monocytes (cM), CD14^+^CD16^+^ intermediate monocytes (iM), and CD16^+^ non-classical monocytes (ncM) between survival (n = 12) and non-survival patients (n = 8). B. Percentages of CD14^+^ cM and CD14^+^CD16^+^ iM and CD16^+^ ncM monocytes between no-severe (n = 4) and severe patients (n = 16). C. Percentages of MDSCs, monocytic MDSCs (M-MDSCs), and polymorphonuclear MDSCs (PMN-MDSCs) between survival and non-survival patients. D. Percentages of MDSCs, M-MDSCs, and PMN-MDSCs between no-(CD4^+^ and CD8^+^) between severe and severe patients.


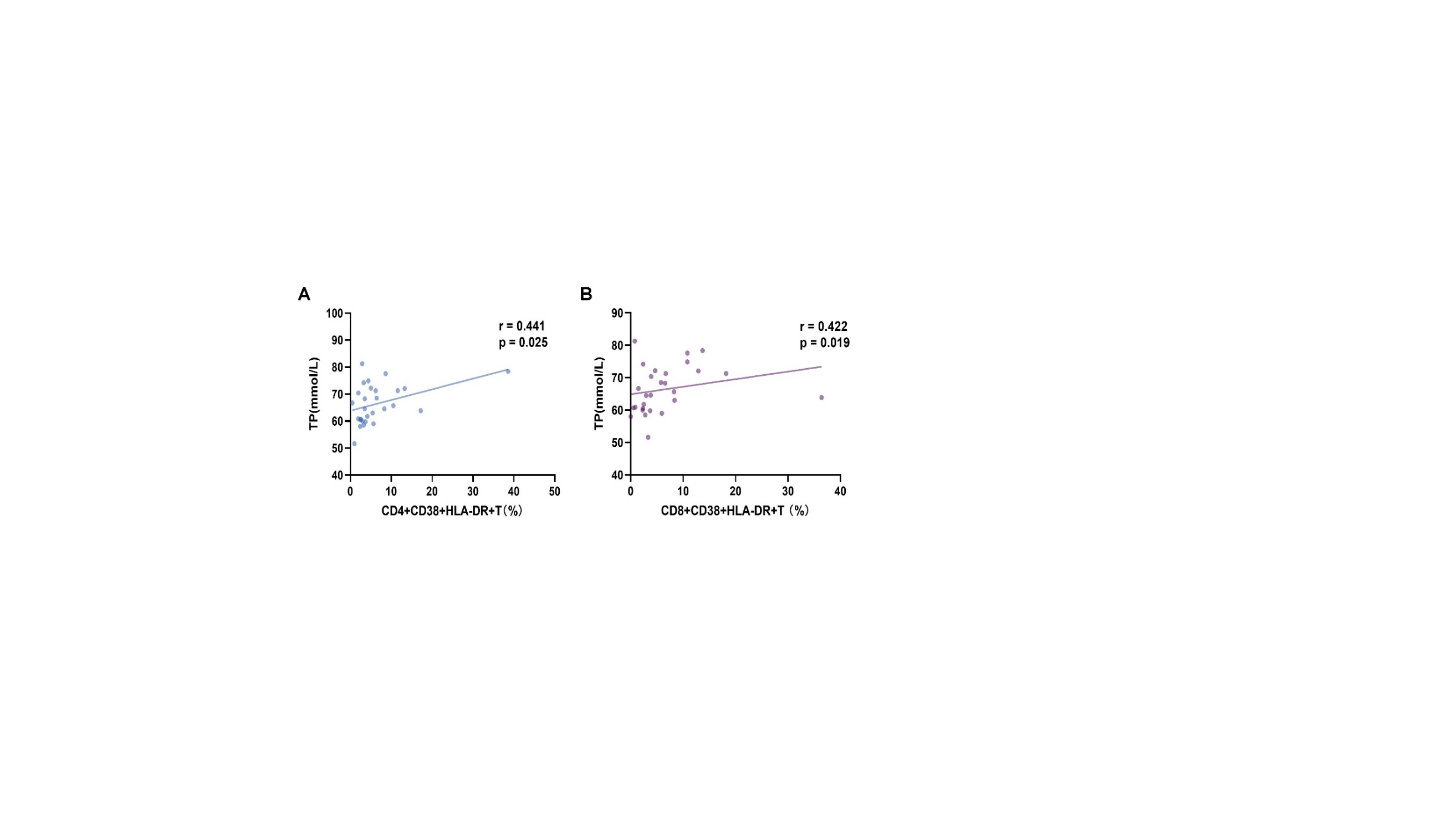
**Supplementary Figure 2.** Spearman correlation analysis between the percentage of CD4^+^ and CD8^+^CD38^+^HLA-DR^+^ T cell subsets and clinical indicators. A. The percentage of CD4^+^CD38^+^HLA-DR^+^ T cells is positively correlated with TP (r = 0.441, *p* = 0.025). B. The percentage of CD8^+^ CD38^+^HLA-DR^+^ T cells is positively correlated with TP (r = 0.422, *p* = 0.019).


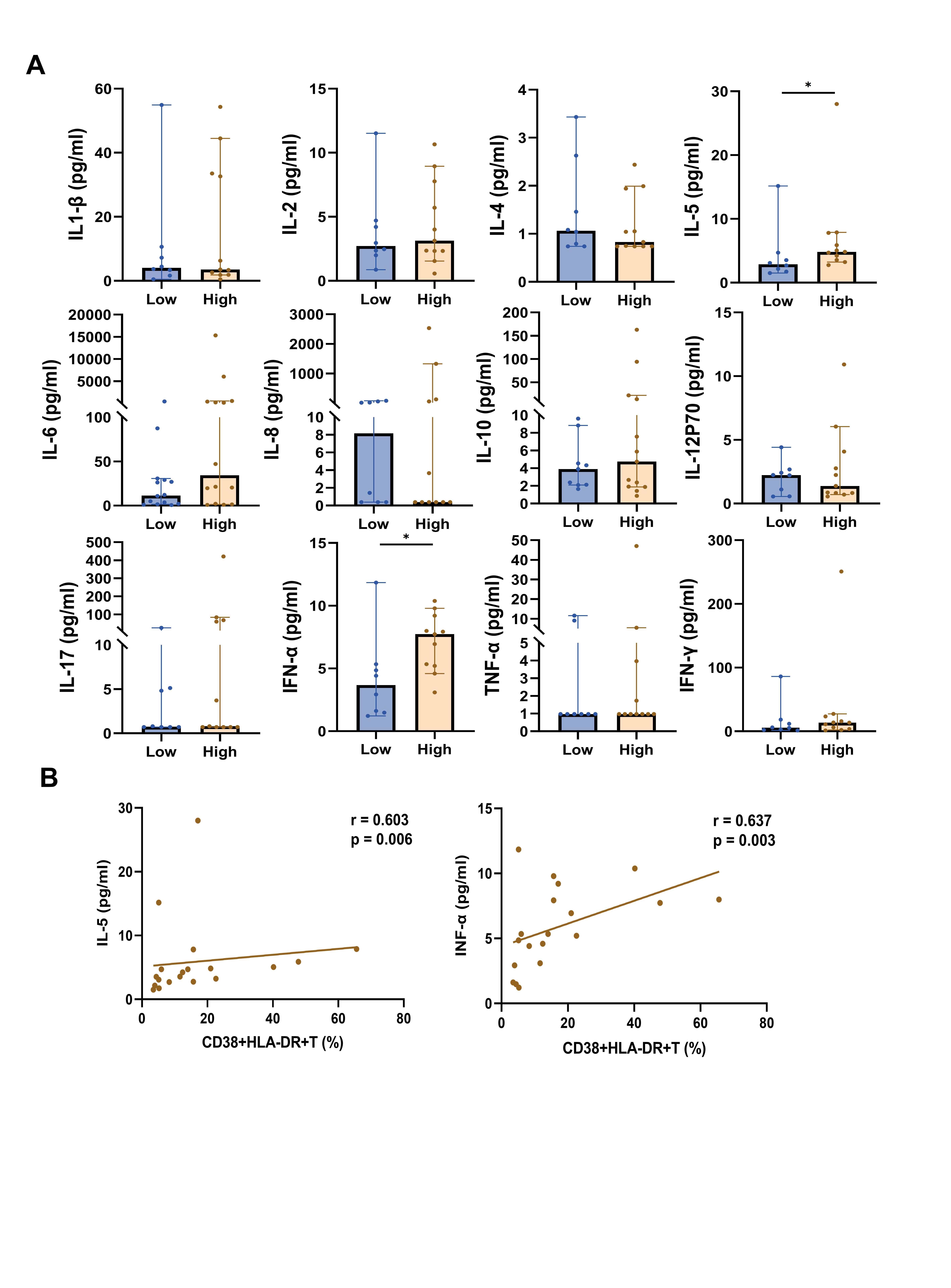


**Supplementary Figure 3.** Correlation analysis between clinical parameters and CD38^+^HLA-DR^+^ T cell prevalence in COVID-19 patients**.** A. Serum levels of twelve inflammatory cytokines in COVID-19 patients stratified by the median of CD38^+^HLA-DR^+^ T cell percentage into high and low groups. B. The percentages of CD38^+^HLA-DR^+^ T cells were positively correlated with IL-5 and IFN-α. Data were represented as median with 95% CI. Statistical significances were calculated via Mann-Whitney U tests or Spearman's rho test. **p* < 0.05.


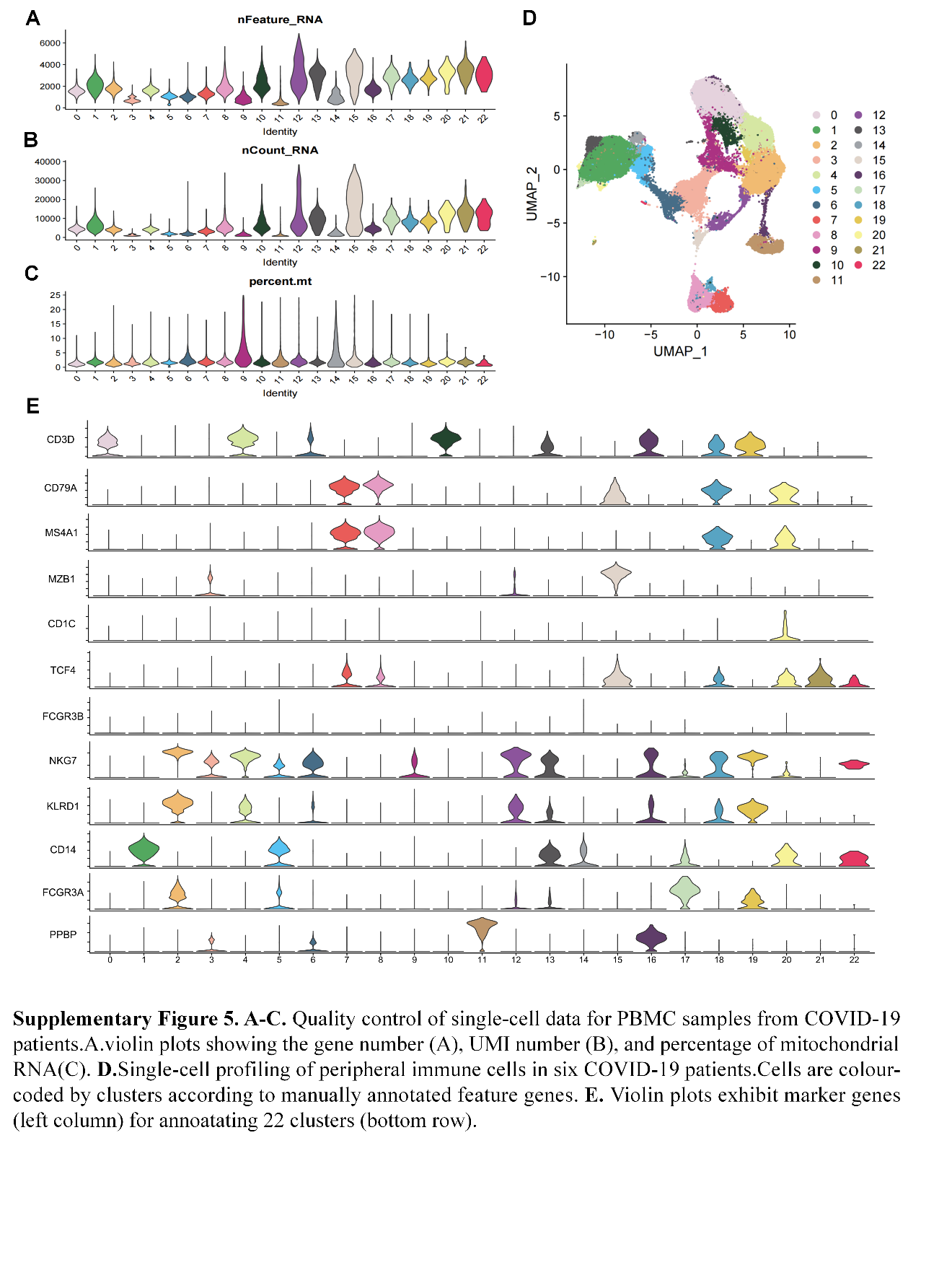
**Supplementary Figure 4.** A-C. Quality control of single-cell data for PBMC samples from COVID-19 patients. Violin plots showing the (A) gene number, (B) UMI number, and (C) percentage of mitochondrial RNA. D. Single-cell profiling of peripheral immune cells in six COVID-19 patients. Cells are colour-coded by clusters according to manually annotated feature genes. E. The violin plots exhibiting marker genes (left column) for annotating 22 clusters (bottom row).


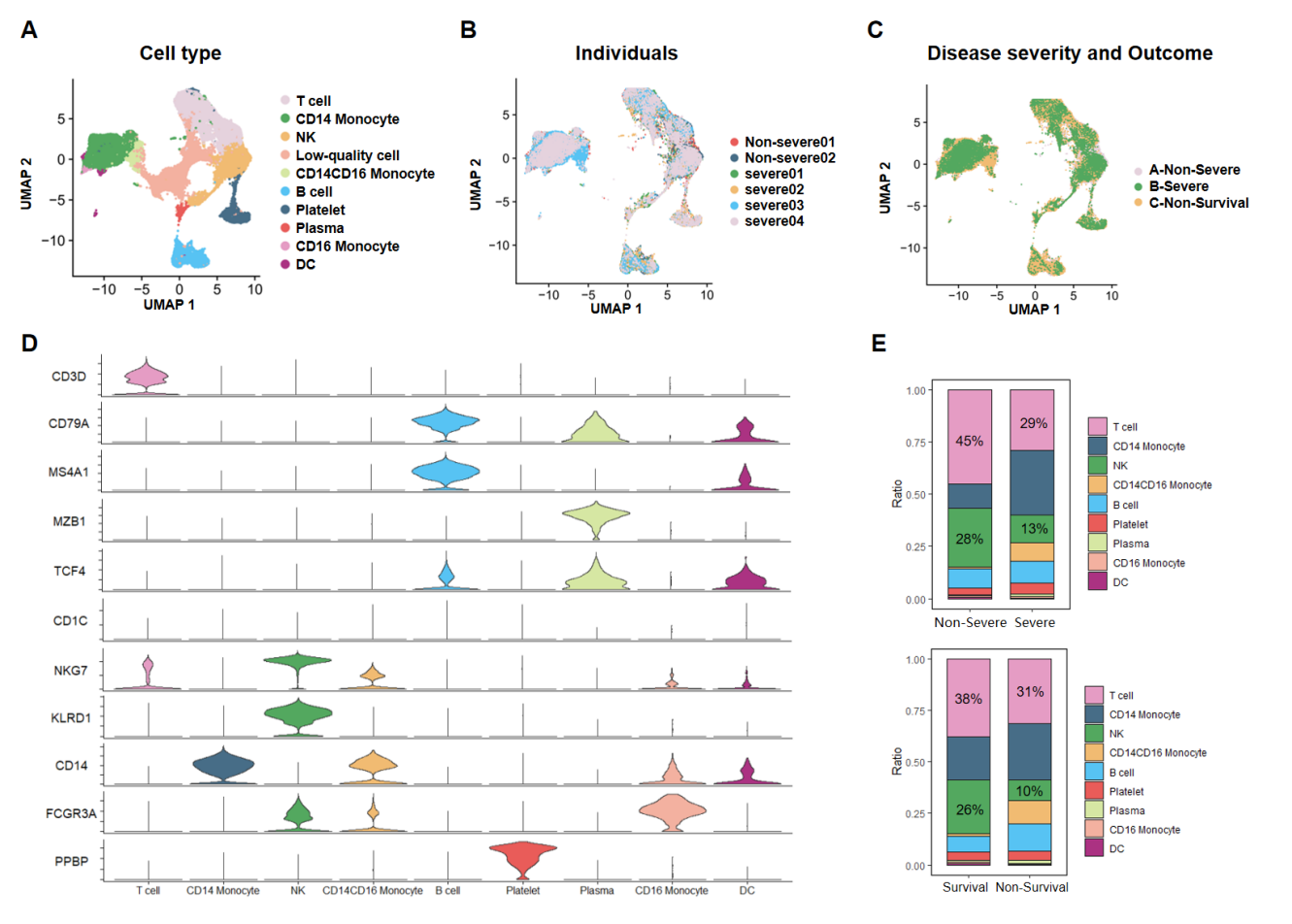
**Supplementary Figure 5**. A-C. The two-dimensional UMAP distribution for a total of 56,954 PBMCs from COVID-19 patients, which were divided into nine subpopulations including T cell, natural killer cells (NK), CD14CD16 monocyte, B cell, Platelet, Plasma, CD16 monocyte, and dendritic cells (DC), while the 11, 285 low-quality cells were excluded. Cells are color-coded by (A) cell types, (B) individuals, and (C) disease severity and outcomes. D. Violin plots exhibiting marker genes (left column) for identifying corresponding cell types (bottom row). E. Bar graphs showing the relative percentages of the above nine cell subsets in patients divided into different groups of disease severity (non-severe and severe) and outcomes (survival and non-survival).

**
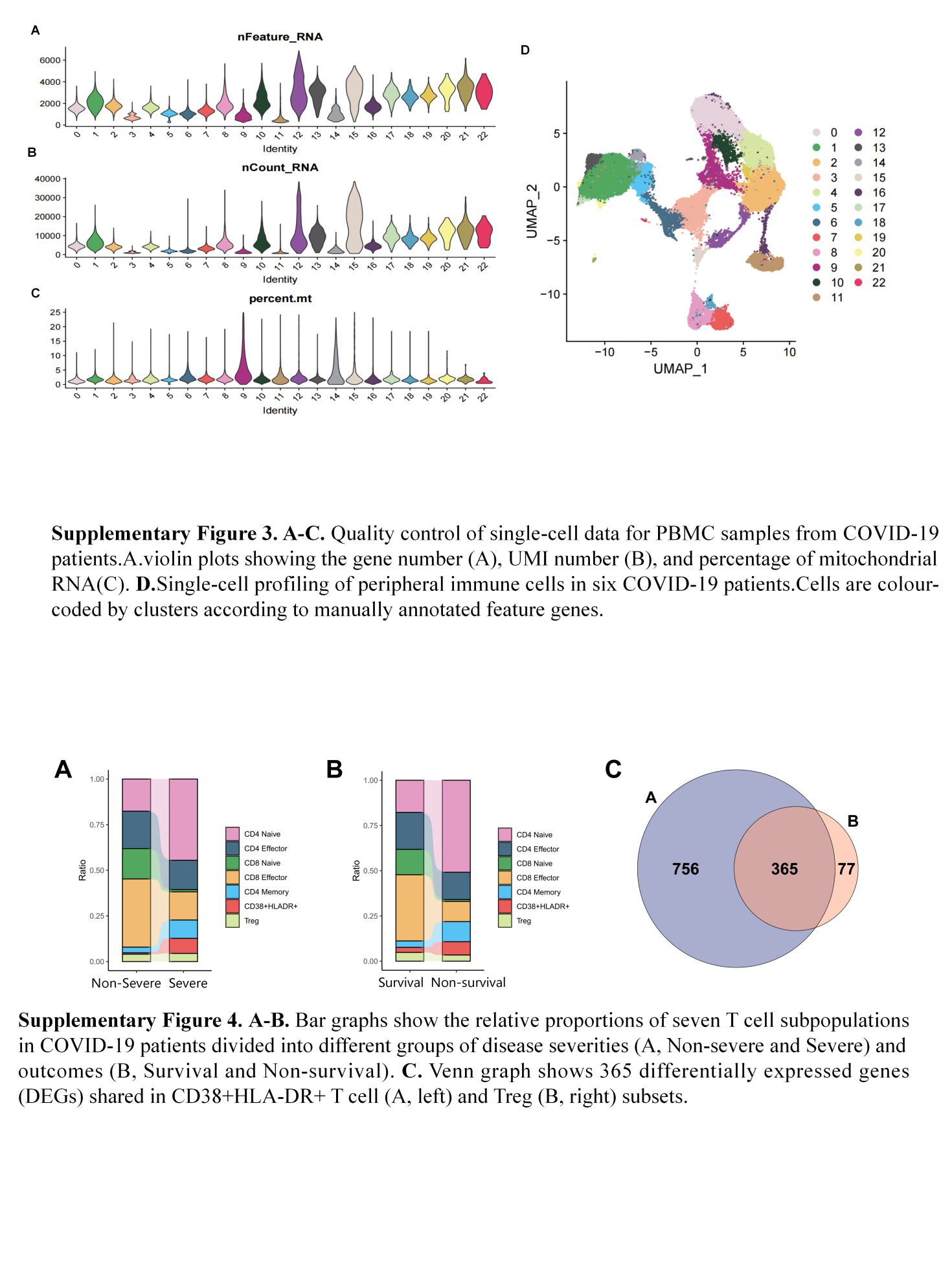
**

**Supplementary Figure 6.** A-B. The relative proportions of seven T cell subpopulations in COVID-19 patients divided into different groups of (A) disease severity and (B) outcomes. C. The venn diagram showing 365 differentially expressed genes (DEGs) shared in CD38^+^HLA-DR^+^ T cells (A, left) and (B, right) Treg subsets.

# Supplementary Tables

| **Supplementary Table 1. Demographics of the cohort.** | | | |
| --- | --- | --- | --- |
| **Characteristics** | **Healthy (n = 10)** | **Bacterial (n = 14)** | **COVID-19 (n = 28)** |
| **Age (years)** |  |  |  |
| Median (IQR) | 24.00 (22.00, 26.00) | 65.00 (60.00, 74.00) | 73.00 (62.00, 82.00) |
| ＞60 years old | 0 (0.00%) | 11 (78.57%) | 23 (82.14%) |
| **Sex, n (%)** |  |  |  |
| Male | 6 (60.00%) | 10 (71.43%) | 21 (75.00%) |
| **Past medical history** |  |  |  |
| Smoking history, n (%) | 1 (10.00%) | 3 (21.43%) | 11 (39.29%) |
| Hypertension, n (%) | 0 (0.00%) | 4 (28.57%) | 11 (39.29%) |
| Diabetes, n (%) | 0 (0.00%) | 2 (14.29%) | 7 (25.00%) |
| Malignant neoplasm, n (%) | 0 (0.0%) | 1 (7.14%) | 2 (7.14%) |
| Metabolic arthritis, n (%) | 0 (0.00%) | 0 (0.00%) | 2 (7.14%) |
| Chronic cardiac disease, n (%) | 0 (0.00%) | 0 (0.00%) | 3 (10.71%) |
| Chronic pulmonary disease, n (%) | 0 (0.00%) | 1 (7.14%) | 9 (32.14%) |
| Chronic liver disease, n (%) | 0 (0.00%) | 0 (0.00%) | 3 (10.71%) |
| Chronic kidney disease, n (%) | 0 (0.00%) | 0 (0.00%) | 2 (7.14%) |
| **Outcomes** |  |  |  |
| Non-suvivial, n (%) | 0 (0.00%) | 0 (0.00%) | 9 (32.14%) |

Data were presented as Median (IQR), n (%) unless otherwise stated. IQR represented the range between the 1st and 3rd quartiles.

| **Supplementary Table 2. The antibodies, fluorochrome conjugates and clones used for flow cytometry analysis.** | | |
| --- | --- | --- |
| **Antibodies** | **Source** | **Identifier** |
| Anti-Human CD3-BV421 | BD | Cat# 562426 |
| Anti-Human CD38-PE | BD | Cat# 555460 |
| Anti-Human CD4-Percp/cy5.5 | BioLegend | Cat# 317428 |
| Anti-Human CD8-APC | BioLegend | Cat# 301014 |
| Anti-Human HLA-DR-APC-H7 | BD | Cat# 561358 |
| Anti-Human aCD56-BV421 | Biolegend | Cat#318328 |
| Anti-Human CD19-BV421 | BD | Cat#562440 |
| Anti-Human CD20-BV421 | BD | Cat#302330 |
| Anti-Human CD88-PE | BD | Cat#344304 |
| Anti-Human CD33-BB515 | BD | Cat#564588 |
| Anti-Human CD11b-PE-Cy7 | BD | Cat#557743 |
| Anti-Human CD15-BV650 | BD | Cat#564232 |
| Anti-Human CD14-BV510 | BD | Cat#563079 |
| Anti-Human CD16-BV786 | BD | Cat#563690 |
| Anti-Human Fc Receptor Blocking Solution | BioLegend | Cat# 422302 |
| Fixable Viability Stain 700-AF700 | BD | Cat# 564997 |

| **Supplementary Table 3. Correlation analysis of common clinical indicators related to CD38^+^HLA-DR^+^ T cells.** | | | | |
| --- | --- | --- | --- | --- |
| **Parameters** | **Non-Severe（n=11）** | **Severe（n=17）** | **r-value** | **p-value** |
| TP (g/L) | 64.60 (61.95,69.35) | 65.70 (60.50,72.20) | 0.142 | 0.473 |
| ALB (g/L) | 64.60 (61.95,69.35) | 65.70 (60.50,72.20) | 0.102 | 0.607 |
| GLO (g/L) | 29.70 (27.65,32.95) | 30.60 (25.70,35.10) | 0.013 | 0.948 |
| TBIL (μmol/L) | 11.40 (8.85,14.10) | 9.60 (8.30,14.30) | 0.108 | 0.584 |
| DBIL (μmol/L) | 7.50 (4.50,9.70) | 4.70 (4.10,10.40) | 0.122 | 0.538 |
| ALT (U/L) | 26.50 (12.60,60.30) | 27.00 (13.30,32.60) | 0.050 | 0.802 |
| AST (U/L) | 30.90 (20.00,90.10) | 30.90 (23.70,55.30) | 0.205 | 0.295 |
| γ-GT (U/L) | 41.90 (28.40,150.85) | 28.70 (20.80,48.10) | -0.030 | 0.880 |
| ALP (U/L) | 73.40 (59.30,117.10) | 60.30 (53.60,81.30) | -0.229 | 0.240 |
| TG (mmol/L) | 0.87 (0.77,1.44) | 1.34 (0.90,1.75) | -0.215 | 0.273 |
| TC (mmol/L) | 4.10 (3.65,4.27) | 3.35 (2.87,3.99) | -0.462 | 0.013* |
| HDL (mmol/L) | 1.13 (0.84,1.19) | 1.01 (0.81,1.15) | -0.191 | 0.330 |
| LDL (mmol/L) | 2.38 (2.17,2.62) | 1.79 (1.40,2.51) | -0.432 | 0.022* |
| APOA (g/L) | 1.08 (0.85,1.15) | 1.04 (0.77,1.08) | 0.066 | 0.739 |
| APOB (g/L) | 0.87 (0.83,1.03) | 0.81 (0.56,1.06) | -0.489 | 0.008* |
| CK (U/L) | 99.30 (59.90,169.75) | 125.80 (38.80,212.80) | 0.162 | 0.409 |
| LDH (U/L) | 275.50 (225.90,287.35) | 256.70 (205.50,444.50) | -0.181 | 0.355 |
| WBC (*10^9^/L) | 5.86 (4.04,7.80) | 6.55 (5.47,8.67) | -0.304 | 0.116 |
| NEUT (*10^9^/L) | 5.13 (3.41,6.49) | 5.19 (4.88,8.09) | -0.365 | 0.056 |
| LYM (*10^9^/L) | 0.71 (0.55,0.94) | 0.44 (0.37,0.90) | 0.065 | 0.742 |
| MONO (*10^9^/L) | 0.42 (0.21,0.61) | 0.34 (0.20,0.61) | -0.284 | 0.143 |
| HGB (*10^9^/L) | 129.00 (119.00,153.00) | 125.00 (121.00,134.00) | 0.138 | 0.483 |
| PLT (*10^9^/L) | 161.00 (131.00,292.50) | 187.00 (152.00,246.00) | -0.510 | 0.006* |
| PCT (ng/ml) | 0.09 (0.06,0.17) | 0.57 (0.14,2.35) | 0.060 | 0.773 |
| Myo (ng/ml) | 68.90 (39.67,98.07) | 107.22 (77.52,233.88) | 0.277 | 0.154 |
| CKMB (ng/ml) | 1.34 (0.72,1.94) | 1.53 (1.18,3.06) | 0.073 | 0.714 |
| cTNT (ng/l) | 10.00 (6.06,13.45) | 14.43 (10.00,43.77) | 0.049 | 0.811 |
| NT-proBNP (pg/ml) | 351.95 (105.32,459.37) | 1544.60 (596.84,3285.90) | 0.109 | 0.589 |
| D-Dimer (ug/ml) | 0.67 (0.40,0.99) | 0.96 (0.83,2.35) | 0.021 | 0.914 |
| FDP (ug/ml) | 2.50 (2.50,3.16) | 4.39 (2.70,8.04) | 0.011 | 0.957 |
| IL6 (pg/ml) | 10.86 (1.79,38.12) | 21.40 (4.88,389.68) | 0.258 | 0.185 |
| IL10 (pg/ml) | 1.92 (1.64,2.39) | 4.75 (2.39,9.61) | 0.094 | 0.676 |

Data are presented as Median (IQR). Data were analyzed using Spearman's rho test. IQR represented the range between the 1st and 3rd quartiles.

**Supplementary Table 4. Demographics and clinical characteristics of study populations.**

| **Characteristics** | **Survival**  **(n = 19)** | **Non-Survival**  **(n = 9)** | ***p*-value** |
| --- | --- | --- | --- |
| Age (years) |  |  |  |
| Median (IQR) | 84.00  (70.00, 86.00) | 71.00  (62.00, 78.00) | 0.121 |
| Age group, n (%) |  |  | 1.000 |
| ＞60 years old | 15 (78.95%) | 8 (88.89%) |  |
| Sex, n (%) |  |  | 0.062 |
| Male | 12 (63.16%) | 9 (100.00%) |  |
| Past medical history |  |  |  |
| Smoking history, n (%) | 6 (31.58%) | 5 (55.56%) | 0.409 |
| Hypertension, n (%) | 8 (42.11%) | 3 (33.33%) | 1.000 |
| Diabetes, n (%) | 3 (33.33%) | 4 (21.05%) | 0.646 |
| Malignant neoplasm, n (%) | 1 (11.11%) | 1 (5.26%) | 1.000 |
| Metabolic arthritis, n (%) | 0 (0.00%) | 2 (10.53%) | 1.000 |
| Chronic cardiac disease, n (%) | 1 (11.11%) | 2 (10.53%) | 1.000 |
| Chronic pulmonary disease, n (%) | 4 (44.44%) | 5 (26.32%) | 0.407 |
| Chronic liver disease, n (%) | 0 (0.00%) | 3 (15.79%) | 0.530 |
| Chronic kidney disease, n (%) | 0 (0.00%) | 2 (10.53%) | 1.000 |

Data are presented as Median (IQR), n (%) unless otherwise stated. IQR represented the range between the 1st and 3rd quartiles.

| **Supplementary Table 5. Clinical information for patients with single-cell sequencing.** | | |
| --- | --- | --- |
| **Characteristics** | **Non-Survival (n = 3)** | **Survival (n = 3)** |
| **Age group, n (%)** |  |  |
| 60-75 years | 1 (33.33%) | 0(0.00%) |
| ＞76 years old | 2 (66.67%) | 3(100.00%) |
| **Sex, n (%)** |  |  |
| Male | 3 (100.00%) | 1 (33.33%) |
| **Past medical history** |  |  |
| Smoking history, n (%) | 2 (66.67%) | 0 (0.00%) |
| Hypertension, n (%) | 2 (66.67%) | 1 (33.33%) |
| Diabetes, n (%) | 1 (33.33%) | 1 (33.33%) |
| Malignant neoplasm, n (%) | 0 (0.00%) | 0 (0.00%) |
| Metabolic arthritis, n (%) | 0 (0.00%) | 0 (0.00%) |
| Chronic cardiac disease, n (%) | 0 (0.00%) | 0 (0.00%) |
| Chronic pulmonary disease, n (%) | 0 (0.00%) | 0 (0.00%) |
| Chronic liver disease, n (%) | 0 (0.00%) | 0 (0.00%) |
| Chronic kidney disease, n (%) | 0 (0.00%) | 0 (0.00%) |

| **Supplementary Table 6. DEGs upregulated in the CD38^+^HLA-DR^+^ T cell.** | | | | | | | | |
| --- | --- | --- | --- | --- | --- | --- | --- | --- |
|  | p_val | | avg_log2FC | pct.1 | pct.2 | p_val_adj | cluster | gene |
| PFN1 | 0 | | 1.932125048 | 1 | 0.931 | 0 | CD38+HLADR+ | PFN1 |
| ACTB | 0 | | 1.814543545 | 1 | 0.993 | 0 | CD38+HLADR+ | ACTB |
| ARPC1B | 0 | | 1.75101855 | 0.965 | 0.465 | 0 | CD38+HLADR+ | ARPC1B |
| COTL1 | 0 | | 1.682884143 | 0.961 | 0.471 | 0 | CD38+HLADR+ | COTL1 |
| IL32 | 8.75E-257 | | 1.66575864 | 0.993 | 0.838 | 2.13E-252 | CD38+HLADR+ | IL32 |
| ACTG1 | 1.57E-250 | | 1.651844935 | 0.992 | 0.944 | 3.82E-246 | CD38+HLADR+ | ACTG1 |
| LGALS11 | 2.47E-124 | | 1.549746328 | 0.697 | 0.308 | 6.02E-120 | CD38+HLADR+ | LGALS1 |
| CNN2 | 2.16E-285 | | 1.538176023 | 0.967 | 0.585 | 5.25E-281 | CD38+HLADR+ | CNN2 |
| CORO1A | 1.85E-300 | | 1.500314488 | 0.995 | 0.753 | 4.51E-296 | CD38+HLADR+ | CORO1A |
| PPP1CA | 0 | | 1.492322621 | 0.909 | 0.31 | 0 | CD38+HLADR+ | PPP1CA |
| S100A4 | 3.34E-237 | | 1.458116256 | 0.992 | 0.845 | 8.14E-233 | CD38+HLADR+ | S100A4 |
| GAPDH1 | 1.47E-276 | 1.434205356 | | 0.997 | 0.948 | 3.57E-272 | CD38+HLADR+ | GAPDH |
| CD52 | 1.79E-303 | 1.419154112 | | 0.995 | 0.81 | 4.36E-299 | CD38+HLADR+ | CD52 |
| CORO1B | 0 | 1.365844661 | | 0.86 | 0.226 | 0 | CD38+HLADR+ | CORO1B |
| UCP2 | 4.97E-270 | 1.36174926 | | 0.902 | 0.335 | 1.21E-265 | CD38+HLADR+ | UCP2 |
| RAC2 | 8.52E-270 | 1.346359074 | | 0.983 | 0.655 | 2.07E-265 | CD38+HLADR+ | RAC2 |
| MYL61 | 6.39E-269 | 1.316488136 | | 0.992 | 0.807 | 1.56E-264 | CD38+HLADR+ | MYL6 |
| CFL11 | 1.84E-279 | 1.25641156 | | 0.999 | 0.933 | 4.47E-275 | CD38+HLADR+ | CFL1 |
| CD27 | 0 | 1.247076829 | | 0.785 | 0.201 | 0 | CD38+HLADR+ | CD27 |
| MT-CO11 | 1.78E-187 | 1.246138306 | | 0.996 | 0.981 | 4.33E-183 | CD38+HLADR+ | MT-CO1 |
| NOSIP1 | 7.83E-151 | 1.244114952 | | 0.868 | 0.514 | 1.91E-146 | CD38+HLADR+ | NOSIP |
| ANXA21 | 2.71E-275 | 1.221631812 | | 0.774 | 0.211 | 6.59E-271 | CD38+HLADR+ | ANXA2 |
| CRIP1 | 5.02E-154 | 1.192558909 | | 0.98 | 0.752 | 1.22E-149 | CD38+HLADR+ | CRIP1 |
| ISG15 | 1.87E-201 | 1.17221281 | | 0.759 | 0.229 | 4.55E-197 | CD38+HLADR+ | ISG15 |
| SLC25A5 | 1.42E-183 | 1.150490093 | | 0.913 | 0.594 | 3.45E-179 | CD38+HLADR+ | SLC25A5 |
| FKBP1A | 0 | 1.122073569 | | 0.828 | 0.216 | 0 | CD38+HLADR+ | FKBP1A |
| CLIC11 | 3.89E-191 | 1.116072076 | | 0.939 | 0.508 | 9.46E-187 | CD38+HLADR+ | CLIC1 |
| TMSB4X1 | 0 | 1.108963206 | | 0.997 | 0.998 | 0 | CD38+HLADR+ | TMSB4X |
| CAPZB | 1.46E-226 | 1.108387065 | | 0.961 | 0.603 | 3.57E-222 | CD38+HLADR+ | CAPZB |
| MIR4435-2HG | 0 | 1.106179361 | | 0.676 | 0.131 | 0 | CD38+HLADR+ | MIR4435-2HG |
| SELL1 | 1.23E-181 | 1.088727556 | | 0.806 | 0.301 | 2.98E-177 | CD38+HLADR+ | SELL |
| DUSP4 | 0 | 1.083328823 | | 0.499 | 0.06 | 0 | CD38+HLADR+ | DUSP4 |
| ARPC21 | 8.63E-199 | 1.081518193 | | 0.975 | 0.723 | 2.10E-194 | CD38+HLADR+ | ARPC2 |
| ITGB12 | 2.85E-159 | 1.068404574 | | 0.79 | 0.325 | 6.95E-155 | CD38+HLADR+ | ITGB1 |
| COX5A | 1.71E-225 | 1.067157343 | | 0.889 | 0.368 | 4.17E-221 | CD38+HLADR+ | COX5A |
| TMSB10 | 1.38E-288 | 1.046364339 | | 1 | 1 | 3.37E-284 | CD38+HLADR+ | TMSB10 |
| S100A102 | 6.73E-159 | 1.035180591 | | 0.989 | 0.761 | 1.64E-154 | CD38+HLADR+ | S100A10 |
| ARPC3 | 1.50E-219 | 1.032999789 | | 0.981 | 0.758 | 3.65E-215 | CD38+HLADR+ | ARPC3 |
| PPIA | 1.05E-230 | 1.018286445 | | 0.997 | 0.944 | 2.56E-226 | CD38+HLADR+ | PPIA |
| ARPC5 | 1.55E-250 | 1.01280405 | | 0.789 | 0.228 | 3.77E-246 | CD38+HLADR+ | ARPC5 |
| TOX2 | 0 | 1.002319503 | | 0.472 | 0.018 | 0 | CD38+HLADR+ | TOX2 |
| LSP11 | 3.44E-170 | 0.999652737 | | 0.96 | 0.606 | 8.37E-166 | CD38+HLADR+ | LSP1 |
| TRAPPC1 | 8.24E-219 | 0.998686612 | | 0.836 | 0.297 | 2.01E-214 | CD38+HLADR+ | TRAPPC1 |
| NPDC1 | 0 | 0.97994566 | | 0.567 | 0.095 | 0 | CD38+HLADR+ | NPDC1 |
| MT1E | 0 | 0.964528098 | | 0.595 | 0.075 | 0 | CD38+HLADR+ | MT1E |
| SH3BGRL31 | 2.11E-196 | 0.959839878 | | 0.999 | 0.92 | 5.15E-192 | CD38+HLADR+ | SH3BGRL3 |
| ARPC4 | 1.23E-182 | 0.952836412 | | 0.888 | 0.395 | 2.99E-178 | CD38+HLADR+ | ARPC4 |
| ARHGDIB | 2.00E-218 | 0.952477535 | | 0.999 | 0.935 | 4.86E-214 | CD38+HLADR+ | ARHGDIB |
| IL2RG1 | 2.76E-157 | 0.948904061 | | 0.936 | 0.514 | 6.71E-153 | CD38+HLADR+ | IL2RG |
| CTLA4 | 0 | 0.9452172 | | 0.528 | 0.068 | 0 | CD38+HLADR+ | CTLA4 |
| ABRACL | 1.99E-208 | 0.930726402 | | 0.829 | 0.284 | 4.86E-204 | CD38+HLADR+ | ABRACL |
| YWHAB | 1.46E-153 | 0.921297719 | | 0.976 | 0.717 | 3.56E-149 | CD38+HLADR+ | YWHAB |
| H3F3A | 1.95E-195 | 0.918349073 | | 0.991 | 0.842 | 4.76E-191 | CD38+HLADR+ | H3F3A |
| LAT | 6.60E-177 | 0.91650898 | | 0.848 | 0.336 | 1.61E-172 | CD38+HLADR+ | LAT |
| ATP5MF | 1.12E-176 | 0.916179442 | | 0.876 | 0.361 | 2.74E-172 | CD38+HLADR+ | ATP5MF |
| CYTOR1 | 4.21E-212 | 0.913249371 | | 0.799 | 0.249 | 1.03E-207 | CD38+HLADR+ | CYTOR |
| ANXA5 | 2.30E-250 | 0.90244389 | | 0.723 | 0.183 | 5.60E-246 | CD38+HLADR+ | ANXA5 |
| WDR1 | 8.51E-189 | 0.899885034 | | 0.757 | 0.255 | 2.07E-184 | CD38+HLADR+ | WDR1 |
| CALM3 | 6.75E-158 | 0.899124324 | | 0.869 | 0.39 | 1.64E-153 | CD38+HLADR+ | CALM3 |
| PSME2 | 6.30E-138 | 0.897675299 | | 0.865 | 0.401 | 1.53E-133 | CD38+HLADR+ | PSME2 |
| MT2A1 | 1.35E-129 | 0.896215832 | | 0.882 | 0.472 | 3.30E-125 | CD38+HLADR+ | MT2A |
| NDUFA12 | 2.29E-166 | 0.892705755 | | 0.805 | 0.317 | 5.57E-162 | CD38+HLADR+ | NDUFA12 |
| GMFG | 3.05E-181 | 0.891408572 | | 0.985 | 0.775 | 7.42E-177 | CD38+HLADR+ | GMFG |
| COX8A | 3.32E-143 | 0.891051073 | | 0.952 | 0.587 | 8.08E-139 | CD38+HLADR+ | COX8A |
| TSPO | 1.10E-154 | 0.885454692 | | 0.896 | 0.428 | 2.67E-150 | CD38+HLADR+ | TSPO |
| ENO1 | 4.61E-148 | 0.884213258 | | 0.9 | 0.439 | 1.12E-143 | CD38+HLADR+ | ENO1 |
| ANXA6 | 2.31E-205 | 0.884099889 | | 0.775 | 0.245 | 5.62E-201 | CD38+HLADR+ | ANXA6 |
| BRK1 | 1.95E-191 | 0.882582799 | | 0.856 | 0.329 | 4.76E-187 | CD38+HLADR+ | BRK1 |
| AP2S1 | 2.23E-302 | 0.875096999 | | 0.709 | 0.15 | 5.42E-298 | CD38+HLADR+ | AP2S1 |
| TALDO1 | 0 | 0.875010871 | | 0.695 | 0.133 | 0 | CD38+HLADR+ | TALDO1 |
| ITGA41 | 1.09E-126 | 0.874840639 | | 0.755 | 0.322 | 2.66E-122 | CD38+HLADR+ | ITGA4 |
| LIMS1 | 3.20E-257 | 0.872933403 | | 0.638 | 0.14 | 7.80E-253 | CD38+HLADR+ | LIMS1 |
| RNF19A | 1.26E-111 | 0.856217621 | | 0.884 | 0.469 | 3.07E-107 | CD38+HLADR+ | RNF19A |
| LIME1 | 3.61E-147 | 0.845848015 | | 0.976 | 0.699 | 8.79E-143 | CD38+HLADR+ | LIME1 |
| TXN | 5.83E-159 | 0.845575449 | | 0.802 | 0.304 | 1.42E-154 | CD38+HLADR+ | TXN |
| CD742 | 2.70E-101 | 0.843105543 | | 0.961 | 0.7 | 6.59E-97 | CD38+HLADR+ | CD74 |
| UBE2L6 | 2.94E-212 | 0.842419476 | | 0.753 | 0.218 | 7.16E-208 | CD38+HLADR+ | UBE2L6 |
| ADSL | 1.86E-212 | 0.841559751 | | 0.709 | 0.199 | 4.54E-208 | CD38+HLADR+ | ADSL |
| AQP32 | 2.22E-164 | 0.831715682 | | 0.675 | 0.214 | 5.41E-160 | CD38+HLADR+ | AQP3 |
| LY6E | 2.98E-117 | 0.826374455 | | 0.965 | 0.629 | 7.26E-113 | CD38+HLADR+ | LY6E |
| IDH2 | 1.62E-184 | 0.821323686 | | 0.733 | 0.227 | 3.94E-180 | CD38+HLADR+ | IDH2 |
| PSMB10 | 2.90E-160 | 0.816039604 | | 0.807 | 0.307 | 7.07E-156 | CD38+HLADR+ | PSMB10 |
| PPP1R181 | 4.78E-210 | 0.81390155 | | 0.714 | 0.196 | 1.16E-205 | CD38+HLADR+ | PPP1R18 |
| C4orf48 | 1.64E-240 | 0.813274353 | | 0.717 | 0.179 | 4.00E-236 | CD38+HLADR+ | C4orf48 |
| EPSTI1 | 0 | 0.811765802 | | 0.579 | 0.095 | 0 | CD38+HLADR+ | EPSTI1 |
| S1PR4 | 4.10E-129 | 0.809713083 | | 0.793 | 0.333 | 9.99E-125 | CD38+HLADR+ | S1PR4 |
| ISG201 | 7.40E-118 | 0.806231348 | | 0.971 | 0.656 | 1.80E-113 | CD38+HLADR+ | ISG20 |
| ATP5F1E | 3.94E-202 | 0.797186744 | | 0.996 | 0.948 | 9.58E-198 | CD38+HLADR+ | ATP5F1E |
| OAZ1 | 4.41E-125 | 0.796873186 | | 0.957 | 0.668 | 1.07E-120 | CD38+HLADR+ | OAZ1 |
| ATP5MC3 | 3.00E-135 | 0.795443569 | | 0.91 | 0.542 | 7.30E-131 | CD38+HLADR+ | ATP5MC3 |
| COPE | 8.63E-143 | 0.791925677 | | 0.866 | 0.389 | 2.10E-138 | CD38+HLADR+ | COPE |
| GNAI2 | 2.11E-128 | 0.787522646 | | 0.934 | 0.597 | 5.14E-124 | CD38+HLADR+ | GNAI2 |
| DBI1 | 1.08E-153 | 0.786593392 | | 0.856 | 0.347 | 2.64E-149 | CD38+HLADR+ | DBI |
| LCK | 2.48E-127 | 0.786314311 | | 0.922 | 0.528 | 6.05E-123 | CD38+HLADR+ | LCK |
| HLA-DRA2 | 7.07E-129 | 0.781477917 | | 0.533 | 0.164 | 1.72E-124 | CD38+HLADR+ | HLA-DRA |
| S100A6 | 5.57E-83 | 0.78144008 | | 0.984 | 0.88 | 1.36E-78 | CD38+HLADR+ | S100A6 |
| GNG5 | 1.74E-148 | 0.773941581 | | 0.85 | 0.353 | 4.23E-144 | CD38+HLADR+ | GNG5 |
| MT-CO32 | 9.19E-60 | 0.769273448 | | 0.985 | 0.951 | 2.24E-55 | CD38+HLADR+ | MT-CO3 |
| H2AFV | 4.55E-124 | 0.768414118 | | 0.862 | 0.417 | 1.11E-119 | CD38+HLADR+ | H2AFV |
| ATP5F1C | 1.55E-130 | 0.767312557 | | 0.837 | 0.385 | 3.78E-126 | CD38+HLADR+ | ATP5F1C |
| ICOS2 | 1.21E-119 | 0.765870019 | | 0.754 | 0.311 | 2.94E-115 | CD38+HLADR+ | ICOS |
| HMGN2 | 6.20E-44 | 0.764433709 | | 0.913 | 0.636 | 1.51E-39 | CD38+HLADR+ | HMGN2 |
| SNX91 | 9.37E-92 | 0.763144098 | | 0.845 | 0.466 | 2.28E-87 | CD38+HLADR+ | SNX9 |
| COX6C | 1.85E-122 | 0.755504222 | | 0.973 | 0.771 | 4.51E-118 | CD38+HLADR+ | COX6C |
| IFI61 | 1.25E-47 | 0.748772654 | | 0.525 | 0.247 | 3.05E-43 | CD38+HLADR+ | IFI6 |
| LGALS9 | 0 | 0.748177651 | | 0.552 | 0.077 | 0 | CD38+HLADR+ | LGALS9 |
| JPT11 | 1.59E-214 | 0.744753756 | | 0.697 | 0.18 | 3.87E-210 | CD38+HLADR+ | JPT1 |
| CUL3 | 6.58E-111 | 0.743720297 | | 0.829 | 0.393 | 1.60E-106 | CD38+HLADR+ | CUL3 |
| RILPL22 | 3.66E-97 | 0.743532731 | | 0.675 | 0.293 | 8.92E-93 | CD38+HLADR+ | RILPL2 |
| SERF2 | 1.58E-167 | 0.742983532 | | 0.997 | 0.939 | 3.84E-163 | CD38+HLADR+ | SERF2 |
| NME2 | 2.93E-104 | 0.739912539 | | 0.939 | 0.72 | 7.14E-100 | CD38+HLADR+ | NME2 |
| CBLB1 | 3.35E-86 | 0.73680605 | | 0.834 | 0.471 | 8.16E-82 | CD38+HLADR+ | CBLB |
| SEPTIN6 | 1.95E-121 | 0.736056585 | | 0.872 | 0.427 | 4.74E-117 | CD38+HLADR+ | SEPTIN6 |
| SEC11A | 1.36E-131 | 0.735790859 | | 0.781 | 0.321 | 3.32E-127 | CD38+HLADR+ | SEC11A |
| PSMB3 | 7.69E-139 | 0.735164412 | | 0.846 | 0.36 | 1.87E-134 | CD38+HLADR+ | PSMB3 |
| S100A111 | 3.09E-103 | 0.732039812 | | 0.763 | 0.325 | 7.53E-99 | CD38+HLADR+ | S100A11 |
| COX6A1 | 1.18E-113 | 0.730519707 | | 0.957 | 0.69 | 2.88E-109 | CD38+HLADR+ | COX6A1 |
| DECR1 | 3.96E-191 | 0.725992766 | | 0.663 | 0.183 | 9.66E-187 | CD38+HLADR+ | DECR1 |
| PGAM1 | 1.25E-113 | 0.723080067 | | 0.829 | 0.387 | 3.05E-109 | CD38+HLADR+ | PGAM1 |
| CSTB | 4.34E-151 | 0.721574994 | | 0.782 | 0.282 | 1.06E-146 | CD38+HLADR+ | CSTB |
| PYCARD | 1.89E-281 | 0.720742578 | | 0.598 | 0.112 | 4.60E-277 | CD38+HLADR+ | PYCARD |
| PLSCR3 | 6.31E-140 | 0.719396819 | | 0.785 | 0.3 | 1.54E-135 | CD38+HLADR+ | PLSCR3 |
| IFITM21 | 8.07E-91 | 0.718210073 | | 0.968 | 0.79 | 1.97E-86 | CD38+HLADR+ | IFITM2 |
| ITGB21 | 1.00E-105 | 0.713247301 | | 0.898 | 0.482 | 2.44E-101 | CD38+HLADR+ | ITGB2 |
| PKM | 1.56E-117 | 0.712002113 | | 0.806 | 0.361 | 3.79E-113 | CD38+HLADR+ | PKM |
| LDLRAD4 | 1.56E-150 | 0.705246209 | | 0.626 | 0.191 | 3.81E-146 | CD38+HLADR+ | LDLRAD4 |
| ARAP21 | 7.49E-130 | 0.70471461 | | 0.735 | 0.274 | 1.82E-125 | CD38+HLADR+ | ARAP2 |
| LAMTOR2 | 2.26E-297 | 0.704066046 | | 0.596 | 0.106 | 5.49E-293 | CD38+HLADR+ | LAMTOR2 |
| NDUFB2 | 3.83E-131 | 0.702386683 | | 0.825 | 0.343 | 9.32E-127 | CD38+HLADR+ | NDUFB2 |
| PSME11 | 1.33E-117 | 0.698570161 | | 0.969 | 0.713 | 3.24E-113 | CD38+HLADR+ | PSME1 |
| MT-CO22 | 8.22E-81 | 0.696784837 | | 0.999 | 0.995 | 2.00E-76 | CD38+HLADR+ | MT-CO2 |
| PPP1CC | 1.57E-116 | 0.695986483 | | 0.866 | 0.413 | 3.83E-112 | CD38+HLADR+ | PPP1CC |
| PSMD8 | 8.26E-152 | 0.693022815 | | 0.785 | 0.278 | 2.01E-147 | CD38+HLADR+ | PSMD8 |
| COX6B1 | 2.27E-120 | 0.690915149 | | 0.961 | 0.677 | 5.54E-116 | CD38+HLADR+ | COX6B1 |
| ZEB1 | 1.64E-91 | 0.690875246 | | 0.741 | 0.344 | 3.99E-87 | CD38+HLADR+ | ZEB1 |
| TKT | 1.62E-134 | 0.690669463 | | 0.773 | 0.3 | 3.95E-130 | CD38+HLADR+ | TKT |
| IFITM11 | 3.30E-74 | 0.687283434 | | 0.989 | 0.97 | 8.04E-70 | CD38+HLADR+ | IFITM1 |
| BLOC1S1 | 2.18E-147 | 0.686067052 | | 0.79 | 0.285 | 5.30E-143 | CD38+HLADR+ | BLOC1S1 |
| AC004585.1 | 0 | 0.685596634 | | 0.337 | 0.011 | 0 | CD38+HLADR+ | AC004585.1 |
| FYB11 | 6.80E-91 | 0.68540204 | | 0.956 | 0.66 | 1.66E-86 | CD38+HLADR+ | FYB1 |
| CSK | 6.78E-149 | 0.683457493 | | 0.771 | 0.272 | 1.65E-144 | CD38+HLADR+ | CSK |
| GTF3C6 | 1.90E-242 | 0.682528375 | | 0.648 | 0.143 | 4.64E-238 | CD38+HLADR+ | GTF3C6 |
| LCP11 | 1.06E-86 | 0.680728827 | | 0.905 | 0.549 | 2.57E-82 | CD38+HLADR+ | LCP1 |
| ITGB7 | 1.21E-200 | 0.676930879 | | 0.658 | 0.169 | 2.94E-196 | CD38+HLADR+ | ITGB7 |
| ZYX | 1.25E-99 | 0.676158673 | | 0.832 | 0.407 | 3.03E-95 | CD38+HLADR+ | ZYX |
| ATP5F1A | 3.00E-100 | 0.676055474 | | 0.842 | 0.453 | 7.30E-96 | CD38+HLADR+ | ATP5F1A |
| C9orf16 | 1.09E-106 | 0.672631227 | | 0.928 | 0.566 | 2.66E-102 | CD38+HLADR+ | C9orf16 |
| PSMB91 | 2.30E-100 | 0.67140653 | | 0.837 | 0.419 | 5.60E-96 | CD38+HLADR+ | PSMB9 |
| SUB1 | 1.69E-109 | 0.671217672 | | 0.983 | 0.781 | 4.11E-105 | CD38+HLADR+ | SUB1 |
| PDCD1 | 2.22E-182 | 0.667349955 | | 0.402 | 0.078 | 5.41E-178 | CD38+HLADR+ | PDCD1 |
| CAP1 | 1.02E-108 | 0.666740651 | | 0.809 | 0.36 | 2.48E-104 | CD38+HLADR+ | CAP1 |
| ETHE1 | 2.43E-236 | 0.665736121 | | 0.615 | 0.133 | 5.91E-232 | CD38+HLADR+ | ETHE1 |
| ATP5PB | 4.62E-100 | 0.665145482 | | 0.832 | 0.426 | 1.12E-95 | CD38+HLADR+ | ATP5PB |
| TWF2 | 7.88E-169 | 0.663804228 | | 0.638 | 0.183 | 1.92E-164 | CD38+HLADR+ | TWF2 |
| AKAP131 | 9.18E-86 | 0.663504549 | | 0.928 | 0.584 | 2.23E-81 | CD38+HLADR+ | AKAP13 |
| TPM4 | 1.36E-131 | 0.661615498 | | 0.75 | 0.282 | 3.31E-127 | CD38+HLADR+ | TPM4 |
| ATP5F1B | 1.18E-89 | 0.661400657 | | 0.909 | 0.6 | 2.87E-85 | CD38+HLADR+ | ATP5F1B |
| STAT1 | 1.93E-146 | 0.657689548 | | 0.579 | 0.168 | 4.71E-142 | CD38+HLADR+ | STAT1 |
| PARK7 | 4.40E-100 | 0.657299553 | | 0.884 | 0.47 | 1.07E-95 | CD38+HLADR+ | PARK7 |
| CARD16 | 7.21E-150 | 0.654491499 | | 0.722 | 0.236 | 1.76E-145 | CD38+HLADR+ | CARD16 |
| MXD41 | 4.03E-155 | 0.65390697 | | 0.68 | 0.211 | 9.81E-151 | CD38+HLADR+ | MXD4 |
| ARF5 | 1.58E-107 | 0.650297433 | | 0.888 | 0.446 | 3.85E-103 | CD38+HLADR+ | ARF5 |
| NDUFA13 | 5.59E-106 | 0.64807247 | | 0.945 | 0.566 | 1.36E-101 | CD38+HLADR+ | NDUFA13 |
| CHST111 | 5.11E-107 | 0.646509196 | | 0.721 | 0.287 | 1.24E-102 | CD38+HLADR+ | CHST11 |
| PSMB8 | 1.69E-86 | 0.646017618 | | 0.865 | 0.48 | 4.12E-82 | CD38+HLADR+ | PSMB8 |
| TRIB2 | 3.00E-272 | 0.644716149 | | 0.496 | 0.081 | 7.30E-268 | CD38+HLADR+ | TRIB2 |
| CD3D1 | 1.77E-93 | 0.642422424 | | 0.976 | 0.777 | 4.32E-89 | CD38+HLADR+ | CD3D |
| PPM1G | 4.42E-134 | 0.640326446 | | 0.758 | 0.275 | 1.08E-129 | CD38+HLADR+ | PPM1G |
| PELI11 | 2.37E-84 | 0.638109043 | | 0.822 | 0.398 | 5.77E-80 | CD38+HLADR+ | PELI1 |
| H2AFY | 1.53E-154 | 0.636373728 | | 0.693 | 0.217 | 3.72E-150 | CD38+HLADR+ | H2AFY |
| SIRPG | 6.18E-290 | 0.635463429 | | 0.473 | 0.07 | 1.50E-285 | CD38+HLADR+ | SIRPG |
| SUMO1 | 2.32E-110 | 0.634242294 | | 0.833 | 0.376 | 5.64E-106 | CD38+HLADR+ | SUMO1 |
| DOK21 | 1.20E-114 | 0.631983796 | | 0.674 | 0.24 | 2.93E-110 | CD38+HLADR+ | DOK2 |
| RGS101 | 1.35E-89 | 0.630124461 | | 0.862 | 0.47 | 3.29E-85 | CD38+HLADR+ | RGS10 |
| UQCR10 | 2.67E-92 | 0.627301287 | | 0.881 | 0.472 | 6.50E-88 | CD38+HLADR+ | UQCR10 |
| ATXN1 | 3.42E-99 | 0.625347175 | | 0.79 | 0.35 | 8.34E-95 | CD38+HLADR+ | ATXN1 |
| TAGLN2 | 1.13E-80 | 0.624237592 | | 0.957 | 0.698 | 2.74E-76 | CD38+HLADR+ | TAGLN2 |
| VAMP8 | 1.77E-108 | 0.623734262 | | 0.845 | 0.374 | 4.32E-104 | CD38+HLADR+ | VAMP8 |
| GBP2 | 1.08E-112 | 0.62215782 | | 0.695 | 0.266 | 2.62E-108 | CD38+HLADR+ | GBP2 |
| PGLS | 5.47E-137 | 0.621729392 | | 0.682 | 0.227 | 1.33E-132 | CD38+HLADR+ | PGLS |
| NDUFB9 | 2.58E-87 | 0.621208595 | | 0.928 | 0.613 | 6.29E-83 | CD38+HLADR+ | NDUFB9 |
| NDUFA11 | 2.90E-103 | 0.619976549 | | 0.91 | 0.483 | 7.06E-99 | CD38+HLADR+ | NDUFA11 |
| HINT1 | 2.80E-102 | 0.619960443 | | 0.987 | 0.889 | 6.82E-98 | CD38+HLADR+ | HINT1 |
| TPI11 | 1.81E-89 | 0.617352613 | | 0.848 | 0.424 | 4.42E-85 | CD38+HLADR+ | TPI1 |
| CYBA1 | 2.04E-94 | 0.616922313 | | 0.987 | 0.829 | 4.96E-90 | CD38+HLADR+ | CYBA |
| NDUFC2 | 3.15E-102 | 0.615924262 | | 0.799 | 0.354 | 7.66E-98 | CD38+HLADR+ | NDUFC2 |
| GBP51 | 1.02E-101 | 0.614584234 | | 0.579 | 0.21 | 2.48E-97 | CD38+HLADR+ | GBP5 |
| MVP | 7.07E-185 | 0.613402672 | | 0.675 | 0.182 | 1.72E-180 | CD38+HLADR+ | MVP |
| GPRIN31 | 3.64E-85 | 0.613270712 | | 0.821 | 0.4 | 8.85E-81 | CD38+HLADR+ | GPRIN3 |
| HMGA1 | 1.59E-125 | 0.610769311 | | 0.63 | 0.213 | 3.87E-121 | CD38+HLADR+ | HMGA1 |
| MICOS10 | 2.44E-96 | 0.607837698 | | 0.778 | 0.351 | 5.94E-92 | CD38+HLADR+ | MICOS10 |
| ETV6 | 3.26E-127 | 0.607253833 | | 0.668 | 0.229 | 7.93E-123 | CD38+HLADR+ | ETV6 |
| RHOG | 7.50E-92 | 0.606890592 | | 0.894 | 0.502 | 1.83E-87 | CD38+HLADR+ | RHOG |
| CLDND11 | 1.42E-76 | 0.603801919 | | 0.68 | 0.311 | 3.46E-72 | CD38+HLADR+ | CLDND1 |
| CCDC167 | 4.69E-190 | 0.603750023 | | 0.547 | 0.127 | 1.14E-185 | CD38+HLADR+ | CCDC167 |
| CTSB | 8.75E-214 | 0.602517979 | | 0.533 | 0.112 | 2.13E-209 | CD38+HLADR+ | CTSB |
| SELENOH | 3.40E-122 | 0.600862945 | | 0.803 | 0.318 | 8.28E-118 | CD38+HLADR+ | SELENOH |
| PRDX11 | 2.26E-107 | 0.600753992 | | 0.778 | 0.328 | 5.52E-103 | CD38+HLADR+ | PRDX1 |
| PARP1 | 4.13E-197 | 0.599343852 | | 0.545 | 0.123 | 1.01E-192 | CD38+HLADR+ | PARP1 |
| ZNHIT1 | 1.84E-168 | 0.598963001 | | 0.65 | 0.18 | 4.48E-164 | CD38+HLADR+ | ZNHIT1 |
| MCUB1 | 2.85E-87 | 0.597664959 | | 0.885 | 0.498 | 6.95E-83 | CD38+HLADR+ | MCUB |
| SEM1 | 3.59E-97 | 0.593214251 | | 0.836 | 0.393 | 8.75E-93 | CD38+HLADR+ | SEM1 |
| PCLAF | 0 | 0.590662353 | | 0.303 | 0.006 | 0 | CD38+HLADR+ | PCLAF |
| LIMD21 | 2.76E-79 | 0.588006419 | | 0.971 | 0.837 | 6.72E-75 | CD38+HLADR+ | LIMD2 |
| FLNA1 | 1.23E-76 | 0.58674029 | | 0.797 | 0.393 | 2.99E-72 | CD38+HLADR+ | FLNA |
| MYH91 | 9.30E-68 | 0.583901486 | | 0.925 | 0.597 | 2.26E-63 | CD38+HLADR+ | MYH9 |
| PLAC81 | 8.85E-33 | 0.579557677 | | 0.563 | 0.347 | 2.16E-28 | CD38+HLADR+ | PLAC8 |
| PSMB2 | 1.06E-143 | 0.577409208 | | 0.615 | 0.185 | 2.57E-139 | CD38+HLADR+ | PSMB2 |
| VAMP5 | 2.09E-115 | 0.577251424 | | 0.672 | 0.239 | 5.10E-111 | CD38+HLADR+ | VAMP5 |
| PSMA4 | 6.93E-135 | 0.576088824 | | 0.636 | 0.201 | 1.69E-130 | CD38+HLADR+ | PSMA4 |
| MYL6B | 0 | 0.575954856 | | 0.409 | 0.024 | 0 | CD38+HLADR+ | MYL6B |
| SLC25A3 | 8.12E-68 | 0.575620801 | | 0.914 | 0.67 | 1.98E-63 | CD38+HLADR+ | SLC25A3 |
| MDH2 | 1.02E-89 | 0.572095901 | | 0.856 | 0.439 | 2.49E-85 | CD38+HLADR+ | MDH2 |
| PHACTR21 | 3.88E-111 | 0.571359551 | | 0.741 | 0.286 | 9.46E-107 | CD38+HLADR+ | PHACTR2 |
| WIPF12 | 3.75E-76 | 0.570105286 | | 0.902 | 0.535 | 9.14E-72 | CD38+HLADR+ | WIPF1 |
| FAM160B1 | 1.11E-147 | 0.567612111 | | 0.586 | 0.166 | 2.70E-143 | CD38+HLADR+ | FAM160B1 |
| TLE5 | 1.74E-93 | 0.567363496 | | 0.995 | 0.93 | 4.23E-89 | CD38+HLADR+ | TLE5 |
| EIF3A | 1.66E-84 | 0.566721433 | | 0.81 | 0.395 | 4.05E-80 | CD38+HLADR+ | EIF3A |
| PTRHD1 | 3.29E-155 | 0.566507696 | | 0.612 | 0.171 | 8.00E-151 | CD38+HLADR+ | PTRHD1 |
| CD991 | 5.40E-67 | 0.564703764 | | 0.964 | 0.759 | 1.31E-62 | CD38+HLADR+ | CD99 |
| NEDD8 | 1.43E-99 | 0.563514329 | | 0.841 | 0.377 | 3.48E-95 | CD38+HLADR+ | NEDD8 |
| CD4 | 2.46E-153 | 0.563510382 | | 0.548 | 0.146 | 5.99E-149 | CD38+HLADR+ | CD4 |
| ITM2A1 | 5.33E-62 | 0.562513523 | | 0.795 | 0.448 | 1.30E-57 | CD38+HLADR+ | ITM2A |
| GPSM3 | 9.13E-74 | 0.562232138 | | 0.956 | 0.727 | 2.22E-69 | CD38+HLADR+ | GPSM3 |
| GNA15 | 2.75E-193 | 0.561114907 | | 0.346 | 0.056 | 6.70E-189 | CD38+HLADR+ | GNA15 |
| ATP5MG | 1.65E-107 | 0.561027559 | | 0.987 | 0.903 | 4.02E-103 | CD38+HLADR+ | ATP5MG |
| MSN1 | 8.54E-80 | 0.560600576 | | 0.81 | 0.402 | 2.08E-75 | CD38+HLADR+ | MSN |
| MT-ATP6 | 2.27E-47 | 0.560005578 | | 0.933 | 0.738 | 5.53E-43 | CD38+HLADR+ | MT-ATP6 |
| MT1F | 6.91E-171 | 0.559511153 | | 0.443 | 0.096 | 1.68E-166 | CD38+HLADR+ | MT1F |
| TMEM156 | 7.08E-161 | 0.556362607 | | 0.547 | 0.139 | 1.72E-156 | CD38+HLADR+ | TMEM156 |
| AP2M1 | 5.39E-125 | 0.553407876 | | 0.717 | 0.249 | 1.31E-120 | CD38+HLADR+ | AP2M1 |
| CD82 | 4.74E-170 | 0.552748689 | | 0.495 | 0.115 | 1.15E-165 | CD38+HLADR+ | CD82 |
| C17orf49 | 1.82E-124 | 0.552217561 | | 0.684 | 0.233 | 4.44E-120 | CD38+HLADR+ | C17orf49 |
| EVL | 7.96E-75 | 0.552167096 | | 0.948 | 0.641 | 1.94E-70 | CD38+HLADR+ | EVL |
| CARHSP1 | 1.36E-227 | 0.550820336 | | 0.521 | 0.1 | 3.32E-223 | CD38+HLADR+ | CARHSP1 |
| MT-ATP82 | 2.52E-38 | 0.549991629 | | 0.993 | 0.965 | 6.14E-34 | CD38+HLADR+ | MT-ATP8 |
| SH3KBP11 | 2.28E-86 | 0.549167923 | | 0.778 | 0.351 | 5.55E-82 | CD38+HLADR+ | SH3KBP1 |
| SHMT2 | 1.83E-201 | 0.549065837 | | 0.504 | 0.104 | 4.45E-197 | CD38+HLADR+ | SHMT2 |
| RNF213 | 2.25E-62 | 0.548473863 | | 0.834 | 0.456 | 5.47E-58 | CD38+HLADR+ | RNF213 |
| PPP4C | 1.55E-106 | 0.548097098 | | 0.766 | 0.305 | 3.78E-102 | CD38+HLADR+ | PPP4C |
| NDUFB3 | 5.95E-170 | 0.547897637 | | 0.564 | 0.141 | 1.45E-165 | CD38+HLADR+ | NDUFB3 |
| ICAM3 | 7.53E-70 | 0.547412718 | | 0.84 | 0.486 | 1.83E-65 | CD38+HLADR+ | ICAM3 |
| HIGD2A | 5.62E-73 | 0.546755129 | | 0.924 | 0.657 | 1.37E-68 | CD38+HLADR+ | HIGD2A |
| TRBC21 | 1.93E-54 | 0.546200381 | | 0.844 | 0.514 | 4.69E-50 | CD38+HLADR+ | TRBC2 |
| NDUFS6 | 1.27E-128 | 0.544276124 | | 0.695 | 0.233 | 3.09E-124 | CD38+HLADR+ | NDUFS6 |
| ADA | 7.05E-245 | 0.543264901 | | 0.491 | 0.085 | 1.72E-240 | CD38+HLADR+ | ADA |
| PSMA5 | 6.14E-105 | 0.542896856 | | 0.687 | 0.259 | 1.50E-100 | CD38+HLADR+ | PSMA5 |
| HMGB11 | 6.22E-73 | 0.540368705 | | 0.981 | 0.853 | 1.52E-68 | CD38+HLADR+ | HMGB1 |
| ARID5B2 | 2.24E-71 | 0.53825712 | | 0.91 | 0.525 | 5.46E-67 | CD38+HLADR+ | ARID5B |
| GET3 | 3.41E-201 | 0.538212772 | | 0.582 | 0.131 | 8.29E-197 | CD38+HLADR+ | GET3 |
| HINT2 | 1.34E-185 | 0.537545454 | | 0.535 | 0.121 | 3.25E-181 | CD38+HLADR+ | HINT2 |
| EIF3I | 4.76E-83 | 0.535344068 | | 0.777 | 0.363 | 1.16E-78 | CD38+HLADR+ | EIF3I |
| ELOB | 1.23E-79 | 0.535007037 | | 0.955 | 0.673 | 2.99E-75 | CD38+HLADR+ | ELOB |
| MPG | 3.68E-175 | 0.534615384 | | 0.62 | 0.16 | 8.96E-171 | CD38+HLADR+ | MPG |
| DDOST | 1.29E-76 | 0.533450413 | | 0.778 | 0.38 | 3.15E-72 | CD38+HLADR+ | DDOST |
| TBCB | 2.96E-119 | 0.533398629 | | 0.69 | 0.239 | 7.22E-115 | CD38+HLADR+ | TBCB |
| PTPRCAP1 | 1.29E-71 | 0.53131285 | | 0.987 | 0.891 | 3.14E-67 | CD38+HLADR+ | PTPRCAP |
| DCTN3 | 7.43E-96 | 0.530999658 | | 0.747 | 0.311 | 1.81E-91 | CD38+HLADR+ | DCTN3 |
| PRELID1 | 1.31E-98 | 0.530485563 | | 0.71 | 0.282 | 3.19E-94 | CD38+HLADR+ | PRELID1 |
| EIF4A1 | 2.14E-64 | 0.527738308 | | 0.948 | 0.749 | 5.21E-60 | CD38+HLADR+ | EIF4A1 |
| NOP10 | 1.57E-105 | 0.527654628 | | 0.655 | 0.237 | 3.82E-101 | CD38+HLADR+ | NOP10 |
| PDCD5 | 8.61E-138 | 0.527605553 | | 0.603 | 0.178 | 2.10E-133 | CD38+HLADR+ | PDCD5 |
| MZT2A | 6.28E-69 | 0.527250264 | | 0.894 | 0.55 | 1.53E-64 | CD38+HLADR+ | MZT2A |
| COX7B | 2.34E-76 | 0.526853161 | | 0.892 | 0.497 | 5.70E-72 | CD38+HLADR+ | COX7B |
| MRPL28 | 1.69E-193 | 0.523749337 | | 0.556 | 0.125 | 4.11E-189 | CD38+HLADR+ | MRPL28 |
| NDUFA7 | 1.40E-118 | 0.523494302 | | 0.701 | 0.247 | 3.42E-114 | CD38+HLADR+ | NDUFA7 |
| MAP2K2 | 1.19E-82 | 0.52259137 | | 0.754 | 0.344 | 2.91E-78 | CD38+HLADR+ | MAP2K2 |
| MAF | 5.90E-116 | 0.522559309 | | 0.441 | 0.123 | 1.44E-111 | CD38+HLADR+ | MAF |
| ACTR3 | 5.31E-75 | 0.521843903 | | 0.735 | 0.34 | 1.29E-70 | CD38+HLADR+ | ACTR3 |
| PSMB61 | 7.30E-82 | 0.521575629 | | 0.766 | 0.343 | 1.78E-77 | CD38+HLADR+ | PSMB6 |
| SMAD3 | 2.50E-160 | 0.521057042 | | 0.52 | 0.128 | 6.08E-156 | CD38+HLADR+ | SMAD3 |
| TOX | 4.70E-88 | 0.519883089 | | 0.287 | 0.074 | 1.14E-83 | CD38+HLADR+ | TOX |
| UBL5 | 2.19E-65 | 0.519552992 | | 0.916 | 0.601 | 5.34E-61 | CD38+HLADR+ | UBL5 |
| SEPTIN1 | 8.20E-77 | 0.519154054 | | 0.858 | 0.438 | 2.00E-72 | CD38+HLADR+ | SEPTIN1 |
| ACTR2 | 1.16E-74 | 0.518949413 | | 0.841 | 0.434 | 2.82E-70 | CD38+HLADR+ | ACTR2 |
| CD59 | 1.33E-146 | 0.518624517 | | 0.374 | 0.079 | 3.24E-142 | CD38+HLADR+ | CD59 |
| ATP5PD | 1.74E-78 | 0.516335963 | | 0.857 | 0.445 | 4.25E-74 | CD38+HLADR+ | ATP5PD |
| LGALS31 | 8.50E-41 | 0.515564859 | | 0.287 | 0.115 | 2.07E-36 | CD38+HLADR+ | LGALS3 |
| ELOVL5 | 6.56E-84 | 0.514821182 | | 0.798 | 0.365 | 1.60E-79 | CD38+HLADR+ | ELOVL5 |
| SHKBP1 | 3.78E-151 | 0.51432233 | | 0.587 | 0.16 | 9.20E-147 | CD38+HLADR+ | SHKBP1 |
| SCP2 | 9.63E-126 | 0.514240151 | | 0.651 | 0.211 | 2.34E-121 | CD38+HLADR+ | SCP2 |
| UQCRH | 8.16E-71 | 0.513831158 | | 0.93 | 0.665 | 1.99E-66 | CD38+HLADR+ | UQCRH |
| MAP3K51 | 3.69E-120 | 0.513581041 | | 0.623 | 0.203 | 8.99E-116 | CD38+HLADR+ | MAP3K5 |
| RGS12 | 3.68E-63 | 0.513392051 | | 0.651 | 0.309 | 8.97E-59 | CD38+HLADR+ | RGS1 |
| TRAPPC5 | 1.55E-104 | 0.512083607 | | 0.722 | 0.273 | 3.77E-100 | CD38+HLADR+ | TRAPPC5 |
| DNAJC15 | 7.72E-111 | 0.511908529 | | 0.725 | 0.266 | 1.88E-106 | CD38+HLADR+ | DNAJC15 |
| UQCRC1 | 3.02E-155 | 0.511695991 | | 0.584 | 0.156 | 7.35E-151 | CD38+HLADR+ | UQCRC1 |
| PRDX2 | 8.70E-91 | 0.510541253 | | 0.75 | 0.311 | 2.12E-86 | CD38+HLADR+ | PRDX2 |
| LPXN | 1.20E-107 | 0.508969263 | | 0.634 | 0.223 | 2.92E-103 | CD38+HLADR+ | LPXN |
| NAMPT1 | 9.76E-83 | 0.508178456 | | 0.606 | 0.235 | 2.38E-78 | CD38+HLADR+ | NAMPT |
| CD281 | 3.66E-102 | 0.508173635 | | 0.754 | 0.297 | 8.91E-98 | CD38+HLADR+ | CD28 |
| POMP1 | 1.95E-68 | 0.507318077 | | 0.848 | 0.479 | 4.76E-64 | CD38+HLADR+ | POMP |
| ATP5MC2 | 2.82E-85 | 0.507219802 | | 0.991 | 0.903 | 6.86E-81 | CD38+HLADR+ | ATP5MC2 |
| THADA | 7.35E-131 | 0.506662184 | | 0.416 | 0.103 | 1.79E-126 | CD38+HLADR+ | THADA |
| TCEA1 | 4.70E-77 | 0.50635394 | | 0.802 | 0.375 | 1.14E-72 | CD38+HLADR+ | TCEA1 |
| WAS | 4.63E-81 | 0.506118106 | | 0.79 | 0.367 | 1.13E-76 | CD38+HLADR+ | WAS |
| PACS1 | 1.71E-78 | 0.505957606 | | 0.71 | 0.306 | 4.16E-74 | CD38+HLADR+ | PACS1 |
| PPP1R7 | 2.91E-131 | 0.505152872 | | 0.643 | 0.199 | 7.09E-127 | CD38+HLADR+ | PPP1R7 |
| TMEM258 | 1.42E-68 | 0.504146114 | | 0.884 | 0.51 | 3.45E-64 | CD38+HLADR+ | TMEM258 |
| OCIAD2 | 6.50E-66 | 0.503805093 | | 0.832 | 0.434 | 1.58E-61 | CD38+HLADR+ | OCIAD2 |
| RAB37 | 3.43E-256 | 0.503649886 | | 0.433 | 0.065 | 8.36E-252 | CD38+HLADR+ | RAB37 |
| RHOA | 1.06E-70 | 0.502062565 | | 0.959 | 0.759 | 2.59E-66 | CD38+HLADR+ | RHOA |
| NUTF2 | 2.17E-120 | 0.501890479 | | 0.651 | 0.215 | 5.29E-116 | CD38+HLADR+ | NUTF2 |
| NDUFB11 | 1.79E-69 | 0.500812718 | | 0.893 | 0.538 | 4.37E-65 | CD38+HLADR+ | NDUFB11 |
| SPTBN1 | 5.66E-128 | 0.499020884 | | 0.54 | 0.158 | 1.38E-123 | CD38+HLADR+ | SPTBN1 |
| DBNL | 6.38E-123 | 0.498670215 | | 0.6 | 0.188 | 1.55E-118 | CD38+HLADR+ | DBNL |
| SH2D1A2 | 5.36E-87 | 0.498525234 | | 0.556 | 0.202 | 1.30E-82 | CD38+HLADR+ | SH2D1A |
| HDAC1 | 5.06E-141 | 0.498086512 | | 0.56 | 0.157 | 1.23E-136 | CD38+HLADR+ | HDAC1 |
| LDHB2 | 3.90E-66 | 0.497876907 | | 0.964 | 0.807 | 9.50E-62 | CD38+HLADR+ | LDHB |
| PHTF2 | 1.90E-112 | 0.497726363 | | 0.54 | 0.169 | 4.62E-108 | CD38+HLADR+ | PHTF2 |
| JARID2 | 1.77E-118 | 0.496944271 | | 0.524 | 0.157 | 4.31E-114 | CD38+HLADR+ | JARID2 |
| LRRC8C1 | 7.50E-77 | 0.496841048 | | 0.738 | 0.336 | 1.83E-72 | CD38+HLADR+ | LRRC8C |
| IGBP1 | 7.35E-78 | 0.4966936 | | 0.803 | 0.376 | 1.79E-73 | CD38+HLADR+ | IGBP1 |
| TPM3 | 5.61E-60 | 0.495305001 | | 0.968 | 0.763 | 1.37E-55 | CD38+HLADR+ | TPM3 |
| CACYBP | 2.19E-91 | 0.495151739 | | 0.655 | 0.253 | 5.34E-87 | CD38+HLADR+ | CACYBP |
| TMEM256 | 3.63E-101 | 0.494480461 | | 0.675 | 0.252 | 8.84E-97 | CD38+HLADR+ | TMEM256 |
| TLK1 | 3.92E-81 | 0.494477293 | | 0.698 | 0.3 | 9.54E-77 | CD38+HLADR+ | TLK1 |
| SASH31 | 1.15E-130 | 0.49433498 | | 0.56 | 0.164 | 2.81E-126 | CD38+HLADR+ | SASH3 |
| STMN1 | 2.60E-91 | 0.494023093 | | 0.324 | 0.087 | 6.34E-87 | CD38+HLADR+ | STMN1 |
| BCL2L11 | 2.77E-129 | 0.491248123 | | 0.443 | 0.114 | 6.74E-125 | CD38+HLADR+ | BCL2L11 |
| VSIR1 | 7.22E-69 | 0.491240582 | | 0.782 | 0.393 | 1.76E-64 | CD38+HLADR+ | VSIR |
| TAP1 | 1.26E-79 | 0.490316287 | | 0.67 | 0.283 | 3.06E-75 | CD38+HLADR+ | TAP1 |
| FAM49B1 | 3.27E-76 | 0.490254099 | | 0.773 | 0.355 | 7.97E-72 | CD38+HLADR+ | FAM49B |
| PRDX5 | 2.24E-90 | 0.490023076 | | 0.729 | 0.299 | 5.45E-86 | CD38+HLADR+ | PRDX5 |
| ATP5IF1 | 3.87E-65 | 0.4891449 | | 0.88 | 0.526 | 9.43E-61 | CD38+HLADR+ | ATP5IF1 |
| CD53 | 5.90E-59 | 0.489076343 | | 0.873 | 0.52 | 1.44E-54 | CD38+HLADR+ | CD53 |
| PPP2R1A | 2.20E-83 | 0.488329621 | | 0.757 | 0.328 | 5.35E-79 | CD38+HLADR+ | PPP2R1A |
| TIGIT | 1.20E-90 | 0.487654414 | | 0.313 | 0.083 | 2.91E-86 | CD38+HLADR+ | TIGIT |
| VDAC1 | 2.77E-97 | 0.486389153 | | 0.666 | 0.252 | 6.75E-93 | CD38+HLADR+ | VDAC1 |
| SEPTIN9 | 1.29E-60 | 0.485850403 | | 0.9 | 0.55 | 3.15E-56 | CD38+HLADR+ | SEPTIN9 |
| PRDX3 | 2.33E-204 | 0.485186977 | | 0.436 | 0.079 | 5.67E-200 | CD38+HLADR+ | PRDX3 |
| NDUFA1 | 9.55E-65 | 0.484865022 | | 0.921 | 0.584 | 2.33E-60 | CD38+HLADR+ | NDUFA1 |
| PHPT1 | 9.86E-125 | 0.484835823 | | 0.511 | 0.145 | 2.40E-120 | CD38+HLADR+ | PHPT1 |
| CMTM7 | 1.29E-96 | 0.484150834 | | 0.631 | 0.23 | 3.15E-92 | CD38+HLADR+ | CMTM7 |
| BICDL11 | 1.19E-172 | 0.484034624 | | 0.515 | 0.119 | 2.91E-168 | CD38+HLADR+ | BICDL1 |
| SSBP41 | 1.21E-86 | 0.483794104 | | 0.726 | 0.304 | 2.96E-82 | CD38+HLADR+ | SSBP4 |
| CD38 | 9.46E-288 | 0.483550495 | | 0.283 | 0.025 | 2.30E-283 | CD38+HLADR+ | CD38 |
| URM1 | 2.15E-132 | 0.482707259 | | 0.654 | 0.202 | 5.23E-128 | CD38+HLADR+ | URM1 |
| SETD2 | 1.22E-78 | 0.482568565 | | 0.737 | 0.322 | 2.97E-74 | CD38+HLADR+ | SETD2 |
| CAPN1 | 1.31E-227 | 0.48224148 | | 0.476 | 0.084 | 3.19E-223 | CD38+HLADR+ | CAPN1 |
| OSBPL8 | 2.35E-57 | 0.482197706 | | 0.83 | 0.465 | 5.72E-53 | CD38+HLADR+ | OSBPL8 |
| PFKL | 1.01E-163 | 0.481791837 | | 0.492 | 0.115 | 2.47E-159 | CD38+HLADR+ | PFKL |
| FERMT3 | 5.50E-121 | 0.479763305 | | 0.574 | 0.178 | 1.34E-116 | CD38+HLADR+ | FERMT3 |
| MYO1G1 | 4.00E-91 | 0.479367225 | | 0.68 | 0.265 | 9.75E-87 | CD38+HLADR+ | MYO1G |
| POLD4 | 1.49E-86 | 0.47913184 | | 0.719 | 0.3 | 3.63E-82 | CD38+HLADR+ | POLD4 |
| UBE2V1 | 9.45E-79 | 0.479010814 | | 0.767 | 0.353 | 2.30E-74 | CD38+HLADR+ | UBE2V1 |
| CKLF | 1.76E-100 | 0.47870801 | | 0.596 | 0.206 | 4.29E-96 | CD38+HLADR+ | CKLF |
| BIN1 | 2.43E-63 | 0.478379176 | | 0.821 | 0.442 | 5.92E-59 | CD38+HLADR+ | BIN1 |
| RALY | 5.76E-67 | 0.476792378 | | 0.876 | 0.479 | 1.40E-62 | CD38+HLADR+ | RALY |
| FIBP | 1.01E-181 | 0.476753992 | | 0.505 | 0.111 | 2.45E-177 | CD38+HLADR+ | FIBP |
| HSPA81 | 1.60E-55 | 0.474958349 | | 0.973 | 0.856 | 3.88E-51 | CD38+HLADR+ | HSPA8 |
| STK24 | 2.71E-82 | 0.474876503 | | 0.705 | 0.293 | 6.61E-78 | CD38+HLADR+ | STK24 |
| NDUFA2 | 5.88E-75 | 0.474010942 | | 0.726 | 0.323 | 1.43E-70 | CD38+HLADR+ | NDUFA2 |
| NDUFB8 | 1.46E-53 | 0.473868181 | | 0.89 | 0.564 | 3.55E-49 | CD38+HLADR+ | NDUFB8 |
| CEMIP21 | 6.55E-56 | 0.473104941 | | 0.864 | 0.51 | 1.60E-51 | CD38+HLADR+ | CEMIP2 |
| NDUFB10 | 1.44E-73 | 0.472828596 | | 0.81 | 0.383 | 3.51E-69 | CD38+HLADR+ | NDUFB10 |
| SIPA1L1 | 1.38E-60 | 0.472624588 | | 0.612 | 0.282 | 3.36E-56 | CD38+HLADR+ | SIPA1L1 |
| ITGAE1 | 2.94E-122 | 0.468134629 | | 0.582 | 0.176 | 7.17E-118 | CD38+HLADR+ | ITGAE |
| PFDN4 | 1.70E-112 | 0.468048088 | | 0.594 | 0.193 | 4.13E-108 | CD38+HLADR+ | PFDN4 |
| ELMO1 | 1.47E-83 | 0.467484009 | | 0.758 | 0.326 | 3.59E-79 | CD38+HLADR+ | ELMO1 |
| ALOX5AP2 | 1.10E-43 | 0.46742448 | | 0.671 | 0.372 | 2.69E-39 | CD38+HLADR+ | ALOX5AP |
| COX5B | 1.15E-57 | 0.467150244 | | 0.948 | 0.697 | 2.80E-53 | CD38+HLADR+ | COX5B |
| IFI27L2 | 6.42E-117 | 0.46706657 | | 0.6 | 0.191 | 1.56E-112 | CD38+HLADR+ | IFI27L2 |
| MRPL102 | 1.86E-67 | 0.466690891 | | 0.731 | 0.343 | 4.53E-63 | CD38+HLADR+ | MRPL10 |
| UQCRC2 | 5.26E-87 | 0.465972314 | | 0.711 | 0.289 | 1.28E-82 | CD38+HLADR+ | UQCRC2 |
| HNRNPA2B1 | 2.31E-51 | 0.465199582 | | 0.976 | 0.792 | 5.61E-47 | CD38+HLADR+ | HNRNPA2B1 |
| APBB1IP | 3.35E-69 | 0.464952598 | | 0.725 | 0.335 | 8.16E-65 | CD38+HLADR+ | APBB1IP |
| CYTH1 | 5.51E-55 | 0.464576367 | | 0.885 | 0.511 | 1.34E-50 | CD38+HLADR+ | CYTH1 |
| OGDH1 | 2.80E-101 | 0.464506914 | | 0.652 | 0.236 | 6.82E-97 | CD38+HLADR+ | OGDH |
| COX7A2 | 5.50E-61 | 0.463349754 | | 0.941 | 0.693 | 1.34E-56 | CD38+HLADR+ | COX7A2 |
| HSPD1 | 7.62E-69 | 0.463241108 | | 0.757 | 0.367 | 1.86E-64 | CD38+HLADR+ | HSPD1 |
| SESN32 | 4.10E-47 | 0.461904819 | | 0.663 | 0.351 | 9.99E-43 | CD38+HLADR+ | SESN3 |
| CD3G1 | 6.64E-59 | 0.461832813 | | 0.898 | 0.556 | 1.62E-54 | CD38+HLADR+ | CD3G |
| ROMO1 | 6.15E-73 | 0.461537099 | | 0.755 | 0.352 | 1.50E-68 | CD38+HLADR+ | ROMO1 |
| SELPLG1 | 3.40E-57 | 0.461482155 | | 0.822 | 0.439 | 8.27E-53 | CD38+HLADR+ | SELPLG |
| UBE2N | 3.79E-75 | 0.461468417 | | 0.775 | 0.353 | 9.23E-71 | CD38+HLADR+ | UBE2N |
| SKAP1 | 5.00E-51 | 0.461032543 | | 0.892 | 0.534 | 1.22E-46 | CD38+HLADR+ | SKAP1 |
| RCSD1 | 2.59E-84 | 0.460854003 | | 0.697 | 0.28 | 6.30E-80 | CD38+HLADR+ | RCSD1 |
| LGALS3BP | 6.14E-182 | 0.460669254 | | 0.37 | 0.065 | 1.49E-177 | CD38+HLADR+ | LGALS3BP |
| ANXA111 | 7.69E-68 | 0.460551629 | | 0.826 | 0.415 | 1.87E-63 | CD38+HLADR+ | ANXA11 |
| ATP6V1F | 2.75E-87 | 0.459383104 | | 0.771 | 0.319 | 6.69E-83 | CD38+HLADR+ | ATP6V1F |
| TP53INP1 | 1.37E-185 | 0.458669426 | | 0.341 | 0.055 | 3.34E-181 | CD38+HLADR+ | TP53INP1 |
| LYPLA1 | 1.81E-167 | 0.458654257 | | 0.533 | 0.127 | 4.40E-163 | CD38+HLADR+ | LYPLA1 |
| NDUFB4 | 1.85E-59 | 0.458455414 | | 0.861 | 0.484 | 4.50E-55 | CD38+HLADR+ | NDUFB4 |
| SFT2D1 | 6.00E-143 | 0.458069014 | | 0.507 | 0.131 | 1.46E-138 | CD38+HLADR+ | SFT2D1 |
| TMBIM6 | 3.08E-48 | 0.457152186 | | 0.858 | 0.537 | 7.49E-44 | CD38+HLADR+ | TMBIM6 |
| PTPRJ1 | 2.66E-70 | 0.456995835 | | 0.592 | 0.245 | 6.47E-66 | CD38+HLADR+ | PTPRJ |
| CTSD1 | 1.08E-85 | 0.456936163 | | 0.713 | 0.289 | 2.63E-81 | CD38+HLADR+ | CTSD |
| RNF167 | 2.08E-120 | 0.456656945 | | 0.6 | 0.186 | 5.06E-116 | CD38+HLADR+ | RNF167 |
| EMB | 9.92E-60 | 0.455978244 | | 0.779 | 0.403 | 2.42E-55 | CD38+HLADR+ | EMB |
| ANAPC5 | 8.08E-110 | 0.455646744 | | 0.61 | 0.203 | 1.97E-105 | CD38+HLADR+ | ANAPC5 |
| CHCHD5 | 1.06E-125 | 0.455571972 | | 0.574 | 0.17 | 2.58E-121 | CD38+HLADR+ | CHCHD5 |
| YWHAQ1 | 1.40E-59 | 0.455189113 | | 0.811 | 0.435 | 3.40E-55 | CD38+HLADR+ | YWHAQ |
| HCLS1 | 9.27E-55 | 0.454309905 | | 0.869 | 0.532 | 2.26E-50 | CD38+HLADR+ | HCLS1 |
| EPB41 | 1.82E-66 | 0.453633775 | | 0.765 | 0.371 | 4.43E-62 | CD38+HLADR+ | EPB41 |
| UQCRFS1 | 6.83E-58 | 0.453346841 | | 0.842 | 0.49 | 1.66E-53 | CD38+HLADR+ | UQCRFS1 |
| PSMB4 | 1.04E-114 | 0.452069334 | | 0.65 | 0.214 | 2.54E-110 | CD38+HLADR+ | PSMB4 |
| DRAP1 | 1.81E-69 | 0.452029061 | | 0.83 | 0.413 | 4.40E-65 | CD38+HLADR+ | DRAP1 |
| MIR181A1HG | 2.21E-251 | 0.451667603 | | 0.27 | 0.026 | 5.39E-247 | CD38+HLADR+ | MIR181A1HG |
| DEF6 | 4.20E-83 | 0.45165777 | | 0.714 | 0.295 | 1.02E-78 | CD38+HLADR+ | DEF6 |
| MFNG | 6.56E-84 | 0.451142412 | | 0.659 | 0.262 | 1.60E-79 | CD38+HLADR+ | MFNG |
| ITGB2-AS1 | 5.64E-123 | 0.451034804 | | 0.5 | 0.141 | 1.37E-118 | CD38+HLADR+ | ITGB2-AS1 |
| LYST1 | 3.69E-69 | 0.450334463 | | 0.603 | 0.249 | 9.00E-65 | CD38+HLADR+ | LYST |
| ATP5PF | 6.59E-69 | 0.450255975 | | 0.789 | 0.379 | 1.61E-64 | CD38+HLADR+ | ATP5PF |
| MT-ND51 | 1.06E-29 | 0.450189379 | | 0.922 | 0.724 | 2.58E-25 | CD38+HLADR+ | MT-ND5 |
| FBXW5 | 1.27E-121 | 0.449528593 | | 0.588 | 0.18 | 3.10E-117 | CD38+HLADR+ | FBXW5 |
| GYPC | 1.24E-37 | 0.448551918 | | 0.906 | 0.714 | 3.03E-33 | CD38+HLADR+ | GYPC |
| PPA1 | 1.01E-72 | 0.448107723 | | 0.666 | 0.293 | 2.45E-68 | CD38+HLADR+ | PPA1 |
| CAPG | 9.97E-140 | 0.447987306 | | 0.274 | 0.048 | 2.43E-135 | CD38+HLADR+ | CAPG |
| DNPH1 | 3.38E-181 | 0.447616157 | | 0.483 | 0.102 | 8.23E-177 | CD38+HLADR+ | DNPH1 |
| GSTK1 | 5.20E-46 | 0.447508317 | | 0.953 | 0.765 | 1.27E-41 | CD38+HLADR+ | GSTK1 |
| ITPA | 1.77E-159 | 0.447204172 | | 0.517 | 0.124 | 4.32E-155 | CD38+HLADR+ | ITPA |
| TSPAN14 | 8.17E-81 | 0.446974464 | | 0.584 | 0.224 | 1.99E-76 | CD38+HLADR+ | TSPAN14 |
| GNB21 | 2.99E-62 | 0.446549051 | | 0.813 | 0.414 | 7.27E-58 | CD38+HLADR+ | GNB2 |
| TXN2 | 1.44E-122 | 0.445819121 | | 0.563 | 0.168 | 3.51E-118 | CD38+HLADR+ | TXN2 |
| PSMA2 | 4.72E-60 | 0.445520579 | | 0.761 | 0.388 | 1.15E-55 | CD38+HLADR+ | PSMA2 |
| KIF5B | 1.74E-63 | 0.445138535 | | 0.734 | 0.349 | 4.25E-59 | CD38+HLADR+ | KIF5B |
| LSM2 | 2.32E-129 | 0.445034227 | | 0.515 | 0.142 | 5.64E-125 | CD38+HLADR+ | LSM2 |
| MRPL54 | 1.06E-68 | 0.444871376 | | 0.798 | 0.384 | 2.57E-64 | CD38+HLADR+ | MRPL54 |
| SSNA1 | 1.46E-117 | 0.443829799 | | 0.67 | 0.22 | 3.55E-113 | CD38+HLADR+ | SSNA1 |
| ABHD17A1 | 5.21E-66 | 0.443645178 | | 0.84 | 0.437 | 1.27E-61 | CD38+HLADR+ | ABHD17A |
| PAK2 | 3.39E-54 | 0.442925622 | | 0.826 | 0.451 | 8.25E-50 | CD38+HLADR+ | PAK2 |
| SEC61B | 5.72E-53 | 0.442452564 | | 0.892 | 0.566 | 1.39E-48 | CD38+HLADR+ | SEC61B |
| HLA-DRB12 | 3.06E-35 | 0.44243605 | | 0.524 | 0.281 | 7.44E-31 | CD38+HLADR+ | HLA-DRB1 |
| PREX1 | 2.40E-78 | 0.441831723 | | 0.718 | 0.303 | 5.85E-74 | CD38+HLADR+ | PREX1 |
| CCT3 | 1.32E-96 | 0.441128216 | | 0.662 | 0.244 | 3.21E-92 | CD38+HLADR+ | CCT3 |
| ETFB | 7.49E-109 | 0.440738562 | | 0.575 | 0.185 | 1.82E-104 | CD38+HLADR+ | ETFB |
| RAPGEF11 | 9.29E-79 | 0.439956588 | | 0.647 | 0.261 | 2.26E-74 | CD38+HLADR+ | RAPGEF1 |
| M6PR | 1.59E-118 | 0.439427718 | | 0.566 | 0.173 | 3.86E-114 | CD38+HLADR+ | M6PR |
| NCKAP1L | 1.46E-126 | 0.439122771 | | 0.525 | 0.148 | 3.56E-122 | CD38+HLADR+ | NCKAP1L |
| SIT1 | 1.20E-88 | 0.438635663 | | 0.568 | 0.204 | 2.93E-84 | CD38+HLADR+ | SIT1 |
| SH3BP1 | 9.82E-130 | 0.437517231 | | 0.503 | 0.137 | 2.39E-125 | CD38+HLADR+ | SH3BP1 |
| OPTN | 2.12E-69 | 0.436654581 | | 0.729 | 0.331 | 5.17E-65 | CD38+HLADR+ | OPTN |
| MRPL51 | 4.17E-136 | 0.436521489 | | 0.495 | 0.129 | 1.02E-131 | CD38+HLADR+ | MRPL51 |
| CTSA1 | 4.09E-117 | 0.436089029 | | 0.48 | 0.135 | 9.96E-113 | CD38+HLADR+ | CTSA |
| HIST1H1C | 6.11E-71 | 0.435981368 | | 0.61 | 0.249 | 1.49E-66 | CD38+HLADR+ | HIST1H1C |
| APOBEC3C | 3.59E-215 | 0.434535733 | | 0.364 | 0.055 | 8.75E-211 | CD38+HLADR+ | APOBEC3C |
| PPM1M | 1.32E-171 | 0.434433621 | | 0.473 | 0.102 | 3.20E-167 | CD38+HLADR+ | PPM1M |
| C1QBP | 4.99E-51 | 0.43388974 | | 0.777 | 0.442 | 1.21E-46 | CD38+HLADR+ | C1QBP |
| UFC1 | 7.93E-52 | 0.430042919 | | 0.845 | 0.506 | 1.93E-47 | CD38+HLADR+ | UFC1 |
| ERGIC3 | 1.01E-75 | 0.428926066 | | 0.69 | 0.291 | 2.46E-71 | CD38+HLADR+ | ERGIC3 |
| NDUFS7 | 9.89E-95 | 0.427748834 | | 0.652 | 0.239 | 2.41E-90 | CD38+HLADR+ | NDUFS7 |
| MRPL52 | 8.74E-109 | 0.427742228 | | 0.606 | 0.2 | 2.13E-104 | CD38+HLADR+ | MRPL52 |
| NDUFB71 | 8.72E-82 | 0.427717098 | | 0.77 | 0.32 | 2.12E-77 | CD38+HLADR+ | NDUFB7 |
| CHMP2A | 3.36E-112 | 0.427274414 | | 0.583 | 0.184 | 8.18E-108 | CD38+HLADR+ | CHMP2A |
| CHURC1 | 1.42E-55 | 0.426049566 | | 0.841 | 0.47 | 3.47E-51 | CD38+HLADR+ | CHURC1 |
| DDT | 4.89E-66 | 0.424586542 | | 0.75 | 0.35 | 1.19E-61 | CD38+HLADR+ | DDT |
| DOCK10 | 2.38E-54 | 0.424510182 | | 0.798 | 0.426 | 5.80E-50 | CD38+HLADR+ | DOCK10 |
| TNFRSF41 | 3.78E-72 | 0.424501366 | | 0.313 | 0.095 | 9.20E-68 | CD38+HLADR+ | TNFRSF4 |
| AURKAIP1 | 1.49E-69 | 0.424092806 | | 0.783 | 0.354 | 3.64E-65 | CD38+HLADR+ | AURKAIP1 |
| BTBD111 | 1.80E-96 | 0.423812039 | | 0.457 | 0.141 | 4.37E-92 | CD38+HLADR+ | BTBD11 |
| ATP6V0B1 | 2.61E-80 | 0.423534944 | | 0.689 | 0.277 | 6.35E-76 | CD38+HLADR+ | ATP6V0B |
| PPP2R5C1 | 9.12E-47 | 0.423369112 | | 0.933 | 0.663 | 2.22E-42 | CD38+HLADR+ | PPP2R5C |
| TRAC | 1.67E-103 | 0.423273606 | | 0.54 | 0.173 | 4.07E-99 | CD38+HLADR+ | TRAC |
| CUTA | 7.89E-49 | 0.422612807 | | 0.909 | 0.571 | 1.92E-44 | CD38+HLADR+ | CUTA |
| ADD3 | 4.67E-51 | 0.422452286 | | 0.779 | 0.417 | 1.14E-46 | CD38+HLADR+ | ADD3 |
| WDR83OS1 | 8.54E-56 | 0.420422779 | | 0.82 | 0.434 | 2.08E-51 | CD38+HLADR+ | WDR83OS |
| CSNK2B | 1.99E-58 | 0.419697861 | | 0.864 | 0.482 | 4.84E-54 | CD38+HLADR+ | CSNK2B |
| EVI2B1 | 1.94E-56 | 0.419524791 | | 0.75 | 0.383 | 4.72E-52 | CD38+HLADR+ | EVI2B |
| GLIPR2 | 5.87E-137 | 0.419150208 | | 0.465 | 0.117 | 1.43E-132 | CD38+HLADR+ | GLIPR2 |
| SLC2A31 | 1.78E-22 | 0.418315398 | | 0.917 | 0.719 | 4.34E-18 | CD38+HLADR+ | SLC2A3 |
| VIM2 | 4.21E-41 | 0.417951068 | | 0.988 | 0.931 | 1.02E-36 | CD38+HLADR+ | VIM |
| NME1 | 4.96E-155 | 0.417700312 | | 0.397 | 0.083 | 1.21E-150 | CD38+HLADR+ | NME1 |
| ATP5F1D | 9.78E-54 | 0.417427506 | | 0.932 | 0.627 | 2.38E-49 | CD38+HLADR+ | ATP5F1D |
| CASP41 | 2.47E-99 | 0.416762974 | | 0.651 | 0.231 | 6.01E-95 | CD38+HLADR+ | CASP4 |
| IGFBP4 | 1.51E-195 | 0.416472417 | | 0.253 | 0.03 | 3.68E-191 | CD38+HLADR+ | IGFBP4 |
| CAPNS1 | 1.19E-69 | 0.416154087 | | 0.723 | 0.32 | 2.90E-65 | CD38+HLADR+ | CAPNS1 |
| GBP4 | 7.21E-151 | 0.416129965 | | 0.388 | 0.081 | 1.76E-146 | CD38+HLADR+ | GBP4 |
| TMEM50A1 | 1.90E-48 | 0.415691331 | | 0.84 | 0.486 | 4.62E-44 | CD38+HLADR+ | TMEM50A |
| TLN1 | 3.62E-73 | 0.413967702 | | 0.67 | 0.28 | 8.80E-69 | CD38+HLADR+ | TLN1 |
| MT-ND6 | 1.22E-63 | 0.413249593 | | 0.553 | 0.228 | 2.98E-59 | CD38+HLADR+ | MT-ND6 |
| GLRX | 1.66E-109 | 0.412967535 | | 0.422 | 0.116 | 4.04E-105 | CD38+HLADR+ | GLRX |
| TBCA | 1.10E-53 | 0.412265483 | | 0.822 | 0.448 | 2.69E-49 | CD38+HLADR+ | TBCA |
| TXNL4A | 5.32E-56 | 0.41210345 | | 0.757 | 0.377 | 1.29E-51 | CD38+HLADR+ | TXNL4A |
| SQOR | 4.44E-177 | 0.412086181 | | 0.496 | 0.107 | 1.08E-172 | CD38+HLADR+ | SQOR |
| UBE2K | 3.55E-68 | 0.411319237 | | 0.672 | 0.292 | 8.66E-64 | CD38+HLADR+ | UBE2K |
| MACF1 | 2.03E-72 | 0.411134591 | | 0.655 | 0.272 | 4.94E-68 | CD38+HLADR+ | MACF1 |
| PRKCB1 | 5.67E-57 | 0.410669783 | | 0.741 | 0.363 | 1.38E-52 | CD38+HLADR+ | PRKCB |
| RAC1 | 1.68E-47 | 0.410046274 | | 0.824 | 0.472 | 4.09E-43 | CD38+HLADR+ | RAC1 |
| CLTB | 4.08E-85 | 0.409577448 | | 0.686 | 0.265 | 9.94E-81 | CD38+HLADR+ | CLTB |
| TBL1XR1 | 6.59E-110 | 0.409260768 | | 0.516 | 0.155 | 1.60E-105 | CD38+HLADR+ | TBL1XR1 |
| CCM2 | 1.37E-71 | 0.408589449 | | 0.646 | 0.27 | 3.33E-67 | CD38+HLADR+ | CCM2 |
| SNRPF | 6.86E-56 | 0.408222029 | | 0.793 | 0.418 | 1.67E-51 | CD38+HLADR+ | SNRPF |
| RABGAP1L1 | 1.82E-53 | 0.408075422 | | 0.775 | 0.404 | 4.42E-49 | CD38+HLADR+ | RABGAP1L |
| SDHC | 1.50E-114 | 0.408031969 | | 0.575 | 0.176 | 3.65E-110 | CD38+HLADR+ | SDHC |
| COPZ1 | 4.34E-124 | 0.407951075 | | 0.511 | 0.142 | 1.06E-119 | CD38+HLADR+ | COPZ1 |
| CLTA | 7.42E-68 | 0.40786725 | | 0.663 | 0.287 | 1.81E-63 | CD38+HLADR+ | CLTA |
| TMEM167A | 1.38E-130 | 0.40770737 | | 0.496 | 0.132 | 3.36E-126 | CD38+HLADR+ | TMEM167A |
| LSM7 | 2.78E-52 | 0.40714396 | | 0.822 | 0.448 | 6.77E-48 | CD38+HLADR+ | LSM7 |
| TADA3 | 3.28E-137 | 0.406906325 | | 0.46 | 0.114 | 8.00E-133 | CD38+HLADR+ | TADA3 |
| SSH21 | 1.51E-45 | 0.40681321 | | 0.866 | 0.548 | 3.67E-41 | CD38+HLADR+ | SSH2 |
| ATP5PO | 1.30E-46 | 0.406404106 | | 0.913 | 0.617 | 3.17E-42 | CD38+HLADR+ | ATP5PO |
| HMGB2 | 8.08E-45 | 0.406092742 | | 0.825 | 0.475 | 1.97E-40 | CD38+HLADR+ | HMGB2 |
| LINC00426 | 9.79E-190 | 0.405844934 | | 0.332 | 0.051 | 2.38E-185 | CD38+HLADR+ | LINC00426 |
| GSDMD | 8.91E-139 | 0.405557822 | | 0.519 | 0.136 | 2.17E-134 | CD38+HLADR+ | GSDMD |
| SH3BGRL | 1.18E-52 | 0.405434135 | | 0.799 | 0.421 | 2.87E-48 | CD38+HLADR+ | SH3BGRL |
| RTRAF | 3.53E-47 | 0.405095414 | | 0.865 | 0.527 | 8.59E-43 | CD38+HLADR+ | RTRAF |
| GSPT1 | 2.27E-35 | 0.404330251 | | 0.868 | 0.617 | 5.53E-31 | CD38+HLADR+ | GSPT1 |
| MIEN1 | 1.22E-112 | 0.403670187 | | 0.557 | 0.171 | 2.97E-108 | CD38+HLADR+ | MIEN1 |
| PSMA6 | 3.97E-54 | 0.403575507 | | 0.765 | 0.39 | 9.67E-50 | CD38+HLADR+ | PSMA6 |
| ABI1 | 3.43E-64 | 0.403409043 | | 0.707 | 0.313 | 8.36E-60 | CD38+HLADR+ | ABI1 |
| IL12RB2 | 6.17E-109 | 0.402178525 | | 0.269 | 0.056 | 1.50E-104 | CD38+HLADR+ | IL12RB2 |
| HSD17B10 | 9.50E-154 | 0.401704467 | | 0.465 | 0.106 | 2.31E-149 | CD38+HLADR+ | HSD17B10 |
| WDFY1 | 9.09E-115 | 0.40057567 | | 0.374 | 0.092 | 2.21E-110 | CD38+HLADR+ | WDFY1 |
| TNIK | 2.29E-61 | 0.399952274 | | 0.69 | 0.321 | 5.57E-57 | CD38+HLADR+ | TNIK |
| SRPK2 | 1.48E-64 | 0.399809378 | | 0.702 | 0.318 | 3.61E-60 | CD38+HLADR+ | SRPK2 |
| ACOT9 | 1.16E-161 | 0.399681868 | | 0.401 | 0.081 | 2.82E-157 | CD38+HLADR+ | ACOT9 |
| AGPAT5 | 1.45E-139 | 0.399509047 | | 0.356 | 0.074 | 3.53E-135 | CD38+HLADR+ | AGPAT5 |
| MGAT51 | 5.27E-72 | 0.39942032 | | 0.507 | 0.19 | 1.28E-67 | CD38+HLADR+ | MGAT5 |
| ACP5 | 1.53E-231 | 0.397540062 | | 0.374 | 0.053 | 3.71E-227 | CD38+HLADR+ | ACP5 |
| SYNE22 | 2.50E-31 | 0.39692445 | | 0.902 | 0.621 | 6.08E-27 | CD38+HLADR+ | SYNE2 |
| GRAMD1B | 5.91E-194 | 0.396322383 | | 0.299 | 0.041 | 1.44E-189 | CD38+HLADR+ | GRAMD1B |
| MT-CYB1 | 3.58E-17 | 0.396047139 | | 0.985 | 0.948 | 8.71E-13 | CD38+HLADR+ | MT-CYB |
| SRP9 | 3.01E-50 | 0.39548741 | | 0.746 | 0.383 | 7.32E-46 | CD38+HLADR+ | SRP9 |
| BAX | 4.45E-49 | 0.39468847 | | 0.791 | 0.397 | 1.08E-44 | CD38+HLADR+ | BAX |
| NSMCE1 | 1.33E-132 | 0.394588724 | | 0.473 | 0.121 | 3.25E-128 | CD38+HLADR+ | NSMCE1 |
| CNIH1 | 1.06E-96 | 0.394204083 | | 0.579 | 0.196 | 2.58E-92 | CD38+HLADR+ | CNIH1 |
| SLC25A6 | 1.67E-45 | 0.393337081 | | 0.972 | 0.875 | 4.06E-41 | CD38+HLADR+ | SLC25A6 |
| TNFRSF1B1 | 3.73E-35 | 0.392996102 | | 0.545 | 0.284 | 9.09E-31 | CD38+HLADR+ | TNFRSF1B |
| RAD23B | 3.58E-85 | 0.392663749 | | 0.638 | 0.241 | 8.72E-81 | CD38+HLADR+ | RAD23B |
| TMEM59 | 9.92E-51 | 0.391753261 | | 0.838 | 0.462 | 2.42E-46 | CD38+HLADR+ | TMEM59 |
| NAP1L41 | 2.96E-44 | 0.389682066 | | 0.84 | 0.479 | 7.20E-40 | CD38+HLADR+ | NAP1L4 |
| UBE2F1 | 1.30E-88 | 0.389675112 | | 0.528 | 0.181 | 3.16E-84 | CD38+HLADR+ | UBE2F |
| HADHA | 4.56E-72 | 0.389122094 | | 0.66 | 0.274 | 1.11E-67 | CD38+HLADR+ | HADHA |
| TRPS1 | 8.86E-110 | 0.38899158 | | 0.365 | 0.092 | 2.16E-105 | CD38+HLADR+ | TRPS1 |
| PSMC5 | 1.68E-64 | 0.388606927 | | 0.679 | 0.294 | 4.09E-60 | CD38+HLADR+ | PSMC5 |
| ESYT1 | 2.06E-181 | 0.38820932 | | 0.386 | 0.069 | 5.02E-177 | CD38+HLADR+ | ESYT1 |
| ACAP2 | 3.85E-63 | 0.387837415 | | 0.651 | 0.284 | 9.38E-59 | CD38+HLADR+ | ACAP2 |
| ARHGAP30 | 8.17E-99 | 0.387514016 | | 0.508 | 0.161 | 1.99E-94 | CD38+HLADR+ | ARHGAP30 |
| SUMO2 | 8.83E-48 | 0.387321296 | | 0.957 | 0.778 | 2.15E-43 | CD38+HLADR+ | SUMO2 |
| RNPEPL1 | 2.08E-67 | 0.385928495 | | 0.65 | 0.275 | 5.08E-63 | CD38+HLADR+ | RNPEPL1 |
| SLC9A3R11 | 4.36E-63 | 0.38590184 | | 0.619 | 0.265 | 1.06E-58 | CD38+HLADR+ | SLC9A3R1 |
| TRAF3 | 2.36E-97 | 0.385844247 | | 0.533 | 0.175 | 5.75E-93 | CD38+HLADR+ | TRAF3 |
| PYM1 | 3.07E-98 | 0.38573656 | | 0.473 | 0.146 | 7.46E-94 | CD38+HLADR+ | PYM1 |
| SLC9A9 | 6.27E-141 | 0.385594644 | | 0.365 | 0.077 | 1.53E-136 | CD38+HLADR+ | SLC9A9 |
| MRPS36 | 2.88E-99 | 0.385123285 | | 0.568 | 0.189 | 7.02E-95 | CD38+HLADR+ | MRPS36 |
| TBC1D10C | 4.86E-56 | 0.384861606 | | 0.813 | 0.403 | 1.18E-51 | CD38+HLADR+ | TBC1D10C |
| ECH1 | 1.76E-80 | 0.383955 | | 0.63 | 0.24 | 4.30E-76 | CD38+HLADR+ | ECH1 |
| RAP1GDS1 | 5.69E-87 | 0.383541083 | | 0.504 | 0.171 | 1.38E-82 | CD38+HLADR+ | RAP1GDS1 |
| NEAT11 | 1.17E-27 | 0.383237855 | | 0.846 | 0.583 | 2.85E-23 | CD38+HLADR+ | NEAT1 |
| DAD1 | 3.64E-57 | 0.383189552 | | 0.722 | 0.342 | 8.85E-53 | CD38+HLADR+ | DAD1 |
| GDI2 | 1.78E-66 | 0.383069065 | | 0.655 | 0.28 | 4.34E-62 | CD38+HLADR+ | GDI2 |
| USP15 | 2.48E-37 | 0.383056532 | | 0.893 | 0.576 | 6.04E-33 | CD38+HLADR+ | USP15 |
| TBC1D4 | 5.76E-85 | 0.382650703 | | 0.346 | 0.099 | 1.40E-80 | CD38+HLADR+ | TBC1D4 |
| HDDC2 | 1.97E-138 | 0.38247705 | | 0.463 | 0.113 | 4.79E-134 | CD38+HLADR+ | HDDC2 |
| TIMM13 | 4.98E-117 | 0.381814631 | | 0.516 | 0.148 | 1.21E-112 | CD38+HLADR+ | TIMM13 |
| CXCR3 | 1.05E-91 | 0.38130415 | | 0.34 | 0.092 | 2.55E-87 | CD38+HLADR+ | CXCR3 |
| TXNDC17 | 3.82E-114 | 0.381115954 | | 0.496 | 0.142 | 9.31E-110 | CD38+HLADR+ | TXNDC17 |
| YARS1 | 1.59E-69 | 0.380220733 | | 0.575 | 0.23 | 3.88E-65 | CD38+HLADR+ | YARS |
| ABLIM1 | 2.69E-34 | 0.380165036 | | 0.737 | 0.432 | 6.55E-30 | CD38+HLADR+ | ABLIM1 |
| TUFM | 8.60E-59 | 0.379860597 | | 0.714 | 0.337 | 2.10E-54 | CD38+HLADR+ | TUFM |
| POLR2G | 1.77E-84 | 0.37913796 | | 0.667 | 0.251 | 4.31E-80 | CD38+HLADR+ | POLR2G |
| GBP1 | 1.80E-155 | 0.378774341 | | 0.369 | 0.072 | 4.37E-151 | CD38+HLADR+ | GBP1 |
| MMADHC | 1.38E-69 | 0.378689725 | | 0.666 | 0.279 | 3.35E-65 | CD38+HLADR+ | MMADHC |
| OTUB1 | 6.98E-58 | 0.378654585 | | 0.755 | 0.362 | 1.70E-53 | CD38+HLADR+ | OTUB1 |
| LRRC8D | 6.27E-183 | 0.37859839 | | 0.366 | 0.063 | 1.53E-178 | CD38+HLADR+ | LRRC8D |
| SPPL2A | 1.05E-98 | 0.378135853 | | 0.479 | 0.146 | 2.56E-94 | CD38+HLADR+ | SPPL2A |
| RBX1 | 1.55E-55 | 0.377932204 | | 0.747 | 0.366 | 3.78E-51 | CD38+HLADR+ | RBX1 |
| TMEM14B | 1.22E-64 | 0.377473411 | | 0.734 | 0.33 | 2.97E-60 | CD38+HLADR+ | TMEM14B |
| TMBIM4 | 2.16E-86 | 0.377303652 | | 0.652 | 0.241 | 5.25E-82 | CD38+HLADR+ | TMBIM4 |
| SMIM7 | 5.53E-131 | 0.376808653 | | 0.491 | 0.128 | 1.35E-126 | CD38+HLADR+ | SMIM7 |
| LAPTM4A | 1.40E-69 | 0.376281786 | | 0.663 | 0.277 | 3.41E-65 | CD38+HLADR+ | LAPTM4A |
| SAE1 | 7.06E-127 | 0.375558518 | | 0.456 | 0.117 | 1.72E-122 | CD38+HLADR+ | SAE1 |
| MAP4K1 | 7.37E-112 | 0.375436256 | | 0.523 | 0.156 | 1.80E-107 | CD38+HLADR+ | MAP4K1 |
| NDUFA9 | 3.43E-99 | 0.375117658 | | 0.557 | 0.18 | 8.34E-95 | CD38+HLADR+ | NDUFA9 |
| COPS9 | 1.61E-58 | 0.374862447 | | 0.766 | 0.364 | 3.92E-54 | CD38+HLADR+ | COPS9 |
| NDUFAB1 | 6.02E-72 | 0.374380959 | | 0.666 | 0.273 | 1.47E-67 | CD38+HLADR+ | NDUFAB1 |
| PIK3R5 | 9.43E-48 | 0.374056855 | | 0.655 | 0.327 | 2.30E-43 | CD38+HLADR+ | PIK3R5 |
| LAMTOR1 | 1.38E-63 | 0.374019486 | | 0.717 | 0.318 | 3.37E-59 | CD38+HLADR+ | LAMTOR1 |
| TBL1X | 1.71E-74 | 0.373909338 | | 0.523 | 0.196 | 4.16E-70 | CD38+HLADR+ | TBL1X |
| TNFRSF14 | 3.19E-53 | 0.373260762 | | 0.77 | 0.382 | 7.76E-49 | CD38+HLADR+ | TNFRSF14 |
| RIPOR2 | 8.61E-40 | 0.373192276 | | 0.803 | 0.468 | 2.10E-35 | CD38+HLADR+ | RIPOR2 |
| THOC7 | 3.30E-108 | 0.37287261 | | 0.555 | 0.172 | 8.04E-104 | CD38+HLADR+ | THOC7 |
| PSMD9 | 5.31E-107 | 0.372731546 | | 0.588 | 0.188 | 1.29E-102 | CD38+HLADR+ | PSMD9 |
| CTDNEP1 | 7.91E-65 | 0.372359826 | | 0.68 | 0.292 | 1.93E-60 | CD38+HLADR+ | CTDNEP1 |
| NUDT5 | 4.91E-161 | 0.371256505 | | 0.381 | 0.074 | 1.20E-156 | CD38+HLADR+ | NUDT5 |
| UBL7 | 1.15E-150 | 0.371233521 | | 0.424 | 0.093 | 2.79E-146 | CD38+HLADR+ | UBL7 |
| HADHB | 2.25E-110 | 0.371203261 | | 0.475 | 0.136 | 5.49E-106 | CD38+HLADR+ | HADHB |
| BAK1 | 2.04E-147 | 0.370763517 | | 0.398 | 0.085 | 4.97E-143 | CD38+HLADR+ | BAK1 |
| BTF3 | 1.47E-68 | 0.370550903 | | 0.992 | 0.975 | 3.58E-64 | CD38+HLADR+ | BTF3 |
| DYNLRB1 | 1.53E-58 | 0.370503995 | | 0.659 | 0.298 | 3.72E-54 | CD38+HLADR+ | DYNLRB1 |
| CD2 | 1.81E-46 | 0.370355587 | | 0.866 | 0.489 | 4.41E-42 | CD38+HLADR+ | CD2 |
| CAPN2 | 4.99E-65 | 0.369408165 | | 0.662 | 0.282 | 1.21E-60 | CD38+HLADR+ | CAPN2 |
| COMMD4 | 8.54E-193 | 0.36928414 | | 0.385 | 0.065 | 2.08E-188 | CD38+HLADR+ | COMMD4 |
| ANAPC16 | 2.42E-44 | 0.369278322 | | 0.858 | 0.51 | 5.88E-40 | CD38+HLADR+ | ANAPC16 |
| FOXN2 | 3.22E-76 | 0.368901998 | | 0.552 | 0.208 | 7.83E-72 | CD38+HLADR+ | FOXN2 |
| OLA1 | 1.10E-72 | 0.368703094 | | 0.61 | 0.241 | 2.67E-68 | CD38+HLADR+ | OLA1 |
| CIAO2A | 2.08E-107 | 0.368367105 | | 0.563 | 0.176 | 5.07E-103 | CD38+HLADR+ | CIAO2A |
| CLEC2B1 | 3.69E-32 | 0.36832687 | | 0.791 | 0.49 | 8.99E-28 | CD38+HLADR+ | CLEC2B |
| RABL6 | 2.69E-121 | 0.368063449 | | 0.492 | 0.135 | 6.56E-117 | CD38+HLADR+ | RABL6 |
| INPP5D | 1.69E-56 | 0.368011916 | | 0.566 | 0.25 | 4.11E-52 | CD38+HLADR+ | INPP5D |
| EIF4E2 | 5.54E-100 | 0.367675014 | | 0.525 | 0.166 | 1.35E-95 | CD38+HLADR+ | EIF4E2 |
| PTPN2 | 2.13E-62 | 0.367194493 | | 0.607 | 0.258 | 5.19E-58 | CD38+HLADR+ | PTPN2 |
| C16orf87 | 1.61E-122 | 0.366586153 | | 0.436 | 0.112 | 3.92E-118 | CD38+HLADR+ | C16orf87 |
| HIGD1A | 2.02E-125 | 0.366155919 | | 0.451 | 0.115 | 4.92E-121 | CD38+HLADR+ | HIGD1A |
| CDC25B | 2.23E-82 | 0.365357872 | | 0.5 | 0.173 | 5.44E-78 | CD38+HLADR+ | CDC25B |
| GNB1 | 1.76E-50 | 0.365198861 | | 0.755 | 0.377 | 4.28E-46 | CD38+HLADR+ | GNB1 |
| RALA | 1.06E-64 | 0.364667239 | | 0.608 | 0.258 | 2.57E-60 | CD38+HLADR+ | RALA |
| TMEM1231 | 2.52E-38 | 0.364200533 | | 0.858 | 0.558 | 6.14E-34 | CD38+HLADR+ | TMEM123 |
| UQCRQ | 1.22E-51 | 0.364124452 | | 0.778 | 0.396 | 2.98E-47 | CD38+HLADR+ | UQCRQ |
| LRBA | 6.79E-56 | 0.363223597 | | 0.697 | 0.326 | 1.65E-51 | CD38+HLADR+ | LRBA |
| ANAPC15 | 6.72E-102 | 0.362267246 | | 0.505 | 0.155 | 1.64E-97 | CD38+HLADR+ | ANAPC15 |
| NDUFS2 | 7.19E-93 | 0.361870748 | | 0.574 | 0.194 | 1.75E-88 | CD38+HLADR+ | NDUFS2 |
| PSMB1 | 6.95E-40 | 0.361731329 | | 0.886 | 0.588 | 1.69E-35 | CD38+HLADR+ | PSMB1 |
| SCAND1 | 8.76E-38 | 0.361110275 | | 0.898 | 0.58 | 2.13E-33 | CD38+HLADR+ | SCAND1 |
| PMAIP11 | 2.49E-42 | 0.360859272 | | 0.662 | 0.347 | 6.06E-38 | CD38+HLADR+ | PMAIP1 |
| UHRF2 | 2.43E-82 | 0.360675637 | | 0.505 | 0.175 | 5.93E-78 | CD38+HLADR+ | UHRF2 |
| BCAP31 | 2.52E-64 | 0.360527146 | | 0.691 | 0.301 | 6.13E-60 | CD38+HLADR+ | BCAP31 |
| DEK | 7.17E-52 | 0.360283708 | | 0.729 | 0.362 | 1.75E-47 | CD38+HLADR+ | DEK |
| RPS6KA1 | 1.92E-172 | 0.359987805 | | 0.372 | 0.067 | 4.68E-168 | CD38+HLADR+ | RPS6KA1 |
| LINC008611 | 1.77E-66 | 0.359972255 | | 0.612 | 0.257 | 4.30E-62 | CD38+HLADR+ | LINC00861 |
| IRF7 | 2.28E-67 | 0.359889861 | | 0.422 | 0.151 | 5.56E-63 | CD38+HLADR+ | IRF7 |
| LAMTOR4 | 7.90E-44 | 0.359591605 | | 0.852 | 0.503 | 1.92E-39 | CD38+HLADR+ | LAMTOR4 |
| TAP2 | 9.31E-76 | 0.359218139 | | 0.519 | 0.189 | 2.27E-71 | CD38+HLADR+ | TAP2 |
| NDUFB1 | 5.79E-73 | 0.35885718 | | 0.607 | 0.236 | 1.41E-68 | CD38+HLADR+ | NDUFB1 |
| GRB2 | 3.92E-52 | 0.358544129 | | 0.702 | 0.344 | 9.56E-48 | CD38+HLADR+ | GRB2 |
| MRPL37 | 3.20E-151 | 0.3583139 | | 0.424 | 0.092 | 7.80E-147 | CD38+HLADR+ | MRPL37 |
| DR1 | 5.88E-89 | 0.357992503 | | 0.549 | 0.186 | 1.43E-84 | CD38+HLADR+ | DR1 |
| IKBKE | 1.01E-145 | 0.357883816 | | 0.369 | 0.076 | 2.45E-141 | CD38+HLADR+ | IKBKE |
| GDE1 | 3.92E-89 | 0.357628405 | | 0.333 | 0.091 | 9.54E-85 | CD38+HLADR+ | GDE1 |
| SUSD3 | 2.89E-115 | 0.357406763 | | 0.452 | 0.123 | 7.04E-111 | CD38+HLADR+ | SUSD3 |
| SRP14 | 3.57E-48 | 0.356975347 | | 0.975 | 0.869 | 8.69E-44 | CD38+HLADR+ | SRP14 |
| RAB1B1 | 5.79E-71 | 0.356925602 | | 0.607 | 0.242 | 1.41E-66 | CD38+HLADR+ | RAB1B |
| FAS | 3.16E-72 | 0.356792331 | | 0.559 | 0.213 | 7.69E-68 | CD38+HLADR+ | FAS |
| EID11 | 1.58E-40 | 0.356699787 | | 0.857 | 0.508 | 3.84E-36 | CD38+HLADR+ | EID1 |
| CTSC1 | 1.44E-62 | 0.355928777 | | 0.576 | 0.239 | 3.50E-58 | CD38+HLADR+ | CTSC |
| PGM2L1 | 8.02E-115 | 0.355647519 | | 0.286 | 0.059 | 1.95E-110 | CD38+HLADR+ | PGM2L1 |
| TFDP1 | 8.03E-104 | 0.355592709 | | 0.445 | 0.127 | 1.96E-99 | CD38+HLADR+ | TFDP1 |
| ATP2A3 | 1.99E-85 | 0.355566941 | | 0.556 | 0.194 | 4.84E-81 | CD38+HLADR+ | ATP2A3 |
| ATF7IP2 | 5.99E-68 | 0.355549584 | | 0.591 | 0.24 | 1.46E-63 | CD38+HLADR+ | ATF7IP2 |
| STK17A | 7.17E-30 | 0.354829261 | | 0.902 | 0.678 | 1.75E-25 | CD38+HLADR+ | STK17A |
| SMARCA2 | 6.44E-48 | 0.354800217 | | 0.702 | 0.346 | 1.57E-43 | CD38+HLADR+ | SMARCA2 |
| LSM4 | 1.02E-70 | 0.354706847 | | 0.553 | 0.212 | 2.49E-66 | CD38+HLADR+ | LSM4 |
| LRP10 | 8.18E-72 | 0.354231797 | | 0.595 | 0.233 | 1.99E-67 | CD38+HLADR+ | LRP10 |
| PTEN | 1.93E-87 | 0.353601528 | | 0.551 | 0.191 | 4.70E-83 | CD38+HLADR+ | PTEN |
| SSBP1 | 8.68E-66 | 0.352729008 | | 0.668 | 0.283 | 2.11E-61 | CD38+HLADR+ | SSBP1 |
| DNM2 | 2.91E-66 | 0.3525557 | | 0.603 | 0.247 | 7.10E-62 | CD38+HLADR+ | DNM2 |
| TMEM160 | 8.91E-85 | 0.352108591 | | 0.575 | 0.204 | 2.17E-80 | CD38+HLADR+ | TMEM160 |
| ANAPC11 | 4.93E-74 | 0.351894718 | | 0.566 | 0.216 | 1.20E-69 | CD38+HLADR+ | ANAPC11 |
| SHISA5 | 1.74E-57 | 0.351850135 | | 0.694 | 0.308 | 4.24E-53 | CD38+HLADR+ | SHISA5 |
| DLEU2 | 8.95E-78 | 0.351748957 | | 0.425 | 0.141 | 2.18E-73 | CD38+HLADR+ | DLEU2 |
| PDCL3 | 3.77E-90 | 0.351434333 | | 0.497 | 0.163 | 9.19E-86 | CD38+HLADR+ | PDCL3 |
| GHITM | 3.63E-48 | 0.351234112 | | 0.703 | 0.352 | 8.84E-44 | CD38+HLADR+ | GHITM |
| SLC16A1 | 2.29E-97 | 0.350790403 | | 0.311 | 0.077 | 5.57E-93 | CD38+HLADR+ | SLC16A1 |
| TRAF3IP3 | 7.56E-60 | 0.350756601 | | 0.684 | 0.307 | 1.84E-55 | CD38+HLADR+ | TRAF3IP3 |
| OSTF1 | 4.72E-55 | 0.350637544 | | 0.639 | 0.29 | 1.15E-50 | CD38+HLADR+ | OSTF1 |
| FLNB | 1.33E-167 | 0.350534567 | | 0.271 | 0.039 | 3.25E-163 | CD38+HLADR+ | FLNB |
| CD79B | 7.89E-203 | 0.349758877 | | 0.274 | 0.033 | 1.92E-198 | CD38+HLADR+ | CD79B |
| PHF19 | 2.22E-139 | 0.349739471 | | 0.365 | 0.077 | 5.40E-135 | CD38+HLADR+ | PHF19 |
| PPP2CA | 1.24E-48 | 0.349552048 | | 0.773 | 0.389 | 3.03E-44 | CD38+HLADR+ | PPP2CA |
| DIP2B | 1.83E-96 | 0.349515521 | | 0.392 | 0.109 | 4.45E-92 | CD38+HLADR+ | DIP2B |
| BORCS5 | 2.43E-72 | 0.349510062 | | 0.532 | 0.2 | 5.92E-68 | CD38+HLADR+ | BORCS5 |
| CCDC200 | 1.98E-48 | 0.34893358 | | 0.279 | 0.098 | 4.81E-44 | CD38+HLADR+ | CCDC200 |
| LTB2 | 3.58E-16 | 0.348405996 | | 0.874 | 0.671 | 8.71E-12 | CD38+HLADR+ | LTB |
| TAF10 | 1.68E-37 | 0.348294889 | | 0.893 | 0.61 | 4.08E-33 | CD38+HLADR+ | TAF10 |
| CCDC88C | 7.44E-57 | 0.348234446 | | 0.672 | 0.303 | 1.81E-52 | CD38+HLADR+ | CCDC88C |
| YBX1 | 7.79E-37 | 0.348163319 | | 0.969 | 0.828 | 1.90E-32 | CD38+HLADR+ | YBX1 |
| ATOX1 | 4.24E-101 | 0.347983069 | | 0.418 | 0.118 | 1.03E-96 | CD38+HLADR+ | ATOX1 |
| POLR1D | 8.23E-39 | 0.347979863 | | 0.888 | 0.543 | 2.00E-34 | CD38+HLADR+ | POLR1D |
| INPP5K | 6.15E-96 | 0.347900549 | | 0.513 | 0.163 | 1.50E-91 | CD38+HLADR+ | INPP5K |
| COX7A2L | 2.67E-49 | 0.347605718 | | 0.775 | 0.398 | 6.49E-45 | CD38+HLADR+ | COX7A2L |
| RANBP9 | 1.00E-80 | 0.34757216 | | 0.571 | 0.207 | 2.44E-76 | CD38+HLADR+ | RANBP9 |
| YIF1B | 1.76E-166 | 0.34752662 | | 0.364 | 0.066 | 4.30E-162 | CD38+HLADR+ | YIF1B |
| PEPD | 1.12E-104 | 0.347486178 | | 0.389 | 0.103 | 2.72E-100 | CD38+HLADR+ | PEPD |
| SYTL1 | 3.22E-53 | 0.347210492 | | 0.66 | 0.309 | 7.83E-49 | CD38+HLADR+ | SYTL1 |
| FKBP3 | 9.70E-117 | 0.347192946 | | 0.408 | 0.104 | 2.36E-112 | CD38+HLADR+ | FKBP3 |
| ATP5MD | 8.11E-37 | 0.347020259 | | 0.866 | 0.552 | 1.98E-32 | CD38+HLADR+ | ATP5MD |
| CDK2AP2 | 5.84E-75 | 0.346953636 | | 0.424 | 0.142 | 1.42E-70 | CD38+HLADR+ | CDK2AP2 |
| PDIA61 | 3.33E-61 | 0.346402626 | | 0.576 | 0.24 | 8.10E-57 | CD38+HLADR+ | PDIA6 |
| STAM | 7.97E-81 | 0.346382406 | | 0.441 | 0.146 | 1.94E-76 | CD38+HLADR+ | STAM |
| NT5C | 1.12E-82 | 0.346374123 | | 0.505 | 0.174 | 2.72E-78 | CD38+HLADR+ | NT5C |
| NCOA3 | 9.46E-88 | 0.346214019 | | 0.453 | 0.144 | 2.30E-83 | CD38+HLADR+ | NCOA3 |
| PPP1R12A | 1.29E-53 | 0.34554768 | | 0.675 | 0.313 | 3.15E-49 | CD38+HLADR+ | PPP1R12A |
| KRT101 | 4.89E-42 | 0.345156782 | | 0.834 | 0.484 | 1.19E-37 | CD38+HLADR+ | KRT10 |
| ATP6V0D1 | 2.11E-104 | 0.344951358 | | 0.519 | 0.158 | 5.13E-100 | CD38+HLADR+ | ATP6V0D1 |
| ETFA | 8.18E-104 | 0.344714976 | | 0.521 | 0.159 | 1.99E-99 | CD38+HLADR+ | ETFA |
| MED13L | 2.66E-78 | 0.344637493 | | 0.551 | 0.201 | 6.48E-74 | CD38+HLADR+ | MED13L |
| CEP128 | 1.47E-109 | 0.343870166 | | 0.257 | 0.051 | 3.58E-105 | CD38+HLADR+ | CEP128 |
| RICTOR | 5.19E-41 | 0.343613604 | | 0.723 | 0.384 | 1.26E-36 | CD38+HLADR+ | RICTOR |
| IFI35 | 1.31E-114 | 0.343379375 | | 0.349 | 0.082 | 3.18E-110 | CD38+HLADR+ | IFI35 |
| TMEM173 | 5.95E-56 | 0.343218037 | | 0.521 | 0.22 | 1.45E-51 | CD38+HLADR+ | TMEM173 |
| OGT | 8.54E-70 | 0.343027069 | | 0.584 | 0.233 | 2.08E-65 | CD38+HLADR+ | OGT |
| UBR5 | 1.59E-64 | 0.34267776 | | 0.556 | 0.224 | 3.88E-60 | CD38+HLADR+ | UBR5 |
| EIF5A | 3.39E-33 | 0.341982164 | | 0.906 | 0.654 | 8.26E-29 | CD38+HLADR+ | EIF5A |
| LAMTOR5 | 5.26E-63 | 0.341940841 | | 0.697 | 0.297 | 1.28E-58 | CD38+HLADR+ | LAMTOR5 |
| ATP5MPL | 5.23E-43 | 0.341778732 | | 0.801 | 0.451 | 1.27E-38 | CD38+HLADR+ | ATP5MPL |
| SNX3 | 2.13E-53 | 0.341560452 | | 0.694 | 0.322 | 5.19E-49 | CD38+HLADR+ | SNX3 |
| TET2 | 3.28E-97 | 0.341543959 | | 0.385 | 0.106 | 7.99E-93 | CD38+HLADR+ | TET2 |
| ANKRD11 | 1.65E-43 | 0.341399095 | | 0.791 | 0.428 | 4.02E-39 | CD38+HLADR+ | ANKRD11 |
| GPI | 1.67E-49 | 0.340822374 | | 0.686 | 0.329 | 4.08E-45 | CD38+HLADR+ | GPI |
| SAMSN12 | 2.73E-38 | 0.340818105 | | 0.734 | 0.409 | 6.66E-34 | CD38+HLADR+ | SAMSN1 |
| DENND2D | 1.62E-90 | 0.340570317 | | 0.461 | 0.145 | 3.94E-86 | CD38+HLADR+ | DENND2D |
| PCBD1 | 3.60E-182 | 0.34016207 | | 0.329 | 0.052 | 8.76E-178 | CD38+HLADR+ | PCBD1 |
| CMTM3 | 1.51E-61 | 0.339682589 | | 0.57 | 0.234 | 3.69E-57 | CD38+HLADR+ | CMTM3 |
| FUT8 | 2.53E-108 | 0.339299865 | | 0.38 | 0.097 | 6.16E-104 | CD38+HLADR+ | FUT8 |
| RER1 | 4.43E-88 | 0.338674488 | | 0.507 | 0.169 | 1.08E-83 | CD38+HLADR+ | RER1 |
| DCTN2 | 3.16E-87 | 0.338196805 | | 0.536 | 0.181 | 7.70E-83 | CD38+HLADR+ | DCTN2 |
| BSG1 | 2.04E-59 | 0.338145628 | | 0.702 | 0.313 | 4.97E-55 | CD38+HLADR+ | BSG |
| YWHAZ | 1.63E-44 | 0.338058587 | | 0.991 | 0.861 | 3.98E-40 | CD38+HLADR+ | YWHAZ |
| ATP6V0E1 | 2.24E-40 | 0.338041482 | | 0.806 | 0.438 | 5.47E-36 | CD38+HLADR+ | ATP6V0E1 |
| TSPAN5 | 2.40E-90 | 0.337495194 | | 0.344 | 0.094 | 5.85E-86 | CD38+HLADR+ | TSPAN5 |
| CDC42 | 2.79E-39 | 0.336383675 | | 0.957 | 0.764 | 6.79E-35 | CD38+HLADR+ | CDC42 |
| LAP3 | 2.33E-89 | 0.336371556 | | 0.501 | 0.164 | 5.67E-85 | CD38+HLADR+ | LAP3 |
| SEC61G | 2.57E-31 | 0.335835048 | | 0.857 | 0.567 | 6.26E-27 | CD38+HLADR+ | SEC61G |
| CTSS | 2.89E-72 | 0.335669664 | | 0.614 | 0.24 | 7.03E-68 | CD38+HLADR+ | CTSS |
| RAB11B | 2.89E-57 | 0.335174538 | | 0.727 | 0.33 | 7.04E-53 | CD38+HLADR+ | RAB11B |
| FNBP11 | 1.75E-32 | 0.334759247 | | 0.886 | 0.586 | 4.26E-28 | CD38+HLADR+ | FNBP1 |
| POLR3GL | 1.20E-71 | 0.334663902 | | 0.564 | 0.216 | 2.93E-67 | CD38+HLADR+ | POLR3GL |
| RTF2 | 6.29E-80 | 0.334252539 | | 0.547 | 0.194 | 1.53E-75 | CD38+HLADR+ | RTF2 |
| BIN21 | 6.57E-41 | 0.3338115 | | 0.816 | 0.463 | 1.60E-36 | CD38+HLADR+ | BIN2 |
| PDCD2 | 2.26E-85 | 0.333195328 | | 0.529 | 0.181 | 5.49E-81 | CD38+HLADR+ | PDCD2 |
| CHCHD2 | 2.56E-39 | 0.333110861 | | 0.979 | 0.86 | 6.23E-35 | CD38+HLADR+ | CHCHD2 |
| CPNE1 | 8.74E-83 | 0.33306932 | | 0.509 | 0.175 | 2.13E-78 | CD38+HLADR+ | CPNE1 |
| MTERF4 | 4.62E-74 | 0.3328227 | | 0.58 | 0.221 | 1.13E-69 | CD38+HLADR+ | MTERF4 |
| PSMG2 | 1.32E-57 | 0.332548802 | | 0.675 | 0.298 | 3.22E-53 | CD38+HLADR+ | PSMG2 |
| CCT5 | 3.07E-119 | 0.332181367 | | 0.413 | 0.104 | 7.49E-115 | CD38+HLADR+ | CCT5 |
| RIOK3 | 2.87E-63 | 0.331750475 | | 0.668 | 0.284 | 6.99E-59 | CD38+HLADR+ | RIOK3 |
| CAPZA1 | 2.67E-47 | 0.331319206 | | 0.762 | 0.392 | 6.50E-43 | CD38+HLADR+ | CAPZA1 |
| FCMR | 1.21E-29 | 0.331129334 | | 0.643 | 0.363 | 2.96E-25 | CD38+HLADR+ | FCMR |
| CMTM61 | 1.08E-58 | 0.33068609 | | 0.594 | 0.248 | 2.62E-54 | CD38+HLADR+ | CMTM6 |
| LAPTM5 | 8.03E-31 | 0.330528884 | | 0.979 | 0.871 | 1.96E-26 | CD38+HLADR+ | LAPTM5 |
| PSMB7 | 2.92E-71 | 0.33024364 | | 0.611 | 0.24 | 7.10E-67 | CD38+HLADR+ | PSMB7 |
| ACAP1 | 8.30E-43 | 0.330222693 | | 0.841 | 0.472 | 2.02E-38 | CD38+HLADR+ | ACAP1 |
| VMA21 | 3.49E-124 | 0.330150424 | | 0.428 | 0.106 | 8.50E-120 | CD38+HLADR+ | VMA21 |
| SNRPC | 2.26E-52 | 0.330025895 | | 0.694 | 0.326 | 5.50E-48 | CD38+HLADR+ | SNRPC |
| GGCT | 4.50E-154 | 0.330023948 | | 0.314 | 0.055 | 1.10E-149 | CD38+HLADR+ | GGCT |
| STX11 | 3.06E-91 | 0.329800297 | | 0.348 | 0.095 | 7.45E-87 | CD38+HLADR+ | STX11 |
| XRN21 | 2.98E-53 | 0.329714421 | | 0.702 | 0.329 | 7.27E-49 | CD38+HLADR+ | XRN2 |
| MYL12A | 1.08E-39 | 0.329670857 | | 0.985 | 0.888 | 2.64E-35 | CD38+HLADR+ | MYL12A |
| SERINC52 | 1.29E-51 | 0.329290126 | | 0.616 | 0.281 | 3.14E-47 | CD38+HLADR+ | SERINC5 |
| NUCB1 | 9.26E-65 | 0.329163997 | | 0.644 | 0.269 | 2.26E-60 | CD38+HLADR+ | NUCB1 |
| SNF8 | 4.94E-84 | 0.328598725 | | 0.512 | 0.173 | 1.20E-79 | CD38+HLADR+ | SNF8 |
| TMED3 | 3.41E-124 | 0.328190374 | | 0.434 | 0.109 | 8.31E-120 | CD38+HLADR+ | TMED3 |
| RUVBL1 | 8.31E-133 | 0.327997993 | | 0.332 | 0.068 | 2.02E-128 | CD38+HLADR+ | RUVBL1 |
| HPRT1 | 7.27E-103 | 0.327983109 | | 0.448 | 0.127 | 1.77E-98 | CD38+HLADR+ | HPRT1 |
| RBCK1 | 8.87E-66 | 0.327890035 | | 0.602 | 0.242 | 2.16E-61 | CD38+HLADR+ | RBCK1 |
| NARF | 2.19E-89 | 0.327772707 | | 0.467 | 0.148 | 5.33E-85 | CD38+HLADR+ | NARF |
| CDC123 | 5.71E-72 | 0.327525592 | | 0.543 | 0.203 | 1.39E-67 | CD38+HLADR+ | CDC123 |
| EML41 | 8.61E-25 | 0.327054036 | | 0.917 | 0.692 | 2.10E-20 | CD38+HLADR+ | EML4 |
| IQGAP21 | 3.49E-36 | 0.327024045 | | 0.79 | 0.458 | 8.49E-32 | CD38+HLADR+ | IQGAP2 |
| SP140 | 6.42E-57 | 0.327023464 | | 0.58 | 0.249 | 1.56E-52 | CD38+HLADR+ | SP140 |
| HELZ | 9.54E-62 | 0.327021907 | | 0.595 | 0.248 | 2.32E-57 | CD38+HLADR+ | HELZ |
| HSPE1 | 2.61E-35 | 0.326826367 | | 0.805 | 0.47 | 6.36E-31 | CD38+HLADR+ | HSPE1 |
| LY96 | 2.64E-156 | 0.326678571 | | 0.309 | 0.052 | 6.42E-152 | CD38+HLADR+ | LY96 |
| FKBP51 | 9.83E-30 | 0.326620291 | | 0.781 | 0.503 | 2.39E-25 | CD38+HLADR+ | FKBP5 |
| HACD4 | 1.26E-76 | 0.326437416 | | 0.418 | 0.137 | 3.06E-72 | CD38+HLADR+ | HACD4 |
| TIMP1 | 7.93E-32 | 0.326304677 | | 0.444 | 0.226 | 1.93E-27 | CD38+HLADR+ | TIMP1 |
| ANP32B | 1.64E-34 | 0.32623462 | | 0.912 | 0.69 | 4.00E-30 | CD38+HLADR+ | ANP32B |
| ACAA1 | 3.31E-94 | 0.325849217 | | 0.484 | 0.151 | 8.06E-90 | CD38+HLADR+ | ACAA1 |
| NDUFB5 | 1.33E-95 | 0.325637022 | | 0.464 | 0.141 | 3.23E-91 | CD38+HLADR+ | NDUFB5 |
| FRMD4B | 2.56E-115 | 0.325394553 | | 0.29 | 0.06 | 6.25E-111 | CD38+HLADR+ | FRMD4B |
| VOPP1 | 2.45E-65 | 0.325387033 | | 0.642 | 0.266 | 5.97E-61 | CD38+HLADR+ | VOPP1 |
| NUDT21 | 1.51E-63 | 0.325248008 | | 0.636 | 0.263 | 3.68E-59 | CD38+HLADR+ | NUDT21 |
| SUCLG1 | 6.84E-99 | 0.325157742 | | 0.46 | 0.136 | 1.67E-94 | CD38+HLADR+ | SUCLG1 |
| SVBP | 4.76E-99 | 0.325127574 | | 0.428 | 0.122 | 1.16E-94 | CD38+HLADR+ | SVBP |
| MAP1LC3B1 | 4.17E-39 | 0.324943365 | | 0.86 | 0.516 | 1.01E-34 | CD38+HLADR+ | MAP1LC3B |
| DEAF1 | 3.39E-115 | 0.324507072 | | 0.374 | 0.091 | 8.25E-111 | CD38+HLADR+ | DEAF1 |
| HPCAL1 | 4.20E-89 | 0.324301859 | | 0.491 | 0.158 | 1.02E-84 | CD38+HLADR+ | HPCAL1 |
| PIN1 | 1.21E-72 | 0.323622783 | | 0.576 | 0.216 | 2.93E-68 | CD38+HLADR+ | PIN1 |
| DUSP52 | 2.63E-39 | 0.323619516 | | 0.608 | 0.313 | 6.40E-35 | CD38+HLADR+ | DUSP5 |
| ARL6IP5 | 4.43E-29 | 0.323456417 | | 0.866 | 0.56 | 1.08E-24 | CD38+HLADR+ | ARL6IP5 |
| SMARCE1 | 2.24E-59 | 0.323215342 | | 0.623 | 0.262 | 5.45E-55 | CD38+HLADR+ | SMARCE1 |
| SNX5 | 4.54E-81 | 0.32319171 | | 0.508 | 0.174 | 1.11E-76 | CD38+HLADR+ | SNX5 |
| CLNS1A | 7.47E-70 | 0.322949829 | | 0.594 | 0.231 | 1.82E-65 | CD38+HLADR+ | CLNS1A |
| BRD7 | 8.08E-58 | 0.322665401 | | 0.531 | 0.219 | 1.97E-53 | CD38+HLADR+ | BRD7 |
| EIF3F | 1.64E-33 | 0.322646976 | | 0.94 | 0.841 | 4.00E-29 | CD38+HLADR+ | EIF3F |
| LYPLA2 | 4.59E-121 | 0.32255476 | | 0.408 | 0.1 | 1.12E-116 | CD38+HLADR+ | LYPLA2 |
| STX10 | 5.32E-107 | 0.32218001 | | 0.481 | 0.138 | 1.30E-102 | CD38+HLADR+ | STX10 |
| HMGN1 | 3.25E-33 | 0.322064288 | | 0.853 | 0.531 | 7.91E-29 | CD38+HLADR+ | HMGN1 |
| EIF6 | 1.33E-103 | 0.322009138 | | 0.493 | 0.146 | 3.24E-99 | CD38+HLADR+ | EIF6 |
| SAT12 | 1.05E-18 | 0.321841457 | | 0.968 | 0.802 | 2.56E-14 | CD38+HLADR+ | SAT1 |
| BNIP3L | 6.15E-73 | 0.321615823 | | 0.59 | 0.225 | 1.50E-68 | CD38+HLADR+ | BNIP3L |
| MRPS15 | 9.65E-63 | 0.321255096 | | 0.654 | 0.274 | 2.35E-58 | CD38+HLADR+ | MRPS15 |
| PSMD13 | 3.03E-64 | 0.321022544 | | 0.54 | 0.214 | 7.39E-60 | CD38+HLADR+ | PSMD13 |
| WASF2 | 1.19E-50 | 0.320865415 | | 0.727 | 0.349 | 2.91E-46 | CD38+HLADR+ | WASF2 |
| NAP1L1 | 2.32E-34 | 0.320859847 | | 0.896 | 0.621 | 5.65E-30 | CD38+HLADR+ | NAP1L1 |
| NDUFA10 | 6.75E-73 | 0.320787737 | | 0.571 | 0.215 | 1.64E-68 | CD38+HLADR+ | NDUFA10 |
| POLR2L | 1.15E-49 | 0.320118903 | | 0.783 | 0.395 | 2.81E-45 | CD38+HLADR+ | POLR2L |
| NOA1 | 8.18E-117 | 0.31997114 | | 0.409 | 0.103 | 1.99E-112 | CD38+HLADR+ | NOA1 |
| HAPLN3 | 6.35E-127 | 0.31943566 | | 0.338 | 0.072 | 1.55E-122 | CD38+HLADR+ | HAPLN3 |
| SLC25A46 | 1.50E-115 | 0.318113222 | | 0.358 | 0.085 | 3.65E-111 | CD38+HLADR+ | SLC25A46 |
| BCKDHA | 1.69E-113 | 0.317495482 | | 0.402 | 0.102 | 4.11E-109 | CD38+HLADR+ | BCKDHA |
| GABARAPL2 | 1.44E-56 | 0.317270862 | | 0.664 | 0.295 | 3.51E-52 | CD38+HLADR+ | GABARAPL2 |
| SNRPD3 | 1.02E-60 | 0.317247535 | | 0.639 | 0.273 | 2.48E-56 | CD38+HLADR+ | SNRPD3 |
| NFATC1 | 3.33E-73 | 0.316972278 | | 0.444 | 0.153 | 8.12E-69 | CD38+HLADR+ | NFATC1 |
| MRTFA | 6.40E-77 | 0.316608642 | | 0.456 | 0.154 | 1.56E-72 | CD38+HLADR+ | MRTFA |
| BATF | 2.17E-65 | 0.316552617 | | 0.475 | 0.178 | 5.28E-61 | CD38+HLADR+ | BATF |
| ABCC12 | 2.87E-46 | 0.316356388 | | 0.719 | 0.369 | 6.99E-42 | CD38+HLADR+ | ABCC1 |
| EIF3K | 6.78E-38 | 0.315922927 | | 0.967 | 0.842 | 1.65E-33 | CD38+HLADR+ | EIF3K |
| SMCHD1 | 4.08E-22 | 0.31571992 | | 0.952 | 0.793 | 9.93E-18 | CD38+HLADR+ | SMCHD1 |
| CLINT1 | 2.74E-66 | 0.314826309 | | 0.529 | 0.203 | 6.67E-62 | CD38+HLADR+ | CLINT1 |
| GLRX3 | 2.23E-113 | 0.314796908 | | 0.406 | 0.103 | 5.42E-109 | CD38+HLADR+ | GLRX3 |
| FURIN | 1.13E-102 | 0.314414494 | | 0.301 | 0.069 | 2.74E-98 | CD38+HLADR+ | FURIN |
| HNRNPK | 3.82E-34 | 0.314046966 | | 0.924 | 0.647 | 9.30E-30 | CD38+HLADR+ | HNRNPK |
| TPR | 3.53E-45 | 0.313738525 | | 0.691 | 0.34 | 8.60E-41 | CD38+HLADR+ | TPR |
| PRKAR1A | 3.41E-64 | 0.313023827 | | 0.591 | 0.238 | 8.31E-60 | CD38+HLADR+ | PRKAR1A |
| KLF3 | 1.40E-16 | 0.312929035 | | 0.779 | 0.593 | 3.40E-12 | CD38+HLADR+ | KLF3 |
| PSENEN | 3.53E-115 | 0.312697358 | | 0.426 | 0.11 | 8.61E-111 | CD38+HLADR+ | PSENEN |
| ZC3H12D | 3.12E-84 | 0.31228892 | | 0.321 | 0.088 | 7.60E-80 | CD38+HLADR+ | ZC3H12D |
| CISD3 | 1.06E-147 | 0.312040576 | | 0.364 | 0.072 | 2.59E-143 | CD38+HLADR+ | CISD3 |
| MRPL11 | 1.23E-87 | 0.311791297 | | 0.525 | 0.173 | 3.00E-83 | CD38+HLADR+ | MRPL11 |
| ZNF292 | 2.52E-33 | 0.311648037 | | 0.742 | 0.415 | 6.15E-29 | CD38+HLADR+ | ZNF292 |
| POLR2E | 1.50E-67 | 0.311375799 | | 0.572 | 0.222 | 3.65E-63 | CD38+HLADR+ | POLR2E |
| TOMM22 | 1.66E-53 | 0.311261181 | | 0.684 | 0.313 | 4.05E-49 | CD38+HLADR+ | TOMM22 |
| APRT | 3.89E-32 | 0.310903909 | | 0.894 | 0.605 | 9.48E-28 | CD38+HLADR+ | APRT |
| UBE2D2 | 4.11E-31 | 0.310536611 | | 0.912 | 0.693 | 1.00E-26 | CD38+HLADR+ | UBE2D2 |
| PDLIM2 | 2.46E-100 | 0.310303601 | | 0.42 | 0.118 | 5.99E-96 | CD38+HLADR+ | PDLIM2 |
| SEC11C | 2.50E-63 | 0.309402415 | | 0.61 | 0.248 | 6.08E-59 | CD38+HLADR+ | SEC11C |
| TEX264 | 1.44E-82 | 0.309114955 | | 0.501 | 0.169 | 3.51E-78 | CD38+HLADR+ | TEX264 |
| H2AFJ | 8.89E-52 | 0.308674481 | | 0.666 | 0.303 | 2.17E-47 | CD38+HLADR+ | H2AFJ |
| FLII | 1.44E-114 | 0.308332398 | | 0.368 | 0.088 | 3.51E-110 | CD38+HLADR+ | FLII |
| SRSF9 | 1.33E-40 | 0.308264931 | | 0.741 | 0.391 | 3.24E-36 | CD38+HLADR+ | SRSF9 |
| TMEM183A | 1.09E-64 | 0.307944274 | | 0.556 | 0.219 | 2.64E-60 | CD38+HLADR+ | TMEM183A |
| STOML2 | 3.38E-91 | 0.307551106 | | 0.503 | 0.159 | 8.23E-87 | CD38+HLADR+ | STOML2 |
| FOXO1 | 2.22E-33 | 0.306594721 | | 0.777 | 0.448 | 5.41E-29 | CD38+HLADR+ | FOXO1 |
| ALDOA | 7.26E-29 | 0.306264205 | | 0.925 | 0.689 | 1.77E-24 | CD38+HLADR+ | ALDOA |
| PVT1 | 9.10E-79 | 0.306224864 | | 0.552 | 0.197 | 2.22E-74 | CD38+HLADR+ | PVT1 |
| SERPINB1 | 1.18E-40 | 0.306132432 | | 0.715 | 0.377 | 2.87E-36 | CD38+HLADR+ | SERPINB1 |
| SIK31 | 4.46E-35 | 0.30580244 | | 0.747 | 0.429 | 1.09E-30 | CD38+HLADR+ | SIK3 |
| DLG1 | 7.56E-74 | 0.305711794 | | 0.459 | 0.159 | 1.84E-69 | CD38+HLADR+ | DLG1 |
| BACH1 | 6.41E-87 | 0.305562735 | | 0.401 | 0.12 | 1.56E-82 | CD38+HLADR+ | BACH1 |
| ARF3 | 3.28E-137 | 0.305185686 | | 0.36 | 0.075 | 7.98E-133 | CD38+HLADR+ | ARF3 |
| DERL11 | 6.19E-79 | 0.305110496 | | 0.541 | 0.191 | 1.51E-74 | CD38+HLADR+ | DERL1 |
| IDH3G | 8.70E-93 | 0.304983446 | | 0.501 | 0.158 | 2.12E-88 | CD38+HLADR+ | IDH3G |
| OVCA2 | 1.90E-98 | 0.304966511 | | 0.465 | 0.137 | 4.63E-94 | CD38+HLADR+ | OVCA2 |
| RAB8B1 | 1.25E-55 | 0.304857598 | | 0.583 | 0.251 | 3.05E-51 | CD38+HLADR+ | RAB8B |
| CDV3 | 4.20E-34 | 0.303865233 | | 0.829 | 0.48 | 1.02E-29 | CD38+HLADR+ | CDV3 |
| RNF181 | 2.78E-63 | 0.303819377 | | 0.631 | 0.259 | 6.77E-59 | CD38+HLADR+ | RNF181 |
| GLRX5 | 2.85E-77 | 0.303768341 | | 0.54 | 0.192 | 6.95E-73 | CD38+HLADR+ | GLRX5 |
| ERH | 6.39E-34 | 0.303723723 | | 0.802 | 0.471 | 1.56E-29 | CD38+HLADR+ | ERH |
| COMTD1 | 3.62E-121 | 0.30364513 | | 0.378 | 0.088 | 8.82E-117 | CD38+HLADR+ | COMTD1 |
| BABAM1 | 5.54E-77 | 0.303461998 | | 0.48 | 0.165 | 1.35E-72 | CD38+HLADR+ | BABAM1 |
| RUNX2 | 2.03E-59 | 0.302134638 | | 0.354 | 0.124 | 4.93E-55 | CD38+HLADR+ | RUNX2 |
| BLOC1S2 | 6.87E-61 | 0.302108859 | | 0.61 | 0.252 | 1.67E-56 | CD38+HLADR+ | BLOC1S2 |
| ITGB1BP1 | 8.47E-93 | 0.301978517 | | 0.471 | 0.145 | 2.06E-88 | CD38+HLADR+ | ITGB1BP1 |
| DUT | 1.42E-53 | 0.301531305 | | 0.535 | 0.227 | 3.47E-49 | CD38+HLADR+ | DUT |
| BIRC6 | 2.39E-48 | 0.301286905 | | 0.682 | 0.324 | 5.83E-44 | CD38+HLADR+ | BIRC6 |
| AP1S2 | 2.26E-99 | 0.300501899 | | 0.413 | 0.115 | 5.50E-95 | CD38+HLADR+ | AP1S2 |
| MYL12B | 2.82E-35 | 0.300474079 | | 0.967 | 0.814 | 6.87E-31 | CD38+HLADR+ | MYL12B |
| ADH5 | 1.34E-69 | 0.300471836 | | 0.553 | 0.209 | 3.27E-65 | CD38+HLADR+ | ADH5 |
| UBE2L3 | 5.02E-40 | 0.29994593 | | 0.726 | 0.38 | 1.22E-35 | CD38+HLADR+ | UBE2L3 |
| PTPN18 | 1.81E-94 | 0.299800774 | | 0.416 | 0.119 | 4.40E-90 | CD38+HLADR+ | PTPN18 |
| ANKRD17 | 2.29E-75 | 0.299485948 | | 0.495 | 0.174 | 5.57E-71 | CD38+HLADR+ | ANKRD17 |
| ATP5ME | 4.71E-34 | 0.299131828 | | 0.791 | 0.445 | 1.15E-29 | CD38+HLADR+ | ATP5ME |
| USP3 | 3.86E-25 | 0.299109944 | | 0.841 | 0.562 | 9.41E-21 | CD38+HLADR+ | USP3 |
| HIKESHI | 3.71E-121 | 0.298920029 | | 0.364 | 0.083 | 9.03E-117 | CD38+HLADR+ | HIKESHI |
| BST2 | 1.98E-55 | 0.298791046 | | 0.513 | 0.208 | 4.81E-51 | CD38+HLADR+ | BST2 |
| UTRN1 | 1.68E-38 | 0.29831971 | | 0.763 | 0.414 | 4.09E-34 | CD38+HLADR+ | UTRN |
| FMNL11 | 6.68E-49 | 0.29831369 | | 0.723 | 0.351 | 1.63E-44 | CD38+HLADR+ | FMNL1 |
| PDCD6 | 7.50E-78 | 0.297821676 | | 0.533 | 0.189 | 1.83E-73 | CD38+HLADR+ | PDCD6 |
| ARHGEF6 | 1.13E-90 | 0.297729498 | | 0.401 | 0.116 | 2.75E-86 | CD38+HLADR+ | ARHGEF6 |
| GLUD1 | 1.73E-55 | 0.297580518 | | 0.632 | 0.278 | 4.21E-51 | CD38+HLADR+ | GLUD1 |
| KDM5B | 1.28E-75 | 0.297556932 | | 0.346 | 0.104 | 3.13E-71 | CD38+HLADR+ | KDM5B |
| YWHAE | 6.39E-60 | 0.297467233 | | 0.574 | 0.235 | 1.56E-55 | CD38+HLADR+ | YWHAE |
| ZDHHC24 | 1.58E-88 | 0.297241839 | | 0.386 | 0.111 | 3.86E-84 | CD38+HLADR+ | ZDHHC24 |
| SPG21 | 1.47E-98 | 0.296503076 | | 0.46 | 0.134 | 3.58E-94 | CD38+HLADR+ | SPG21 |
| MIS18BP1 | 8.71E-82 | 0.296470355 | | 0.41 | 0.128 | 2.12E-77 | CD38+HLADR+ | MIS18BP1 |
| SNX17 | 2.48E-59 | 0.296197988 | | 0.548 | 0.223 | 6.05E-55 | CD38+HLADR+ | SNX17 |
| JOSD2 | 3.60E-122 | 0.296066422 | | 0.344 | 0.075 | 8.77E-118 | CD38+HLADR+ | JOSD2 |
| MAZ1 | 1.08E-53 | 0.296051518 | | 0.642 | 0.287 | 2.63E-49 | CD38+HLADR+ | MAZ |
| PSMD4 | 4.55E-64 | 0.295928526 | | 0.566 | 0.223 | 1.11E-59 | CD38+HLADR+ | PSMD4 |
| SYNGR2 | 9.20E-57 | 0.295508167 | | 0.618 | 0.263 | 2.24E-52 | CD38+HLADR+ | SYNGR2 |
| CAPZA2 | 1.50E-53 | 0.295190322 | | 0.614 | 0.265 | 3.65E-49 | CD38+HLADR+ | CAPZA2 |
| NCOR1 | 2.30E-46 | 0.294870246 | | 0.739 | 0.36 | 5.60E-42 | CD38+HLADR+ | NCOR1 |
| FIS1 | 2.92E-35 | 0.294619027 | | 0.731 | 0.399 | 7.10E-31 | CD38+HLADR+ | FIS1 |
| MYO5A | 1.00E-61 | 0.294548924 | | 0.337 | 0.113 | 2.44E-57 | CD38+HLADR+ | MYO5A |
| GUK12 | 1.18E-30 | 0.294054322 | | 0.924 | 0.672 | 2.88E-26 | CD38+HLADR+ | GUK1 |
| TBC1D1 | 8.35E-80 | 0.29400849 | | 0.422 | 0.134 | 2.03E-75 | CD38+HLADR+ | TBC1D1 |
| ATP2C1 | 6.11E-97 | 0.293865418 | | 0.33 | 0.083 | 1.49E-92 | CD38+HLADR+ | ATP2C1 |
| NAA38 | 1.50E-71 | 0.293673202 | | 0.599 | 0.227 | 3.66E-67 | CD38+HLADR+ | NAA38 |
| RNASEK1 | 3.36E-25 | 0.293451215 | | 0.93 | 0.683 | 8.18E-21 | CD38+HLADR+ | RNASEK |
| SDHD | 4.43E-62 | 0.29327274 | | 0.574 | 0.227 | 1.08E-57 | CD38+HLADR+ | SDHD |
| HLA-DMA | 5.58E-73 | 0.293092362 | | 0.307 | 0.09 | 1.36E-68 | CD38+HLADR+ | HLA-DMA |
| FAM102B | 4.32E-67 | 0.292938727 | | 0.332 | 0.105 | 1.05E-62 | CD38+HLADR+ | FAM102B |
| KDM6A | 1.07E-53 | 0.292699772 | | 0.602 | 0.263 | 2.60E-49 | CD38+HLADR+ | KDM6A |
| TPGS1 | 4.01E-80 | 0.292647666 | | 0.495 | 0.167 | 9.77E-76 | CD38+HLADR+ | TPGS1 |
| STMP1 | 5.53E-61 | 0.292532044 | | 0.57 | 0.229 | 1.35E-56 | CD38+HLADR+ | STMP1 |
| OSTC | 5.04E-52 | 0.292478155 | | 0.615 | 0.271 | 1.23E-47 | CD38+HLADR+ | OSTC |
| UQCR11 | 4.97E-28 | 0.292460869 | | 0.941 | 0.732 | 1.21E-23 | CD38+HLADR+ | UQCR11 |
| PHB21 | 2.30E-32 | 0.292443852 | | 0.802 | 0.484 | 5.60E-28 | CD38+HLADR+ | PHB2 |
| COPS3 | 2.80E-78 | 0.291609456 | | 0.509 | 0.175 | 6.83E-74 | CD38+HLADR+ | COPS3 |
| ZNF524 | 1.54E-104 | 0.291499358 | | 0.354 | 0.088 | 3.74E-100 | CD38+HLADR+ | ZNF524 |
| TESC | 2.22E-155 | 0.291454275 | | 0.279 | 0.044 | 5.41E-151 | CD38+HLADR+ | TESC |
| SLMAP | 2.11E-80 | 0.291291325 | | 0.416 | 0.132 | 5.14E-76 | CD38+HLADR+ | SLMAP |
| WIPI2 | 5.53E-79 | 0.291196779 | | 0.504 | 0.173 | 1.35E-74 | CD38+HLADR+ | WIPI2 |
| R3HDM4 | 1.06E-33 | 0.290412478 | | 0.805 | 0.466 | 2.57E-29 | CD38+HLADR+ | R3HDM4 |
| GLMP | 6.56E-116 | 0.29033124 | | 0.322 | 0.071 | 1.60E-111 | CD38+HLADR+ | GLMP |
| ESYT2 | 1.74E-41 | 0.290090171 | | 0.751 | 0.393 | 4.25E-37 | CD38+HLADR+ | ESYT2 |
| KIAA1109 | 6.11E-49 | 0.28982164 | | 0.545 | 0.24 | 1.49E-44 | CD38+HLADR+ | KIAA1109 |
| MX1 | 2.61E-19 | 0.289815255 | | 0.348 | 0.191 | 6.35E-15 | CD38+HLADR+ | MX1 |
| AC010894.3 | 2.43E-84 | 0.289728872 | | 0.372 | 0.109 | 5.92E-80 | CD38+HLADR+ | AC010894.3 |
| ADAM191 | 2.70E-44 | 0.289633368 | | 0.253 | 0.089 | 6.57E-40 | CD38+HLADR+ | ADAM19 |
| VASP1 | 2.04E-78 | 0.289199803 | | 0.475 | 0.16 | 4.98E-74 | CD38+HLADR+ | VASP |
| NSD3 | 2.58E-28 | 0.289064675 | | 0.838 | 0.516 | 6.28E-24 | CD38+HLADR+ | NSD3 |
| TSC22D4 | 3.59E-70 | 0.288959455 | | 0.501 | 0.182 | 8.73E-66 | CD38+HLADR+ | TSC22D4 |
| FABP5 | 1.23E-99 | 0.288780607 | | 0.382 | 0.103 | 3.00E-95 | CD38+HLADR+ | FABP5 |
| RNF7 | 4.05E-32 | 0.288645947 | | 0.781 | 0.441 | 9.88E-28 | CD38+HLADR+ | RNF7 |
| VMP1 | 7.76E-55 | 0.288537756 | | 0.591 | 0.25 | 1.89E-50 | CD38+HLADR+ | VMP1 |
| GIMAP4 | 2.60E-46 | 0.288219263 | | 0.615 | 0.285 | 6.34E-42 | CD38+HLADR+ | GIMAP4 |
| RBBP4 | 5.14E-66 | 0.288139678 | | 0.548 | 0.211 | 1.25E-61 | CD38+HLADR+ | RBBP4 |
| ERBIN | 2.30E-70 | 0.28791402 | | 0.464 | 0.164 | 5.59E-66 | CD38+HLADR+ | ERBIN |
| MRPS11 | 4.83E-134 | 0.287854744 | | 0.338 | 0.069 | 1.18E-129 | CD38+HLADR+ | MRPS11 |
| BANF1 | 6.10E-86 | 0.287725288 | | 0.432 | 0.133 | 1.48E-81 | CD38+HLADR+ | BANF1 |
| FAM107B1 | 2.53E-28 | 0.287668865 | | 0.897 | 0.619 | 6.16E-24 | CD38+HLADR+ | FAM107B |
| MZT2B | 1.90E-27 | 0.287661532 | | 0.941 | 0.792 | 4.62E-23 | CD38+HLADR+ | MZT2B |
| EZH2 | 1.17E-121 | 0.286760583 | | 0.316 | 0.066 | 2.85E-117 | CD38+HLADR+ | EZH2 |
| CCDC6 | 5.31E-94 | 0.286758696 | | 0.393 | 0.11 | 1.29E-89 | CD38+HLADR+ | CCDC6 |
| HERC1 | 2.37E-32 | 0.286695833 | | 0.765 | 0.441 | 5.77E-28 | CD38+HLADR+ | HERC1 |
| SNX6 | 2.39E-40 | 0.286604822 | | 0.701 | 0.356 | 5.83E-36 | CD38+HLADR+ | SNX6 |
| HPS1 | 5.79E-54 | 0.286594145 | | 0.578 | 0.245 | 1.41E-49 | CD38+HLADR+ | HPS1 |
| MTCH2 | 2.64E-113 | 0.286576078 | | 0.365 | 0.087 | 6.43E-109 | CD38+HLADR+ | MTCH2 |
| RGS19 | 8.74E-85 | 0.286409219 | | 0.41 | 0.125 | 2.13E-80 | CD38+HLADR+ | RGS19 |
| AHCY | 5.20E-125 | 0.286191915 | | 0.342 | 0.074 | 1.27E-120 | CD38+HLADR+ | AHCY |
| PASK1 | 1.33E-41 | 0.285287994 | | 0.38 | 0.161 | 3.24E-37 | CD38+HLADR+ | PASK |
| SPN1 | 1.34E-68 | 0.284920637 | | 0.464 | 0.166 | 3.27E-64 | CD38+HLADR+ | SPN |
| MYO1F1 | 1.67E-65 | 0.284872969 | | 0.504 | 0.191 | 4.07E-61 | CD38+HLADR+ | MYO1F |
| TENT5C2 | 2.65E-27 | 0.284544907 | | 0.794 | 0.494 | 6.46E-23 | CD38+HLADR+ | TENT5C |
| FBXO33 | 2.17E-56 | 0.283604454 | | 0.557 | 0.231 | 5.27E-52 | CD38+HLADR+ | FBXO33 |
| MTMR14 | 5.49E-80 | 0.282840397 | | 0.465 | 0.153 | 1.34E-75 | CD38+HLADR+ | MTMR14 |
| PTAR1 | 6.14E-81 | 0.282799828 | | 0.463 | 0.152 | 1.50E-76 | CD38+HLADR+ | PTAR1 |
| HDAC7 | 5.76E-97 | 0.282631002 | | 0.368 | 0.098 | 1.40E-92 | CD38+HLADR+ | HDAC7 |
| PMVK | 1.74E-108 | 0.282568987 | | 0.329 | 0.076 | 4.23E-104 | CD38+HLADR+ | PMVK |
| HIVEP21 | 1.60E-41 | 0.282489484 | | 0.607 | 0.292 | 3.89E-37 | CD38+HLADR+ | HIVEP2 |
| MFHAS1 | 1.48E-71 | 0.282414012 | | 0.311 | 0.092 | 3.61E-67 | CD38+HLADR+ | MFHAS1 |
| PFKP | 3.19E-91 | 0.282147185 | | 0.36 | 0.099 | 7.77E-87 | CD38+HLADR+ | PFKP |
| SCAMP2 | 5.31E-60 | 0.282045343 | | 0.559 | 0.225 | 1.29E-55 | CD38+HLADR+ | SCAMP2 |
| TIMM8B | 1.75E-110 | 0.282004083 | | 0.388 | 0.097 | 4.26E-106 | CD38+HLADR+ | TIMM8B |
| C12orf751 | 4.39E-34 | 0.281863545 | | 0.472 | 0.233 | 1.07E-29 | CD38+HLADR+ | C12orf75 |
| TMOD3 | 1.12E-58 | 0.281743471 | | 0.536 | 0.217 | 2.73E-54 | CD38+HLADR+ | TMOD3 |
| GNA13 | 1.16E-51 | 0.281478136 | | 0.6 | 0.263 | 2.83E-47 | CD38+HLADR+ | GNA13 |
| AC008555.4 | 3.61E-84 | 0.281447463 | | 0.381 | 0.113 | 8.79E-80 | CD38+HLADR+ | AC008555.4 |
| RNASET21 | 2.98E-28 | 0.281080472 | | 0.832 | 0.507 | 7.26E-24 | CD38+HLADR+ | RNASET2 |
| NDUFB6 | 6.39E-64 | 0.280979844 | | 0.575 | 0.227 | 1.56E-59 | CD38+HLADR+ | NDUFB6 |
| COX16 | 1.76E-54 | 0.280761096 | | 0.632 | 0.273 | 4.29E-50 | CD38+HLADR+ | COX16 |
| SMC6 | 1.05E-128 | 0.280364178 | | 0.285 | 0.053 | 2.56E-124 | CD38+HLADR+ | SMC6 |
| TAPBP1 | 7.76E-38 | 0.2802941 | | 0.743 | 0.391 | 1.89E-33 | CD38+HLADR+ | TAPBP |
| ARID4B | 3.30E-24 | 0.280192846 | | 0.914 | 0.666 | 8.03E-20 | CD38+HLADR+ | ARID4B |
| SMC3 | 8.71E-62 | 0.2801286 | | 0.61 | 0.248 | 2.12E-57 | CD38+HLADR+ | SMC3 |
| MTPN | 3.08E-42 | 0.27987009 | | 0.683 | 0.336 | 7.50E-38 | CD38+HLADR+ | MTPN |
| SNRPG | 2.02E-29 | 0.27966893 | | 0.83 | 0.509 | 4.92E-25 | CD38+HLADR+ | SNRPG |
| FAM207A | 4.97E-125 | 0.279397069 | | 0.295 | 0.058 | 1.21E-120 | CD38+HLADR+ | FAM207A |
| STXBP2 | 6.18E-66 | 0.279047534 | | 0.519 | 0.195 | 1.50E-61 | CD38+HLADR+ | STXBP2 |
| MRPS24 | 3.54E-41 | 0.278916803 | | 0.701 | 0.351 | 8.63E-37 | CD38+HLADR+ | MRPS24 |
| CUEDC2 | 1.80E-97 | 0.278881606 | | 0.46 | 0.134 | 4.39E-93 | CD38+HLADR+ | CUEDC2 |
| MHENCR | 3.68E-52 | 0.278843111 | | 0.572 | 0.247 | 8.96E-48 | CD38+HLADR+ | MHENCR |
| AP001011.1 | 2.51E-46 | 0.278717461 | | 0.489 | 0.215 | 6.10E-42 | CD38+HLADR+ | AP001011.1 |
| CCT7 | 2.38E-54 | 0.278648036 | | 0.594 | 0.251 | 5.79E-50 | CD38+HLADR+ | CCT7 |
| UBE2J1 | 3.64E-51 | 0.278415101 | | 0.576 | 0.252 | 8.85E-47 | CD38+HLADR+ | UBE2J1 |
| CD54 | 2.74E-33 | 0.278400819 | | 0.753 | 0.434 | 6.68E-29 | CD38+HLADR+ | CD5 |
| RHOF1 | 1.10E-42 | 0.278303552 | | 0.693 | 0.348 | 2.69E-38 | CD38+HLADR+ | RHOF |
| EXOC4 | 2.18E-61 | 0.27828879 | | 0.513 | 0.201 | 5.32E-57 | CD38+HLADR+ | EXOC4 |
| JAK3 | 9.09E-72 | 0.27820561 | | 0.523 | 0.19 | 2.21E-67 | CD38+HLADR+ | JAK3 |
| AP3S1 | 2.62E-51 | 0.277997361 | | 0.513 | 0.215 | 6.38E-47 | CD38+HLADR+ | AP3S1 |
| CNST | 1.36E-46 | 0.277844315 | | 0.652 | 0.307 | 3.32E-42 | CD38+HLADR+ | CNST |
| FAM89B | 1.53E-81 | 0.277348308 | | 0.358 | 0.105 | 3.72E-77 | CD38+HLADR+ | FAM89B |
| CSGALNACT2 | 1.28E-64 | 0.27726797 | | 0.551 | 0.213 | 3.11E-60 | CD38+HLADR+ | CSGALNACT2 |
| QARS | 1.36E-78 | 0.277169608 | | 0.405 | 0.128 | 3.31E-74 | CD38+HLADR+ | QARS |
| RPL26L1 | 3.61E-116 | 0.276835743 | | 0.332 | 0.074 | 8.80E-112 | CD38+HLADR+ | RPL26L1 |
| TMEM14C | 5.82E-96 | 0.276766519 | | 0.372 | 0.1 | 1.42E-91 | CD38+HLADR+ | TMEM14C |
| IFI16 | 3.51E-47 | 0.2761115 | | 0.642 | 0.298 | 8.55E-43 | CD38+HLADR+ | IFI16 |
| LSM1 | 4.54E-73 | 0.275875541 | | 0.519 | 0.185 | 1.10E-68 | CD38+HLADR+ | LSM1 |
| HLA-A2 | 1.09E-21 | 0.275598056 | | 1 | 0.992 | 2.65E-17 | CD38+HLADR+ | HLA-A |
| AP1M1 | 3.31E-84 | 0.275250754 | | 0.393 | 0.117 | 8.06E-80 | CD38+HLADR+ | AP1M1 |
| MPST | 3.50E-128 | 0.275173624 | | 0.278 | 0.051 | 8.51E-124 | CD38+HLADR+ | MPST |
| GABARAP | 7.40E-26 | 0.27503196 | | 0.939 | 0.708 | 1.80E-21 | CD38+HLADR+ | GABARAP |
| DDHD1 | 1.41E-63 | 0.274596243 | | 0.452 | 0.166 | 3.43E-59 | CD38+HLADR+ | DDHD1 |
| HNRNPR | 2.97E-35 | 0.274361139 | | 0.699 | 0.376 | 7.23E-31 | CD38+HLADR+ | HNRNPR |
| DCAF11 | 1.29E-102 | 0.274166894 | | 0.386 | 0.102 | 3.14E-98 | CD38+HLADR+ | DCAF11 |
| ANKRD39 | 2.11E-79 | 0.274038902 | | 0.41 | 0.128 | 5.14E-75 | CD38+HLADR+ | ANKRD39 |
| ROCK1 | 2.50E-49 | 0.273945561 | | 0.684 | 0.312 | 6.08E-45 | CD38+HLADR+ | ROCK1 |
| KDM7A | 7.56E-67 | 0.273760995 | | 0.39 | 0.133 | 1.84E-62 | CD38+HLADR+ | KDM7A |
| GSTO1 | 6.33E-79 | 0.27367938 | | 0.42 | 0.134 | 1.54E-74 | CD38+HLADR+ | GSTO1 |
| MRPS34 | 1.37E-62 | 0.273217456 | | 0.559 | 0.22 | 3.33E-58 | CD38+HLADR+ | MRPS34 |
| TPGS2 | 1.70E-111 | 0.273169701 | | 0.328 | 0.074 | 4.13E-107 | CD38+HLADR+ | TPGS2 |
| HTATIP2 | 5.91E-94 | 0.273135961 | | 0.36 | 0.096 | 1.44E-89 | CD38+HLADR+ | HTATIP2 |
| CDK2AP1 | 8.62E-153 | 0.272999686 | | 0.273 | 0.042 | 2.10E-148 | CD38+HLADR+ | CDK2AP1 |
| RTN4 | 1.88E-38 | 0.272988345 | | 0.65 | 0.324 | 4.58E-34 | CD38+HLADR+ | RTN4 |
| TRAF7 | 2.68E-131 | 0.27264225 | | 0.311 | 0.061 | 6.53E-127 | CD38+HLADR+ | TRAF7 |
| PTPN11 | 5.54E-83 | 0.272599481 | | 0.439 | 0.137 | 1.35E-78 | CD38+HLADR+ | PTPN11 |
| UBE2E1 | 3.64E-63 | 0.272592725 | | 0.422 | 0.15 | 8.87E-59 | CD38+HLADR+ | UBE2E1 |
| CYC11 | 7.70E-67 | 0.272311456 | | 0.517 | 0.192 | 1.88E-62 | CD38+HLADR+ | CYC1 |
| EIF4G2 | 2.84E-28 | 0.272195862 | | 0.84 | 0.534 | 6.92E-24 | CD38+HLADR+ | EIF4G2 |
| ETF1 | 7.87E-60 | 0.271796383 | | 0.572 | 0.231 | 1.92E-55 | CD38+HLADR+ | ETF1 |
| DUSP16 | 3.22E-54 | 0.271758712 | | 0.455 | 0.18 | 7.84E-50 | CD38+HLADR+ | DUSP16 |
| WDR26 | 2.15E-50 | 0.271712771 | | 0.508 | 0.213 | 5.24E-46 | CD38+HLADR+ | WDR26 |
| HECTD1 | 1.43E-44 | 0.27164776 | | 0.599 | 0.279 | 3.49E-40 | CD38+HLADR+ | HECTD1 |
| NCOA4 | 1.01E-76 | 0.271566994 | | 0.437 | 0.143 | 2.45E-72 | CD38+HLADR+ | NCOA4 |
| ATG3 | 5.87E-74 | 0.271427657 | | 0.52 | 0.184 | 1.43E-69 | CD38+HLADR+ | ATG3 |
| NUDT2 | 1.03E-94 | 0.271420302 | | 0.378 | 0.103 | 2.50E-90 | CD38+HLADR+ | NUDT2 |
| MALT11 | 2.10E-28 | 0.27104413 | | 0.755 | 0.439 | 5.11E-24 | CD38+HLADR+ | MALT1 |
| TAOK31 | 4.59E-56 | 0.270808926 | | 0.564 | 0.236 | 1.12E-51 | CD38+HLADR+ | TAOK3 |
| SKA2 | 2.01E-129 | 0.270750154 | | 0.302 | 0.058 | 4.89E-125 | CD38+HLADR+ | SKA2 |
| RSU1 | 3.63E-81 | 0.270724689 | | 0.426 | 0.135 | 8.83E-77 | CD38+HLADR+ | RSU1 |
| MFF | 8.77E-53 | 0.270685166 | | 0.604 | 0.262 | 2.14E-48 | CD38+HLADR+ | MFF |
| DENND10 | 9.11E-74 | 0.270321171 | | 0.42 | 0.139 | 2.22E-69 | CD38+HLADR+ | DENND10 |
| ISY1 | 1.78E-79 | 0.270196391 | | 0.445 | 0.144 | 4.34E-75 | CD38+HLADR+ | ISY1 |
| NT5C3A | 1.51E-53 | 0.269750138 | | 0.606 | 0.259 | 3.69E-49 | CD38+HLADR+ | NT5C3A |
| PSMA1 | 1.93E-29 | 0.269740109 | | 0.858 | 0.543 | 4.70E-25 | CD38+HLADR+ | PSMA1 |
| COMT | 5.39E-103 | 0.269672091 | | 0.311 | 0.072 | 1.31E-98 | CD38+HLADR+ | COMT |
| PQBP1 | 3.03E-54 | 0.269639013 | | 0.626 | 0.266 | 7.37E-50 | CD38+HLADR+ | PQBP1 |
| MAN2A1 | 1.86E-55 | 0.269524517 | | 0.497 | 0.202 | 4.53E-51 | CD38+HLADR+ | MAN2A1 |
| REEP51 | 2.36E-47 | 0.269176806 | | 0.616 | 0.281 | 5.75E-43 | CD38+HLADR+ | REEP5 |
| CAPRIN1 | 7.07E-72 | 0.267928276 | | 0.477 | 0.168 | 1.72E-67 | CD38+HLADR+ | CAPRIN1 |
| MDH1 | 3.05E-46 | 0.267583984 | | 0.59 | 0.268 | 7.42E-42 | CD38+HLADR+ | MDH1 |
| UBA6 | 5.19E-78 | 0.267534131 | | 0.436 | 0.142 | 1.27E-73 | CD38+HLADR+ | UBA6 |
| DIAPH11 | 1.62E-52 | 0.267507608 | | 0.535 | 0.226 | 3.94E-48 | CD38+HLADR+ | DIAPH1 |
| CCT8 | 1.45E-41 | 0.267470981 | | 0.705 | 0.346 | 3.53E-37 | CD38+HLADR+ | CCT8 |
| NRDC | 6.20E-83 | 0.267460526 | | 0.456 | 0.145 | 1.51E-78 | CD38+HLADR+ | NRDC |
| UPF3A | 1.22E-71 | 0.267347896 | | 0.441 | 0.151 | 2.98E-67 | CD38+HLADR+ | UPF3A |
| GRHPR | 2.08E-97 | 0.267181143 | | 0.422 | 0.119 | 5.06E-93 | CD38+HLADR+ | GRHPR |
| N4BP1 | 3.99E-47 | 0.26712297 | | 0.603 | 0.271 | 9.72E-43 | CD38+HLADR+ | N4BP1 |
| CAT | 2.89E-123 | 0.2670518 | | 0.318 | 0.066 | 7.04E-119 | CD38+HLADR+ | CAT |
| CLIP1 | 8.03E-66 | 0.266970757 | | 0.404 | 0.139 | 1.95E-61 | CD38+HLADR+ | CLIP1 |
| HIST1H1D | 6.91E-43 | 0.26689976 | | 0.532 | 0.244 | 1.68E-38 | CD38+HLADR+ | HIST1H1D |
| GTF2H5 | 5.47E-113 | 0.266749542 | | 0.31 | 0.067 | 1.33E-108 | CD38+HLADR+ | GTF2H5 |
| NSMCE2 | 5.14E-71 | 0.266379408 | | 0.425 | 0.145 | 1.25E-66 | CD38+HLADR+ | NSMCE2 |
| RAB11FIP1 | 2.97E-67 | 0.26612591 | | 0.37 | 0.123 | 7.24E-63 | CD38+HLADR+ | RAB11FIP1 |
| ZAP70 | 2.85E-31 | 0.266107469 | | 0.809 | 0.47 | 6.94E-27 | CD38+HLADR+ | ZAP70 |
| NSF | 2.80E-58 | 0.266057334 | | 0.406 | 0.149 | 6.83E-54 | CD38+HLADR+ | NSF |
| PFKFB31 | 1.30E-56 | 0.266044947 | | 0.447 | 0.173 | 3.17E-52 | CD38+HLADR+ | PFKFB3 |
| GPS1 | 1.56E-86 | 0.26547701 | | 0.413 | 0.123 | 3.81E-82 | CD38+HLADR+ | GPS1 |
| GMDS | 7.75E-78 | 0.265426898 | | 0.436 | 0.142 | 1.89E-73 | CD38+HLADR+ | GMDS |
| RASA2 | 1.17E-26 | 0.265420213 | | 0.805 | 0.481 | 2.86E-22 | CD38+HLADR+ | RASA2 |
| RASAL31 | 1.62E-58 | 0.265400008 | | 0.532 | 0.213 | 3.94E-54 | CD38+HLADR+ | RASAL3 |
| ECHS1 | 3.24E-120 | 0.265373737 | | 0.337 | 0.073 | 7.88E-116 | CD38+HLADR+ | ECHS1 |
| COMMD8 | 3.23E-96 | 0.265351087 | | 0.35 | 0.091 | 7.86E-92 | CD38+HLADR+ | COMMD8 |
| MAN2B1 | 2.24E-86 | 0.265346125 | | 0.414 | 0.124 | 5.45E-82 | CD38+HLADR+ | MAN2B1 |
| ZFP91 | 1.06E-79 | 0.265261321 | | 0.471 | 0.155 | 2.59E-75 | CD38+HLADR+ | ZFP91 |
| ALDH16A1 | 2.15E-144 | 0.265213257 | | 0.302 | 0.053 | 5.24E-140 | CD38+HLADR+ | ALDH16A1 |
| RALBP1 | 1.33E-87 | 0.265011941 | | 0.393 | 0.114 | 3.24E-83 | CD38+HLADR+ | RALBP1 |
| GRN | 9.00E-137 | 0.264919573 | | 0.281 | 0.049 | 2.19E-132 | CD38+HLADR+ | GRN |
| GALM1 | 8.33E-60 | 0.264887075 | | 0.449 | 0.17 | 2.03E-55 | CD38+HLADR+ | GALM |
| CFLAR1 | 9.18E-35 | 0.264723429 | | 0.761 | 0.404 | 2.24E-30 | CD38+HLADR+ | CFLAR |
| SMC4 | 2.37E-70 | 0.264546106 | | 0.365 | 0.117 | 5.78E-66 | CD38+HLADR+ | SMC4 |
| CCT6A | 2.97E-57 | 0.264541837 | | 0.528 | 0.211 | 7.22E-53 | CD38+HLADR+ | CCT6A |
| SH3GLB11 | 1.03E-54 | 0.264464326 | | 0.587 | 0.247 | 2.52E-50 | CD38+HLADR+ | SH3GLB1 |
| CYBC1 | 7.45E-78 | 0.26440361 | | 0.385 | 0.118 | 1.82E-73 | CD38+HLADR+ | CYBC1 |
| EMP3 | 1.93E-24 | 0.264098624 | | 0.952 | 0.728 | 4.69E-20 | CD38+HLADR+ | EMP3 |
| RPUSD3 | 4.87E-170 | 0.263949465 | | 0.265 | 0.036 | 1.19E-165 | CD38+HLADR+ | RPUSD3 |
| SPAG7 | 5.11E-75 | 0.263873413 | | 0.493 | 0.171 | 1.24E-70 | CD38+HLADR+ | SPAG7 |
| SNRPD1 | 7.00E-38 | 0.26376089 | | 0.75 | 0.381 | 1.70E-33 | CD38+HLADR+ | SNRPD1 |
| SDF4 | 2.41E-48 | 0.263579175 | | 0.587 | 0.261 | 5.86E-44 | CD38+HLADR+ | SDF4 |
| ACAA2 | 2.94E-83 | 0.263571666 | | 0.397 | 0.119 | 7.17E-79 | CD38+HLADR+ | ACAA2 |
| ATP2B1 | 1.71E-34 | 0.263556012 | | 0.598 | 0.308 | 4.15E-30 | CD38+HLADR+ | ATP2B1 |
| PPP6C | 1.15E-45 | 0.263382073 | | 0.664 | 0.309 | 2.81E-41 | CD38+HLADR+ | PPP6C |
| PICALM1 | 3.00E-61 | 0.263365875 | | 0.566 | 0.226 | 7.30E-57 | CD38+HLADR+ | PICALM |
| MPV17 | 3.92E-126 | 0.262918456 | | 0.306 | 0.061 | 9.54E-122 | CD38+HLADR+ | MPV17 |
| NUDT1 | 2.73E-87 | 0.262550655 | | 0.373 | 0.106 | 6.64E-83 | CD38+HLADR+ | NUDT1 |
| CCDC69 | 5.71E-59 | 0.262473544 | | 0.549 | 0.222 | 1.39E-54 | CD38+HLADR+ | CCDC69 |
| GOLGA7 | 2.07E-45 | 0.262221245 | | 0.663 | 0.306 | 5.03E-41 | CD38+HLADR+ | GOLGA7 |
| CMC2 | 1.34E-50 | 0.26199347 | | 0.606 | 0.267 | 3.26E-46 | CD38+HLADR+ | CMC2 |
| MPDU1 | 1.43E-118 | 0.26179954 | | 0.329 | 0.071 | 3.47E-114 | CD38+HLADR+ | MPDU1 |
| CNPY2 | 1.56E-66 | 0.261696298 | | 0.433 | 0.153 | 3.80E-62 | CD38+HLADR+ | CNPY2 |
| SPOCK22 | 2.69E-24 | 0.261344868 | | 0.909 | 0.682 | 6.55E-20 | CD38+HLADR+ | SPOCK2 |
| IQGAP1 | 3.34E-32 | 0.261255115 | | 0.735 | 0.399 | 8.12E-28 | CD38+HLADR+ | IQGAP1 |
| TNFSF10 | 5.43E-81 | 0.260926538 | | 0.307 | 0.083 | 1.32E-76 | CD38+HLADR+ | TNFSF10 |
| ABCG1 | 4.35E-62 | 0.260611883 | | 0.385 | 0.134 | 1.06E-57 | CD38+HLADR+ | ABCG1 |
| SF3B6 | 4.12E-30 | 0.260558609 | | 0.798 | 0.454 | 1.00E-25 | CD38+HLADR+ | SF3B6 |
| BCL11B | 6.29E-22 | 0.260296867 | | 0.854 | 0.573 | 1.53E-17 | CD38+HLADR+ | BCL11B |
| POR | 3.20E-50 | 0.260189859 | | 0.356 | 0.134 | 7.80E-46 | CD38+HLADR+ | POR |
| GNG10 | 3.15E-84 | 0.259961656 | | 0.398 | 0.119 | 7.66E-80 | CD38+HLADR+ | GNG10 |
| IL6ST1 | 1.65E-30 | 0.259920606 | | 0.73 | 0.407 | 4.02E-26 | CD38+HLADR+ | IL6ST |
| CAMK2D1 | 7.10E-50 | 0.259919809 | | 0.516 | 0.222 | 1.73E-45 | CD38+HLADR+ | CAMK2D |
| MRPS12 | 2.02E-86 | 0.259873873 | | 0.4 | 0.118 | 4.91E-82 | CD38+HLADR+ | MRPS12 |
| CIAO2B | 7.13E-37 | 0.259762071 | | 0.723 | 0.373 | 1.74E-32 | CD38+HLADR+ | CIAO2B |
| SMARCB1 | 1.94E-71 | 0.259369775 | | 0.481 | 0.168 | 4.72E-67 | CD38+HLADR+ | SMARCB1 |
| ATP5MC1 | 2.49E-74 | 0.259264265 | | 0.473 | 0.162 | 6.06E-70 | CD38+HLADR+ | ATP5MC1 |
| HNRNPD | 2.67E-27 | 0.259055544 | | 0.845 | 0.518 | 6.51E-23 | CD38+HLADR+ | HNRNPD |
| RBM4 | 6.39E-52 | 0.258950192 | | 0.644 | 0.282 | 1.56E-47 | CD38+HLADR+ | RBM4 |
| NAPG | 6.10E-78 | 0.258884277 | | 0.384 | 0.118 | 1.49E-73 | CD38+HLADR+ | NAPG |
| IFITM3 | 1.49E-46 | 0.258798999 | | 0.299 | 0.111 | 3.64E-42 | CD38+HLADR+ | IFITM3 |
| DMAC1 | 9.20E-94 | 0.258619504 | | 0.332 | 0.085 | 2.24E-89 | CD38+HLADR+ | DMAC1 |
| CLPTM1L | 9.16E-96 | 0.258297687 | | 0.33 | 0.083 | 2.23E-91 | CD38+HLADR+ | CLPTM1L |
| TAX1BP1 | 1.48E-28 | 0.258097582 | | 0.807 | 0.483 | 3.61E-24 | CD38+HLADR+ | TAX1BP1 |
| TRIM59 | 5.87E-76 | 0.25798054 | | 0.277 | 0.073 | 1.43E-71 | CD38+HLADR+ | TRIM59 |
| LRMP | 2.57E-82 | 0.257410708 | | 0.325 | 0.089 | 6.26E-78 | CD38+HLADR+ | LRMP |
| CBX3 | 1.75E-35 | 0.257277269 | | 0.718 | 0.377 | 4.25E-31 | CD38+HLADR+ | CBX3 |
| ZNF106 | 3.86E-67 | 0.257172841 | | 0.362 | 0.119 | 9.39E-63 | CD38+HLADR+ | ZNF106 |
| IRF9 | 3.26E-56 | 0.257143281 | | 0.568 | 0.238 | 7.93E-52 | CD38+HLADR+ | IRF9 |
| CASP8 | 2.60E-29 | 0.257069482 | | 0.763 | 0.443 | 6.33E-25 | CD38+HLADR+ | CASP8 |
| NECAP2 | 3.33E-46 | 0.256741579 | | 0.623 | 0.289 | 8.12E-42 | CD38+HLADR+ | NECAP2 |
| NHP2 | 8.95E-48 | 0.25619012 | | 0.549 | 0.241 | 2.18E-43 | CD38+HLADR+ | NHP2 |
| PLAAT41 | 2.29E-21 | 0.256124286 | | 0.836 | 0.568 | 5.57E-17 | CD38+HLADR+ | PLAAT4 |
| DENND1C | 1.78E-62 | 0.255918239 | | 0.545 | 0.211 | 4.33E-58 | CD38+HLADR+ | DENND1C |
| POLE4 | 4.83E-54 | 0.255670156 | | 0.529 | 0.222 | 1.18E-49 | CD38+HLADR+ | POLE4 |
| UBA1 | 5.28E-86 | 0.255404526 | | 0.349 | 0.097 | 1.28E-81 | CD38+HLADR+ | UBA1 |
| CSNK2A1 | 1.11E-75 | 0.254958245 | | 0.443 | 0.146 | 2.70E-71 | CD38+HLADR+ | CSNK2A1 |
| SCFD1 | 1.82E-55 | 0.254852485 | | 0.473 | 0.186 | 4.43E-51 | CD38+HLADR+ | SCFD1 |
| RFXANK | 1.56E-86 | 0.254685632 | | 0.374 | 0.107 | 3.79E-82 | CD38+HLADR+ | RFXANK |
| AKR7A2 | 1.88E-110 | 0.254542976 | | 0.314 | 0.069 | 4.57E-106 | CD38+HLADR+ | AKR7A2 |
| UBE2D3 | 1.07E-25 | 0.25445451 | | 0.967 | 0.815 | 2.60E-21 | CD38+HLADR+ | UBE2D3 |
| NDUFS8 | 4.42E-47 | 0.254395873 | | 0.663 | 0.304 | 1.08E-42 | CD38+HLADR+ | NDUFS8 |
| TOR3A | 1.32E-101 | 0.254389995 | | 0.336 | 0.082 | 3.21E-97 | CD38+HLADR+ | TOR3A |
| CAB39 | 4.51E-53 | 0.254151907 | | 0.449 | 0.177 | 1.10E-48 | CD38+HLADR+ | CAB39 |
| SLC39A7 | 6.41E-118 | 0.253959043 | | 0.291 | 0.059 | 1.56E-113 | CD38+HLADR+ | SLC39A7 |
| ILK | 7.01E-87 | 0.253669156 | | 0.33 | 0.088 | 1.71E-82 | CD38+HLADR+ | ILK |
| C11orf98 | 4.60E-89 | 0.253625482 | | 0.356 | 0.097 | 1.12E-84 | CD38+HLADR+ | C11orf98 |
| PAFAH1B11 | 1.82E-32 | 0.253394582 | | 0.727 | 0.396 | 4.42E-28 | CD38+HLADR+ | PAFAH1B1 |
| ANKRD10 | 2.85E-65 | 0.253168536 | | 0.523 | 0.196 | 6.93E-61 | CD38+HLADR+ | ANKRD10 |
| HDAC4 | 1.79E-65 | 0.253041686 | | 0.358 | 0.118 | 4.37E-61 | CD38+HLADR+ | HDAC4 |
| SLC35D2 | 2.52E-67 | 0.252259835 | | 0.386 | 0.129 | 6.13E-63 | CD38+HLADR+ | SLC35D2 |
| ZNF580 | 3.14E-84 | 0.252162093 | | 0.434 | 0.134 | 7.66E-80 | CD38+HLADR+ | ZNF580 |
| FAM120A | 7.60E-69 | 0.252023225 | | 0.409 | 0.138 | 1.85E-64 | CD38+HLADR+ | FAM120A |
| TRADD2 | 3.39E-51 | 0.251783259 | | 0.603 | 0.266 | 8.25E-47 | CD38+HLADR+ | TRADD |
| ADI1 | 1.44E-94 | 0.251592593 | | 0.341 | 0.088 | 3.52E-90 | CD38+HLADR+ | ADI1 |
| MRPL48 | 7.25E-105 | 0.251570347 | | 0.286 | 0.062 | 1.76E-100 | CD38+HLADR+ | MRPL48 |
| ATP6V0E2 | 2.77E-70 | 0.251474624 | | 0.477 | 0.167 | 6.75E-66 | CD38+HLADR+ | ATP6V0E2 |
| EIF3H | 2.83E-25 | 0.251402704 | | 0.933 | 0.759 | 6.90E-21 | CD38+HLADR+ | EIF3H |
| CISD2 | 6.58E-72 | 0.251380421 | | 0.452 | 0.155 | 1.60E-67 | CD38+HLADR+ | CISD2 |
| GIT2 | 2.87E-65 | 0.251237214 | | 0.459 | 0.164 | 7.00E-61 | CD38+HLADR+ | GIT2 |
| HCFC1R1 | 1.27E-91 | 0.251089831 | | 0.368 | 0.1 | 3.10E-87 | CD38+HLADR+ | HCFC1R1 |
| NFAT51 | 1.03E-52 | 0.251020761 | | 0.463 | 0.187 | 2.50E-48 | CD38+HLADR+ | NFAT5 |
| C1orf43 | 1.35E-32 | 0.250926735 | | 0.75 | 0.406 | 3.30E-28 | CD38+HLADR+ | C1orf43 |
| SNRPB | 2.63E-24 | 0.250779438 | | 0.836 | 0.532 | 6.40E-20 | CD38+HLADR+ | SNRPB |
| LRRC8B | 1.75E-103 | 0.250651452 | | 0.265 | 0.055 | 4.27E-99 | CD38+HLADR+ | LRRC8B |
| RPIA | 1.72E-79 | 0.250597434 | | 0.459 | 0.15 | 4.19E-75 | CD38+HLADR+ | RPIA |
| SERBP1 | 2.03E-25 | 0.250589479 | | 0.854 | 0.561 | 4.95E-21 | CD38+HLADR+ | SERBP1 |
| KPNA3 | 2.57E-74 | 0.250402216 | | 0.416 | 0.135 | 6.25E-70 | CD38+HLADR+ | KPNA3 |
| CAST | 8.78E-25 | 0.250165327 | | 0.762 | 0.455 | 2.14E-20 | CD38+HLADR+ | CAST |
| EIF2AK1 | 5.02E-70 | 0.250128998 | | 0.432 | 0.147 | 1.22E-65 | CD38+HLADR+ | EIF2AK1 |
| FAM32A | 1.29E-54 | 0.250093525 | | 0.552 | 0.229 | 3.14E-50 | CD38+HLADR+ | FAM32A |

**Supplementary Table 7. Lists of genes used to calculate functional scores for**

**CD38^+^HLA-DR^+^ T cells.**

| **T cell exhaustion** | **T cell Effector** | **Cytokine** |
| --- | --- | --- |
| PDCD1 | ABCA2 | IL2 |
| TIGIT | ACP5 | IL7 |
| LAG3 | AK3 | CSF3 |
| CTLA4 | ANAPC16 | CXCL10 |
| HAVCR2 | ANAPC5 | CCL2 |
| BTLA | ANXA1 | CCL3 |
| CD80 | ANXA6 | TNF |
| KLRG1 | AP1M1 | IL6 |
| CD200 | ARFRP1 | CCL7 |
| CD244 | ARL4C | IL1RN |
|  | ATP5PO | CSF1 |
|  | ATP6V0B | IFNG |
|  | ATP6V0C | IL2RA |
|  | AURKAIP1 | IL10 |
|  | AXIN1 | IL18 |
|  | B4GALT1 | HGF |
|  | BCKDK | CXCL9 |
|  | BLVRA | CCL27 |
|  | BNIP3L | TGFB1 |
|  | BRAP | IL1B |
|  | BSCL2 | LTA |
|  | C8orf33 | CSF2 |
|  | CAB39L | LTB |
|  | CALU | TNFSF13 |
|  | CCNDBP1 | IL4 |
|  | CCR2 | CCL12 |
|  | CDC37 | CXCL8 |
|  | CDKN2D | CXCL11 |
|  | CHFR | CCL4 |
|  | CIB1 | CXCL1 |
|  | CMAS | CXCL2 |
|  | CMTM7 | CXCL3 |
|  | COMMD7 | CCL3L1 |
|  | CORO1B | CCL8 |
|  | CPT2 | CXCL16 |
|  | CSRP3 | IFNA1 |
|  | CTSA | CCL5 |
|  | CTSD | CCL11 |
|  | DAP | IFNA2 |
|  | DBI | CCL20 |
|  | DCPS | CCL4L2 |
|  | DCTN5 | OSM |
|  | DDX41 | TNFSF14 |
|  | DEAF1 | SA100A12 |
|  | DGKA | FGF19 |
|  | DHRS1 | CXCL5 |
|  | DNAJB1 | CCL19 |
|  | DNM1 | IL18R1 |
|  | DPEP1 | TGFA |
|  | DPM2 | IFNB1 |
|  | DUS1L | IL8 |
|  | DYM | IL17C |
|  | EBNA1BP2 | TNFSF10 |
|  | EFTUD2 | FGF7 |
|  | EIF2B4 | XCL1 |
|  | EIF2B5 | FGF13 |
|  | EIF2S1 | LIF |
|  | EIF3B | TGFB3 |
|  | EIF3L | INHBE |
|  | EIF4A3 | CERS1 |
|  | EIF6 | TXLNA |
|  | ELAVL1 | IFNW1 |
|  | ENTPD4 | IL22 |
|  | ETS1 | XCL2 |
|  | FAM117A | CCL25 |
|  | FAM89B | CCL16 |
|  | FCGR2B | CD40LG |
|  | FEZ2 | IL20 |
|  | FGFR3 | FASLG |
|  | FHL2 | TPO |
|  | GCAT | SCYL3 |
|  | GDAP2 | PF4V1 |
|  | GIT1 | TNFSF8 |
|  | GLIPR2 | GDF15 |
|  | GOLM1 | IL1A |
|  | GPC1 | VEGFA |
|  | GRAMD2B | GDF7 |
|  | HADHB | BMP6 |
|  | HCLS1 | PDGFA |
|  | HERPUD1 | IL21 |
|  | HIKESHI | ABCD-1 |
|  | HIPK1 | ABCD-2 |
|  | HMCES | PDGFB |
|  | HNRNPAB | TNFSF4 |
|  | HSD11B1 | FAM19A1 |
|  | ICAM2 | HBEGF |
|  | IFITM10 | PDGFD |
|  | IL17RA | IL12RB2 |
|  | IL18RAP | GH1 |
|  | ISYNA1 | VEGFB |
|  | ITGAL | MIP3B |
|  | ITGAX | IL27 |
|  | ITGB7 | PF4 |
|  | JKAMP | BMP8B |
|  | KCNJ8 | TNFSF12 |
|  | KIAA2013 | IL15 |
|  | KLK8 | SCYL2 |
|  | KLRC1 | SCYL1 |
|  | KLRD1 | TSLP |
|  | KLRG1 | GDF11 |
|  | KLRK1 | SDF1B |
|  | LAMTOR4 | INHBA |
|  | LAMTOR5 | PPBP |
|  | LDAH | FGF11 |
|  | LEF1 | IFNG-AS1 |
|  | LGALS9B | FGF22 |
|  | LRWD1 | VEGFC |
|  | LSM1 | CCL18 |
|  | LSM4 | TNFSF11 |
|  | LY6H | IL12A |
|  | LYSMD1 | EBI3 |
|  | MBP | AMH |
|  | MFNG | IL26 |
|  | MIEN1 | IL32 |
|  | MKNK2 | PDGFC |
|  | MLX | FGF23 |
|  | MRPL34 | IGF1 |
|  | MTCH1 | IL1F11 |
|  | MTMR1 | CCL28 |
|  | NDUFB6 | CLCF1 |
|  | NSMCE1 | TNFSF9 |
|  | ORC5 | BMP3 |
|  | PALD1 | IL24 |
|  | PCGF5 | GDF10 |
|  | PDIA6 | CXCL6 |
|  | PHTF1 | GDF9 |
|  | PIK3CD | IL23A |
|  | PIM2 | IL16 |
|  | PKP3 | CD70 |
|  | PLAC8 | IL5 |
|  | PLD3 | FGF9 |
|  | PNPO | IFNL1 |
|  | PPIB | TSC1 |
|  | PPIF | FGF2 |
|  | PRIM2 | IL23R |
|  | PRKAG1 | IL1G |
|  | PRKCH | SPP1 |
|  | PRPSAP1 | IL12RB1 |
|  | PSMB2 | BMP4 |
|  | PSMB3 | IL13 |
|  | PSMD13 | TPAR1 |
|  | PTPN6 | TGFB2 |
|  | PTTG1 | FAM19A2 |
|  | RABGGTA | AGIF3 |
|  | RACGAP1 | EDA |
|  | RAD17 | MIF |
|  | REPS1 | TNFSF13B |
|  | RMC1 | BMP7 |
|  | RNF14 | FGF18 |
|  | RNF167 | CCL23 |
|  | RNPS1 | CCL23 |
|  | RORA |  |
|  | RPN1 |  |
|  | RPP25L |  |
|  | RPS6KA4 |  |
|  | RSU1 |  |
|  | SATB1 |  |
|  | SCP2 |  |
|  | SELENOH |  |
|  | SEMA4A |  |
|  | SETD6 |  |
|  | SLC1A5 |  |
|  | SMIM20 |  |
|  | SMPD1 |  |
|  | SNX1 |  |
|  | SRP68 |  |
|  | SRPK1 |  |
|  | SSNA1 |  |
|  | ST13 |  |
|  | STK38 |  |
|  | SWAP70 |  |
|  | TBCB |  |
|  | TIAM1 |  |
|  | TIMM44 |  |
|  | TMC6 |  |
|  | TMEM147 |  |
|  | TMEM208 |  |
|  | TMEM223 |  |
|  | TMEM45A |  |
|  | TSPAN31 |  |
|  | TUBA3C |  |
|  | TWF2 |  |
|  | TXNL4A |  |
|  | UBE2H |  |
|  | UBE2Z |  |
|  | UBQLN1 |  |
|  | USP22 |  |
|  | USP5 |  |
|  | VAV1 |  |
|  | WTAP |  |
|  | XPNPEP1 |  |
|  | YIPF3 |  |
|  | ZFYVE19 |  |
|  | ZIK1 |  |
